# Fibroblasts facilitate lymphatic vessel formation in transplanted heart

**DOI:** 10.7150/thno.92103

**Published:** 2024-02-24

**Authors:** Hui Gong, Ting Wang, Xiaotong Sun, Yuesheng Zhang, Yichao Qiu, Wenlong Sun, Yue Zhang, Peng Teng, Yanhua Hu, Xiaosheng Hu, Liang Ma, Qingbo Xu, Haige Zhao

**Affiliations:** 1 Department of Cardiology, the First Affiliated Hospital, Zhejiang University School of Medicine, Hangzhou, China.; 2 Department of Cardiovascular Surgery, the First Affiliated Hospital, Zhejiang University School of Medicine, Hangzhou, China.; 3 Liangzhu Laboratory, Zhejiang University, 1369 West Wenyi Road, Hangzhou, China.

**Keywords:** heart transplantation, lymphatics, fibroblasts, cytokines, single cell RNA sequencing

## Abstract

**Rationale:** Lymphangiogenesis plays a critical role in the transplanted heart. The remodeling of lymphatics in the transplanted heart and the source of newly formed lymphatic vessels are still controversial, especially the mechanism of lymphangiogenesis remains limited.

**Methods:** Heart transplantation was performed among *BALB/c*, *C57BL/6J*, *Cag-Cre*, *Lyve1-CreER^T2^*;*Rosa26-tdTomato* and *Postn*(*2A-CreER^T2^-wpre-pA*)*1*;*Rosa26-DTA* mice. scRNA-seq, Elisa assay, Western blotting, Q-PCR and immunohistochemical staining were used to identify the cells and cell-cell communications of allograft heart. Cell depletion was applied to in *vivo* and in *vitro* experiments. Whole-mount staining and three-dimensional reconstruction depicted the cell distribution within transparent transplanted heart.

**Results:** Genetic lineage tracing mice and scRNA-seq analysis have revealed that these newly formed lymphatic vessels mainly originate from recipient LYVE1^+^ cells. It was found that LECs primarily interact with activated fibroblasts. Inhibition of lymphatic vessel formation using a VEGFR3 inhibitor resulted in a decreased survival time of transplanted hearts. Furthermore, when activated fibroblasts were ablated in transplanted hearts, there was a significant suppression of lymphatic vessel generation, leading to earlier graft failure. Additional investigations have shown that activated fibroblasts promote tube formation of LECs primarily through the activation of various signaling pathways, including VEGFD/VEGFR3, MDK/NCL, and SEMA3C/NRP2. Interestingly, knockdown of VEGFD and MDK in activated fibroblasts impaired cardiac lymphangiogenesis after heart transplantation.

**Conclusions:** Our study indicates that cardiac lymphangiogenesis primarily originates from recipient cells, and activated fibroblasts play a crucial role in facilitating the generation of lymphatic vessels after heart transplantation. These findings provide valuable insights into potential therapeutic targets for enhancing graft survival.

## Introduction

Heart transplantation is the most effective treatment for patients with end-stage heart failure. Although the use of immunosuppressive agents after transplantation improves the survival of transplanted hearts to a certain extent, the long-term survival of transplanted hearts is still a challenge 1. The role of the lymphatic system in modulating cardiac disease has recently become the focus of significant cardiac research 2, 3. Lymphangiogenesis is involved in the clearance of inflammatory cells from the injured tissues, which is a vital step in the resolution of inflammation and prevention of fibrotic remodeling 4-8. Lymphangiogenesis has not only been proposed to facilitate chronic rejection by promoting escape of antigen-presenting cells to regional lymph nodes and enhancing allosensitization 9, but also been considered to support heart allografts survival by lymph drainage 10. However, the process of lymphatic remodeling and the origin of newly formed lymphatic vessels in cardiac allograft is still controversial. And more importantly, the underlying mechanisms of lymphangiogenesis, such as the dominant regulating cells and how these cells lead to lymphatic vasculature formation remain limited.

Following heart transplantation, severed donor lymphatics to the heart are not anastomosed to recipient lymphatics 11. Thus, both the restoration of lymphatic drainage from heart grafts and immune responses after transplantation rely on lymphangiogenesis, a process which has not been systematically studied in cardiac transplantation. The origin of lymphangiogenesis remains a topic of ongoing debates. It has been suggested that donor cells are the main sources of lymphatics in some mouse intraperitoneal cardiac transplant models 12, 13, while the lymphatic vessels detected in quality lesions of human cardiac allografts are considered to be from the recipient 11. These conflicting results may be attributed to the limitations in experimental design and technical problems. Thus, what contributes to the origin of lymphangiogenesis is still one of the questions that needs further investigation.

A number of signaling pathways have been shown to contribute to lymphangiogenesis during embryonic development and pathological processes. The most well-studied signaling pathway involves the secreted lymphangiogenic glycoproteins, VEGFC and VEGFD, which act directly on the lymphangiogenic receptor, VEGFR3 (Flt-4) 14, 15. It has been suggested that macrophages and CD4^+^ T cells are the major sources of the predominant lymphangiogenic VEGFR3 ligand VEGFC in a rat cardiac allograft model 12. Recently, the study from our laboratory revealed that fibroblasts could be involved in lymphangiogenesis in mouse vessel grafts 16. Since in addition to cardiomyocytes and endothelial cells, fibroblasts are another important cellular component of the heart 17, the role of fibroblasts in the lymphatic formation in the transplanted heart may be overlooked. Therefore, it is of interest to investigate whether cardiac fibroblasts and which underlying molecular mechanisms could be involved in the regulation of lymphangiogenesis post-transplantation.

In this study, our objective was to investigate the characteristics, origins, and potential mechanisms of lymphatic formation and lymphangiogenesis in a mouse heterotopic cervical heart transplant model. Our findings demonstrated that lymphangiogenesis predominantly occurs in the myocardium of cardiac allografts. The majority of these newly formed lymphatic vessels are derived from the recipient, with LYVE1^+^ cells playing a significant role in the development of LECs in allograft hearts. Additionally, we discovered that fibroblasts become activated (without expressing α-SMA) following transplantation. These activated fibroblasts act as the primary regulatory cells for LECs and facilitate the formation of lymphatics through multiple pathways, including the VEGFD/VEGFR3, MDK/NCL, and SEMA3C/NRP2 ligand-receptor pairs. We further demonstrated that deleting activated (POSTN^+^) fibroblasts or knocking down VEGFD and MDK in activated fibroblasts of cardiac allografts can alleviate lymphangiogenesis after heart transplantation.

## Material and Methods

### Mouse Generation and Genotyping

All animal studies conformed to the Guidelines for the Care and Use of Laboratory Animals published by the US National Institutes of Health (NIH Publication Eighth Edition, 2011) and were approved by the Institutional Animal Care and Use Committee as well as Zhejiang University School of Medicine (Hangzhou, China). Mouse strains used in this study were as follows: *BALB/c* mice, *C57BL/6J* mice, *Cag-Cre* mice (*C57BL/6J* background),* Lyve1-CreER^T2^* mice (*C57BL/6J* background), *Rosa26-tdTomato* mice *(B6.129(Cg)-Gt(ROSA) 26Sortm4 (ACTB-tdTomato,-EGFP)Luo/J)*, *Rosa26-DTA (Gt(ROSA)26Sortm1(DTA)Jpmb/J)* mice, and* Postn*(*2A-CreER^T2^-wpre-pA*)*1* (*C57BL/6J* background) mice were all purchased from Shanghai Biomodel Organism Co.,Ltd.* Lyve1-CreER^T2^*; *Rosa26-tdTomato* mice were generated by crossing *Lyve1-CreER^T2^* mice and *Rosa26-tdTomato* mice. *Postn*(*2A-CreER^T2^-wpre-pA*)*1*; *Rosa26-DTA* mice were generated by crossing *Postn*(*2A-CreER^T^2-wpre-pA*)*1* mice and *Rosa26-DTA* mice. All transgenic mice were bred on a *C57BL/6J* background and raised in the Laboratory Animal Center of Zhejiang University for at least a week before use. Animals were fed on a standard laboratory diet with free access to food and water and kept in a humidity (65-70%) and temperature (22 ± 1°C) and controlled room, with a 12-h light-dark cycle. Genomic DNA from mice tail tissue was identified by proteinase K lysed, isopropanol precipitated and 70% ethanol washed and then prepared for polymerase chain reaction (PCR). All mice were randomly allocated to the control group and experimental groups. The number of mice used in each experiment was clarified in the figure legends. The adult recipient mice (8-12 weeks) and female donor mice (3 weeks) were used as the start point (0 week) for each experiment.

For *Lyve1-CreER*^T2^;* Rosa26-tdTomato* mice and *Postn*(*2A-CreER^T2^-wpre-pA*)1; *Rosa26-DTA* mice, tamoxifen (Sigma, T5648, 20 mg/mL) dissolved in corn oil (Sigma, C8267) was administered by gavage at the start point with the dose of 0.15 mg/g body weight every three days for two weeks to label tdTomato. Cyclosporin A (APExBIO, B1922) was administered continuously to the recipient mice from one week before surgery to the day of allograft harvest. In a nutshell, 0.5 g Cyclosporin A was dissolved in 50 mL absolute ethyl alcohol and stored at -30°C. The storage solution was diluted with double distilled water (ddH_2_O) in a ratio of 1: 50 before being given to mice. At the indicated time, the mice were sacrificed and the cardiac allografts were harvested for further study.

VEGFR3 inhibitor SAR131675 (Selleck, S2842) was first dissolved with DMSO as stock solution (100mg/mL), then the diluted solution (30% PEG300+5% Tween 80+65% ddH_2_O) (DMSO: Sigma, 472301; PEG300: Selleck, S6704; Tween 80: Selleck, S6702) was administered to the recipient mice by gavage with the dose of 1mg/200μL/25g body weight every day for the first three days after heart transplantation and every three days till the cardiac palpitation stopped, mice treated with 5%DMSO diluted in 30% PEG300+5% Tween 80+65% ddH_2_O solution were used as control group.

### Heterotopic Cervical Heart Transplantation

As previously reported, we performed heterotopic cervical heart transplantation 18, 19. In brief, to induce global anesthesia, mice were placed in an anesthesia induction box, which was connected to the vaporizer with 3.0 Vol% isoflurane and a 100% oxygen flow of 1 L/min. Then the inferior vena cava of donor mice was injected with a saline solution containing 100 I.U. heparin sodium. The inferior and superior vena cava were ligated after the thoracic opening, then the ascending aorta and pulmonary artery trunk were isolated and cut close to the heart just before branching. Finally, the rest of pulmonary vessels were tied off and the heart was removed and immersed in ice-cold saline. In the following, the recipient was narcotized with isoflurane mentioned above followed by intraperitoneal injection of ketamine (100 mg/Kg) and then was placed on a heated pad set to 37°C. With microsurgery forceps, the connective tissues around the external jugular vein and carotid artery were meticulously ridded of, thus vessels were exposed and transected for connecting with the donor's heart. Pulling the aorta of the graft over the cuff with the carotid artery and pulling pulmonary trunk over the cuff with the external jugular vein, then we fixed the ligation with an 8-0 silk ligature around the circumference. Coronary revascularization after heart transplant was confirmed, following securing the anastomosis. 1,2 or 4 weeks after transplantation, cardiac allografts were harvested for further analysis following the perfusion of sterile phosphate buffered saline (PBS).

### Bone Marrow Transplantation

As alluded to above, a *C57BL/6J* mouse and a* Cag-Cre*;* Rosa26-tdTomato* mouse were treated with the bone marrow transplantation. First of all, the recipient mice (8-12 weeks male *C57BL/6J* mice) were exposed at the dosage of 8.5-9 Gy for 6-hours whole-body irradiation. The donor mice (8-12 weeks male *Cag*-*Cre*; *Rosa26-tdTomato* mice) were used to harvest bone marrow cells. In a nutshell, the donor mice' femurs and tibias were isolated and immersed in ice-cold RPMI 1640 medium (Biological Industries, 01-100-1A). Afterwards, 1-1.5 mL RPMI 1640 medium was pumped into the cavities of isolated bones. To filter clumped cells and other contaminants, collected bone marrow cells were passed through 40 μm cell strainers (Falcon, 352340). After centrifuging, RPMI 1640 medium was used to resuspend the bone marrow cells. Six hours after irradiation, irradiated mice received an injection at the dosage of 5×10^6^ bone marrow cells per mouse through the tail vein to form chimeric mice. The efficiency of chimeric mice was confirmed by flow cytometry to evaluate the percent of tdTomato^+^ cells in peripheral blood of recipient mice.

### Heart Tissue Cell Dissociation

In brief, mice were anaesthetized with 3% isoflurane and killed by cervical dislocation. Hearts were quickly excised and fresh cardiac tissues were divided into 1 mm^3^ pieces and digested by 0.5 mg/mL liberase solution (Roche, 05401020001) at 37 °C for 20 minutes to generate single-cell suspensions. Afterwards, heart tissue pieces were gently shaken in a 37 °C shaker to guarantee complete digestion of tissues. After the digestive process was completed, the supernatant medium was harvested and the same volume of Dulbecco's Modified Eagle's medium (DMEM, ATCC, 302002) containing 10% fetal bovine serum (FBS, Gibco, 10099141) was added to abort the reaction. A New liberase solution was then added to the surplus tissue fragments for further digestion. To obtain single cell suspension, the mixture of supernatant and neutralization solution was subsequently passed through a 40-μm cell strainer. The collected cells were centrifuged for 5 minutes at the speed of 300 g/min at 4°C. Subsequently, cell pellets were suspended in relevant neutralization buffer for further experiments.

### Flow Cytometric Analysis

For bone marrow analysis, cells were gathered and treated with red blood cell lysis buffer (Biogems, 64010-00) to lyse red blood cells. Single cells suspensions were obtained through filtration with a 40-μm cell strainer. Thus, the composition of tdTomato^+^ cells was assessed. Using Hoechst 33342 (Invitrogen, H3570, 1:1000), isolated cells were stained. As directed by the manufacturer, fluorochrome-conjugated antibodies (1 μg per 10^6^ cells) were used to label the cells extracted from mice cardiac allografts. Conjugated-antibodies used include, CD45-percp-cy5.5 (BD pharmingen, 550994, 1:100) for 30 min. Then, to exclude dead cells, the stained cells were rinsed in PBS before being dyed for 20 minutes with LIVE/DEAD ™ Fixable Near-IR Dead Cell Stain Kit (Invitrogen, L34975 1:1000). Afterwards, cells were washed with PBS and re-suspended with PBS containing 5% BSA. Using a CytoFLEX LX flow cytometry equipment, fluorescence-tagged cells were evaluated, and data were analyzed by CytExpert in accordance with protocols. The cell sorting process was performed using BD LSR Fortessa II flow cytometer (BD Biosciences), and FlowJo v10 software (BD Biosciences, USA) was used for subsequent analysis.

### Single-cell RNA-sequencing, Quality Control and Cell Clustering

Allograft cell pellets were re-suspended with PBS following complete digestion. Using red blood cell lysis buffer, red blood cells were eliminated. Cells were stained with Hoechst 33342 (1:1000) for 20 mins and then LIVE/DEAD ™ Fixable Near-IR Dead Cell Stain Kit (1:1000) for 20 mins. Using a BD FACS ARIA II Flow Cytometer (BD Biosciences), single nucleated live cells (Dead Cell Stain^-^ & Hoechst^+^) were sorted into PBS with 1% BSA, followed by PBS washing and 5% BSA re-suspension. With respect to single nucleated live CD45^-^ cells of cardiac allografts, Dead Cell Stain^-^ & Hoechst^+^ & CD45^-^ cells were centrifuged for 8 minutes at 400 g/min speed at 4°C. Collected samples were then subjected to scRNA-seq.

A Chromium Single Cell 3' Reagent Kit v2 and v3 chemistry (10X Genomics) was used to generate a single cell library using the standard protocol. The library was generated and sequenced on a NovaSeq platform (Illumina) using paired-end 150 bp (PE 150) sequencing strategy. scRNA-seq raw data were skillfully processed by Cell Ranger (version 6.0.0). At a word, matching reads and feature-barcode matrices were generated from FASTQ files, which were managed by “Cellranger mkfastq”. For the sake of matching reads with the mouse reference to calculate the number of barcode and UMI, “Cellranger count” was processed. To add up sequencing data from different libraries to the same sequencing depth (30M/cell), we used “Cellranger aggr”. After cumulation of the samples, deeper analysis and visualization were performed with R (version 4.0.5) package Seurat (version 4.1.0). For cardiac allograft analyzing, low quality cells were defined as “cells with < 400 genes/cell or > 6000 genes/cell and > 10% mitochondrial transcript presence/cell', which were left out of downstream analyses. According to the expression of hemoglobin related genes, red blood cells were also excluded. The R package DoubletFinder (version 2.0.3) was also used to filter Doublet cells. Then, the log normalized gene expression data to a scale factor of 10000 was regressed on the number of molecules detected per cell (nUMI). Subsequently, principal component analysis (PCA) was performed to identify highly variable genes, besides, the first 30 principal components were gathered for further analysis. “FindAllMakers” was performed to identify the marker genes upregulated in each cluster (min.pct = 0.25, logfc.threshold = 0.25). Seurat functions “DimPlot”, “FeaturePlot”, “DotPlot”, “VlnPlot” and “DoHeatmap” were used for visualizing data, as well as R package ggplot2.

### Gene Ontology Enrichment Analysis

By using R package 'clusterProfiler', top 200 differentially expressed genes (DEGs) in each cluster compared to the rest population were applied to gene ontology (GO) term enrichment.

### Gene Sets Score

The Seurat function “AddModuleScore” was devoted to perform gene set analyses on gene sets of interest. The expression of genes within each gene set in each cell was used to calculate the score for each cell. The gene sets of interest were obtained from previously published studies, in addition, the gene ontology resource datasets.

### Cell Communication Analysis

Cellchat was recently created to analyze and visualize intercellular communications derived from scRNA-seq data, which was a convenient R package. By using Cellchat, we identified and visualized specific intercellular signaling communications by signaling patterns and the single ligand-receptor pair in our datasets.

### Histology and Immunofluorescence Staining

As for histology analysis, mice were perfused with ice-cold PBS followed by 4% paraformaldehyde. Thus, cardiac tissues were fixed in 4% paraformaldehyde overnight. Fixed tissues were then paraffin embedded and sectioned at 5 μm. Sirius Red staining and Masson staining were used to visualize collagen deposition of heart tissues, then the software ImageJ was used to quantify the fibrosis level as a positive area. The heart structure was depicted by Hematoxylin and eosin staining.

For immunofluorescence staining, tissues were washed three times with PBS before being fixed in 4% PFA at 4 °C overnight. The fixed tissues were then dehydrated in a 30% sucrose solution at 4 °C overnight until fully penetrated. Afterwards, tissues were embedded in optimum cutting temperature (O.C.T., Sakura, 4583) and cut into 8-µm sections by a Cryostat (Leica CM1950). Cryosections were aired-dried for 30 minutes at room temperature, blocked with 5% donkey serum (Solarbio®, SL050) containing 0.1% Triton X-100(Sigma, X-100) in PBS at room temperature for 1 h, stained sections with primary antibodies overnight at 4 °C, and then incubated with or without Alexa Fluor-conjugated secondary antibodies (Invitrogen, 1:500) at room temperature for 1 h. Then, DAPI (Servicebio, G1012) was stained for 15 min at room temperature before being mounted with an anti-fade mounting medium (Servicebio, G1401). Primary antibodies were applied in this study as listed: LYVE1 (abcam, ab14917, 1:300), VEGFR3 (R&D Systems, AF349, 1:50), tdTomato (Rockland, 600-401-379, 1:500), Periostin (R&D system, AF2955, 1:50), Collagen Type I (Proteintech, 67288-1-Ig, 1:100 ), DDR2 (discoidin domain receptor 2, R&D Systems, MAB25381, 1:100), PDGFRα (R&D Systems, AF1062, 1:50), PCNA (Abclonal, A0264, 1:500), KI67 (abcam, ab16667, 1:500), VEGFD (Proteintech, 26915-1-AP, 1:50), Midkine (Proteintech, 11009-1-AP, 1:50), SEMA3C (R&D Systems,MAB1728, 1:50), PTN (abcam, ab79411, 1:50), NRP2 (Neuropilin-2, CST, D39A5, 1:50), NCL (nucleolin, Santa cruz, sc-8031, 1:50), Vimentin (abcam, ab8978, 1:100), Fibronectin (abcam, ab2413, 1:50), CD31 (R&D, AF3628, 1:50), CD31 (abcam, ab28364, 1:50), SMA-FITC (Sigma, F3777, 1:300), p-AKT(Santa cruz, sc-514032, 1:50), p-ERK (Santa cruz, sc-7383, 1:50), CD45 (R&D Systems, AF114, 1:50), TNF-α(Proteintech, 60291-1-Ig, 1:50), IL1β (Abclonal, a19635, 1:50), IL6 (Proteintech, 66146-1-Ig, 1:50). Secondary antibodies were used as listed: donkey anti-rabbit IgG Alexa Fluor 488 (Invitrogen, A-21206, 1:500), donkey anti-rabbit IgG Alexa Fluor 555 (Invitrogen, A-31572, 1:500), donkey anti-rat IgG Alexa Fluor 488 (Invitrogen, A-21208, 1:500), donkey anti-mouse IgG Alexa Fluor 488 (Invitrogen, A-21202, 1:500), donkey anti-mouse IgG Alexa Fluor 555(Invitrogen, A-31570, 1:500), donkey anti-mouse IgG Alexa Fluor 647(Invitrogen, A-32787, 1:1000),donkey anti-goat IgG Alexa Fluor 488 (Invitrogen, A-11055, 1:500), donkey anti-goat IgG Alexa Fluor 555 (Invitrogen, A-32816, 1:500), donkey anti-goat IgG Alexa Fluor 647 (Invitrogen, A-21447, 1:500). A secondary antibody-only control was used to validate specificity of antibodies and to remove the background signal in immunofluorescence staining.

A Nikon A1 Ti confocal microscope and an Olympus FV3000 confocal microscope were used to acquire cryosection and cell staining images, further analyzed by relevant software. To avoid biasing, the captured regions were chosen at random. For immunostaining images quantification, at least 5 random fields of view containing the entire heart were counted and the mean values were used as the data point for each mouse sample. When performing immunostaining and analyzing the data, the researchers were blinded to the different groups.

### Whole-organ Clearing

For whole heart-clearing and 3D reconstruction, mice were sacrificed and perfused with 20 mL PBS and 20 mL 4% PFA via the heart's left ventricle. Overnight, the harvest allografts were fixed in 4% PFA. To remove residual PFA, the fixed tissues were washed with PBS for 3 times, more than 2 hours at a time. The 50% CUBIC-L (1:1 mixture of CUBIC-L and water) was penetrated into the fixed tissues for over 6 hours. The delipidation procedure was in CUBIC-L carried out for over 5 days with gentle shaking at 37 °C, and the CUBIC-L was refreshed on a daily basis. After decolorization and delipidation procedure, allografts were washed 3 times in PBS for more than 2 hours a time. This procedure was followed by primary and secondary antibody incubation, with each incubation period lasting 7 days at 4 °C. The antibodies used here were the same as indicated for immunofluorescence staining. After antibody incubation, tissues were washed with PBS for 3 times, each lasted over 2 hours. The tissues were then incubated with 50% CUBIC-R and 100% CUBIC-R [45% (wt/wt) antipyrine (TCIchemicals, D1876), 30% (wt/wt) nicotinamide (TCIchemicals, N0078), 0.5% (vol/ vol) N-butyldiethanolamine in ddH_2_O] sequentially for transparency. The processed hearts were imaged by a light sheet microscope Z1 (Zeiss, MC-LM1) and further reconstructed using the Imaris 9.0.1 (Bitplane, Switzerland) software.

### RNA Extraction, Reverse Transcription, Quantitative Polymerase Chain Reaction

Total RNA was isolated by the TRIzol™ reagent (Thermo Fisher Scientific, 15596018) and cDNA was reverse-transcribed according to the manufacturer's instructions using a RevertAid RT Reverse Transcription Kit (Thermo Fisher Scientific, K1691). All cDNA obtained was diluted with DEPC water to a working concentration of 5ng/μL and then stored at -20 ºC. Using the TB Green® Premix Ex Taq™ II (Tli RNaseH Plus) (Takara, RR820A) with the CFX384 Real-Time System (BIO-RAD, CA, USA), the relative mRNA levels of target genes were measured. The expression level of specific genes was normalized using Gapdh or 18sRNA.The Nanodrop ND-1000 spectrophotometer was applied for controlling sample quality. The quantitative qPCR data of mRNA products were analyzed by 2- ΔΔCT method. The expression level was normalized to 18s or Gapdh (mRNA products) using ExpressionSuite (Applied Biosystems) software. The PCR procedure protocol was as follows: 95 °C for 10 min; followed by 40 cycles of 95 °C for 15 sec, 60 °C for 30 sec, and 72 °C for 30 sec; followed by a final 60 °C for 1 min. Primers used in this study are listed as follows:

18s RNA Forward 5'-CGTCTGCCCTATCAACTTTCG-3'

18s RNA Reverse 5'-GCCTGCTGCCTTCCTTGG-3'

Gapdh Forward 5'-AGGTCGGTGTGAACGGATTTG-3'

Gapdh Reverse 5'-TGTAGACCATGTAGTTGAGGTCA-3'

Vegfd Forward 5'-CTCCACCAGATTTGCGGCAACT-3'

Vegfd Reverse 5'-ACTGGCGACTTCTACGCATGTC-3'

Mdk Forward 5'-AAGGTGCCCTGCAACTGGAAGA-3'

Mdk Reverse 5'-GCATTGTACCGCGCCTTCTTCA-3'

Sema3c Forward 5'-CTGATAGTCCGCATAGGCACTG-3'

Sema3c Reverse 5'-GGCAGAGCTATTGGTAGGAAGG-3'

Ptn Forward 5'-ATGTCGTCCCAGCAATATCAGC-3'

Ptn Reverse 5'-CCAAGATGAAAATCAATGCCAGG-3'

Flt4 Forward 5'-AGACTGGAAGGAGGTGACCACT-3'

Flt4 Reverse 5'-CTGACACATTGGCATCCTGGATC-3'

Ncl Forward 5'-ATTACACCAGCCAAAGTCATTCC-3'

Ncl Reverse 5'-TGGCAGCTCCCTTTTTACCAG-3'

Nrp2 Forward 5'-GGTGAAGATTGGATGGTCTACCG-3'

Nrp2 Reverse 5'-TGAACCGAGTCAGCAGTGGCAT-3'

Lyve1 Forward 5'-ACCAGGTAGAGTCAGCGCAGAA-3'

Lyve1 Reverse 5'-CAGGACACCTTTGCCATTCTTCC-3'

Postn Forward 5'-ACGGAGCTCAGGGCTGAAGATG-3'

Postn Reverse 5'-GTTTGGGCCCTGATCCCGAC-3'

Ddr2 Forward 5'-CTGTGGGAGACCTTCACCTT-3'

Ddr2 Reverse 5'-TAGATCTGCCTCCCTTGGTC-3'

Pdgfra Forward 5'-GGACTTACCCTGGAGAAGTGAGAA-3'

Pdgfra Reverse 5'-ACACCAGTTTGATGGATGGGA-3'

Col1a1 Forward 5'-GCTCCTCTTAGGGGCCACT-3'

Col1a1 Reverse 5'-CCACGTCTCACCATTGGGG-3'

Col3a1 Forward 5'-CCTGGCTCAAATGGCTCAC-3'

Col3a1 Reverse 5'-GACCTCGTGTTCCGGGTAT-3'

Fn1 Forward 5'-ATGTGGACCCCTCCTGATAGT-3'

Fn1 Reverse 5'-GCCCAGTGATTTCAGCAAAGG-3'

### Western Blot Analysis

Total proteins were extracted from cells and tissues with RIPA lysis buffer (Beyotime, P0013B) supplemented with phosphatase inhibitor tablets (Roche, 04906837001) and protease inhibitor tablet (Roche, 05892970001). The Enhanced BCA Protein Assay Kit (Beyotime, P0012) was used to assess the protein concentration, which was then adjusted to an equal concentration level in accordance with the manufacturer's instruction. The same amount of sample protein lysates was used in the standard western blot procedure to compare the expression of target proteins in different conditions. The following were the primary antibodies used in this experiment: anti-GAPDH (GNI, GNI4310-GH, 1:5000), anti-β-actin (Sigma, A5441), anti-Fibronectin (abcam, ab2413, 1:1000), anti-Periostin (R&D Systems, AF2955, 1:1000), anti-VEGFD (Proteintech, 26915-1-AP, 1:50), anti-Midkine (Proteintech, 11009-1-AP, 1:50), anti-SEMA3C (R&D Systems,MAB1728, 1:50), anti-VEGFR3 (R&D Systems, AF349, 1:50), anti-Nucleolin (Santa cruz, sc-8031, 1:50), anti-NRP2 (Neuropilin-2, CST, D39A5, 1:50), Vimentin (abcam, ab8978, 1:100), LYVE1 (abcam, ab281587, 1:100). The strips were then incubated with secondary antibody. As internal references, anti-GAPDH and anti-β-actin were used to calculate relative abundance of proteins.

### Quantification of MDK, VEGFD, SEMA3C and PTN by ELISA

The concentration of MDK, VEGFD, SEMA3C and PTN in mice blood serum underwent heart transplantation were detected by enzyme-linked immunosorbent assay (ELISA) according to the protocol. All the results were expressed as ng/mL. The ELISA kits used in our research were as follows: Mouse MDK ELISA Kit (405322V, mlbio), mouse VEGFD ELISA Kit (076303V, mlbio), mouse SEMA3C ELISA Kit (110158V, mlbio), mouse PTN ELISA Kit (188540V, mlbio).

### Colocalization Analysis

We evaluated colocalization in immunofluorescence staining to elucidate cellular functions of proteins, which is typically determined by comparing the distribution of one fluorescently labeled molecule with that of another. The result was reflected in the plot profile by the software ImageJ. Initially, the image to be analyzed was opened in ImageJ, then the channels were split following channels merged. Afterwards, the area of interest was determined. Then, we selected “Analyze - Plot Profile” to demonstrate each channel's fluorescence intensity distribution along the region of interest. Finally, the data were exported to Origin to obtain the curve of different fluorescence intensities along the rectangular trajectory.

### siRNAs Transfection, LEC Migration, Proliferation and Tube Formation Assay in vitro

Transfection was performed on fibroblasts and lymphatic endothelial cells that were 70% confluence. Fibroblasts were transfected with 50 nM siCtrl, siVEGFD, siMDK, siSEMA3C, siPTN (purchased from GENERAL BIOL), while lymphatic endothelial cells were transfected with 50 nM siCtrl, siFlt4, siNrp2, siNcl (purchased from GENERAL BIOL) using a Lipofectamine RNAiMAX transfection reagent (Invitrogen, 13778150) according to the manufacturer's instructions. The medium was changed to a complete medium after 6-hour incubation.

The chemotaxis of substances secreted or metabolized by fibroblasts to LECs can be studied by seeding LECs in the upper chamber (24-well Transwell chamber, Corning, 3422) while fibroblasts in the lower chamber, which were transfected and then cultured for 24 hours. After 24 hours of cocultivation, the culture solution in the well was discarded. Then the transwell chambers were washed twice with calcium-free PBS, fixed with methanol for 30 minutes and properly air dried. After being tinted with 0.1% crystal violet for 20 minutes, the unmigrated cells of the upper layer were gently wiped off with a cotton swab, and washed with PBS for three times. The cells were observed and counted in five visual fields under microscope.

As for the cell proliferation experiment, about 5x10^3^ LECs were added to 96-well plates containing the culture supernatant of transfected fibroblasts as the nutrient medium. Each siRNA represented one experimental group with 7-8 multiple wells in each group. At the same time, blank wells with no cells needed to be created. Then the cells were cultured for 48 hours in an incubator with 37 °C and 5% CO_2_. We added 10 uL of CCK-8 solution (Beyotime, C0038) into each well slowly by immersing the pipette tip into the culture solution, especially, paid attention to avoiding bubbles. Then, the cells were incubated with CCK-8 in an incubator at 37 °C. At last, the OD value was measured at 1, 2, 4, 6 h respectively, using 450 nm wavelength with the microplate reader.

15-well chamber slide's chambers (ibidi, 81506) were coated with 50μL of Matrigel (Corning, 356234) at 37°C for 30 minutes. 2x10^4^ LECs with different culture supernatant from different transfected fibroblasts were then seeded on top of the substrate for 4 to 6 hours. Rearrangement of the cells and formation of capillary-like structures were then examined, with images captured using the imaging microscope (Zeiss Axioplan 2).

### Lenti-virus Infection in Mouse Heart

The siRNA for *Mdk* or *Vegfd* in an intron sponge was chemically synthesized to create postn promoter-driven sponge expression lentivirus cis cassettes 20. Lentivirus plasmids used as control were produced by replacing siRNA with sense sequence of *Mdk* or *Vegfd*.

Briefly, 50 μL viral vectors (1x10^9^ cfu/mL) were injected at four points between the two muscle layers of the apex of the beating heart prior to dissociation from the donor mouse. To ensure sufficient virus infection, an additional injection of 50 μL virus was administrated through a blunt-tip syringe into the heart via the aortic arch. The virus-infected heart was subsequently placed in iced saline for one hour before transplantation.

### Human Cardiac Tissues and Blood Serum Collection

Human cardiac tissues and blood serum were obtained from patients with or without heart transplantation surgery between December 2020 and November 2022 at the First Affiliated Hospital of Zhejiang University School of Medicine (Hangzhou, China). Individuals were age and gender matched for the control and heart transplantation groups. All studies were authorized by the Research Ethics Committees of the First Affiliated Hospital of Zhejiang University School of Medicine (institutional review board approval No. 2021/330). All patients provided the written, informed consent for the collection of samples. All procedures were ethically approved according to the principles expressed in the Declaration of Helsinki and is compliant with the ISHLT statement on transplant ethics. Human cardiac tissues were fixed with 4% paraformaldehyde (PFA, Servicebio, G1101) and collected for further paraffin-embedded sections.

### Statistical Analysis

**Figure 1:**
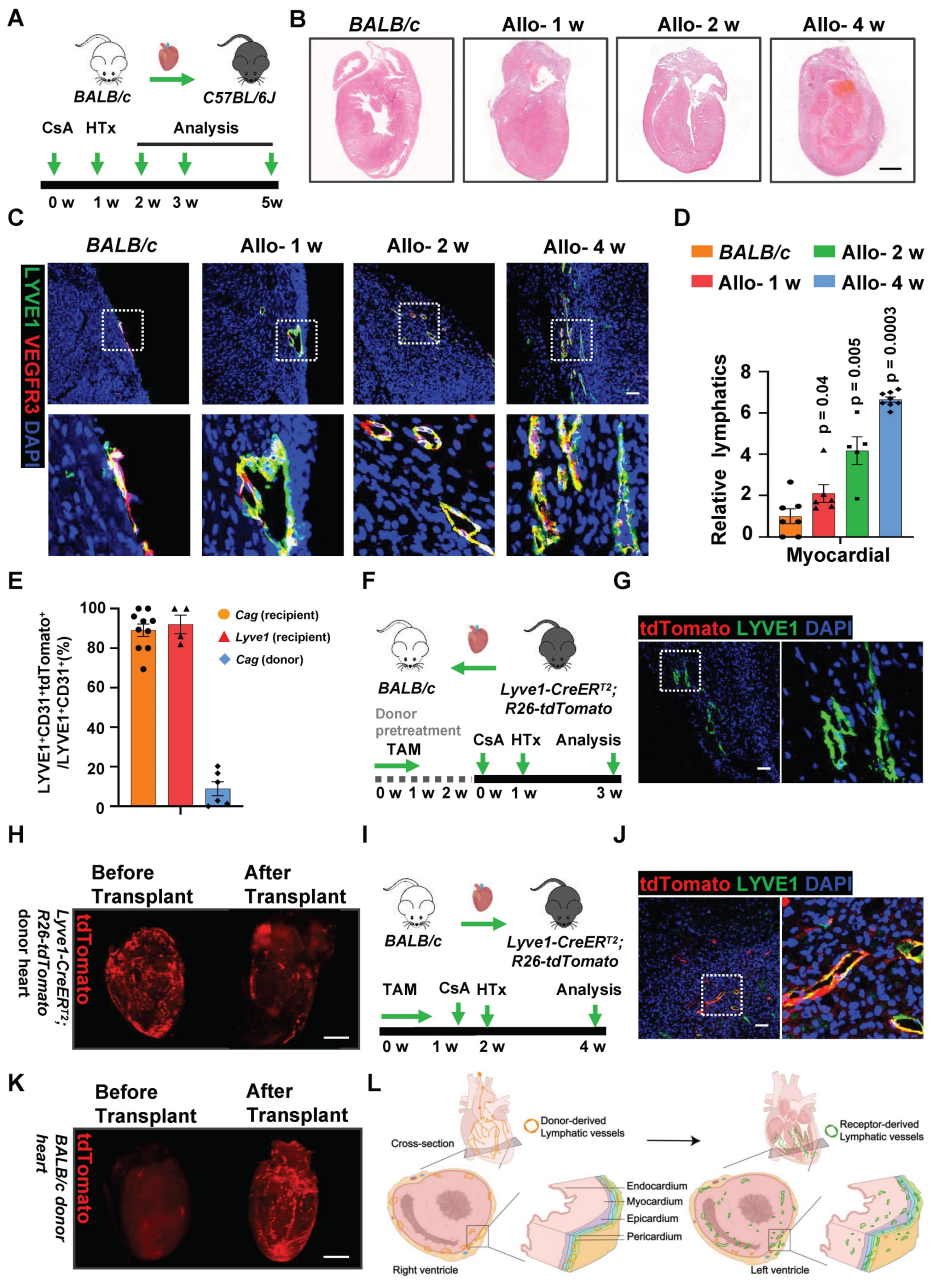
** Recipient Derived LYVE1^+^ Cells Reconstruct Lymphatic Vessels in Cardiac Allograft. A**, Schematic of mouse heart transplant (allograft) model. **B**, HE staining of heart in normal *BALB/c* mouse (control) and indicated time points (1 week, 2 and 4 weeks) allografts after heart transplanted mouse. **C**, Immunostaining of lymphatic vessels (LYVE1^+^VEGFR3^+^) in the heart of control and allograft hearts. The images within the dashed box are displayed in the second row. **D**, Quantification of lymphangiogenesis showed in (**C**), the relative lymphatics in the figure are calculated based on the lymphatic vessel density of *BALB/c* mouse (control) donor heart as the criterion (1.0). Number of mice in each group: n = 7(*BALB/c*), n = 6 (Allo-1 w) , n = 5 (Allo-2 w), n = 8 (Allo-4 w), one-way ANOVA test. **E**, Quantification of origin of lymphatic vessels in allograft heart 2 weeks after transplantation. Number of mice in each group: n = 10 (Cag recipient), n = 4 (Lyve1 recipient), n = 6 (Cag donor). **F**, Schematic of heart transplantation model: donor heart from *Lyve1*-*CreER^T2^R26-tdTomato* mouse was transplanted into *BALB/c* recipient mouse. **G**, Immunostaining of lymphatic vessels in allograft heart 2 weeks after transplantation which was described in (**F**), tdTomato^+^ cells indicate the cells derived from donor *Lyve1*-*CreER^T2^R26-tdTomato* mouse, LYVE1^+^ cells indicate lymphatic endothelial cells. The image within the dashed box is displayed on the right. **H**, Whole-mount staining of tdTomato in the heart from normal donor *Lyve1*-*CreER^T2^R26-tdTomato* mouse (left), and allograft heart from donor *Lyve1*-*CreER^T2^R26-tdTomato* mouse 2 weeks after transplantation (right). **I**, Schematic of heart transplantation model: donor heart from *BALB/c* mouse was transplanted into *Lyve1*-*CreER^T2^R26-tdTomato* recipient mouse. **J**, Immunostaining of lymphatic vessels in allograft heart 2 weeks after transplantation which was described in (**I**), tdTomato^+^ cells indicate the cells derived from recipient *Lyve1*-*CreER^T2^R26-tdTomato* mouse, LYVE1^+^ cells indicate lymphatic endothelial cells. The image within the dashed box is displayed on the right. **K**, whole-mount staining of tdTomato in the heart from normal *BALB/c* mouse (left), and in allograft heart 2 weeks after donor *BALB/c* heart was transplanted into recipient *Lyve1*-*CreER^T2^R26-tdTomato* mouse (right). **L**, Schematics of lymphatic vessel remodeling in mouse allograft hearts. Scale bars, 1 mm (B, H and K), and 100 µm (C, G and J). Allo: allograft; CsA: cyclosporine A; TAM: tamoxifen; HTx: heart transplant.

GraphPad Prism 9.0 was used to draw statistical images and perform comparative analyses in Figure 1D, 3I, 3K, 4G, 4I, 5D, 5F, 5H, 5I, 6D, 6F, 6H, 7A, 7D, 7H, 7K, and Supplemental Data Figure 1E, 7F, 7H, 7J, 8C, 8D, 8G, 10B, 10E, 10G, 10I, 10J, 11A, 11B, 11E, 11G, 11H, 12A, 12B, 12D, 13B, 13D, 13G. Numbers (n) referred to the number of mice or patients used in the experiments, as indicated in corresponding figure legends. Each result was presented as mean ± SEM. The normality of the data of animal and human was determined by D'Agostino-Pearson omnibus test or Shapiro-Wilk test. Then data that passed the normality and lognormality tests were tested by Ordinary one-way ANOVA tests in Figure 1D, 4I, 5F, 7D, 7E, 7F and Supplemental Data Figure 1E, 11E, 11G, 11H, while unpaired two-tailed t test in Figure 3I, 4G, 5D, 5H, 6D, 6F, 6H, 7A, 7H, 7K, and Extended Data Figure 7F, 7H, 7J, 8C, 8D, 8G, 10B, 10E, 10G, 10I, 10J, 11A, 11B, 11G, 12A, 12B, 12D, 13B, 13D, 13G with or without Welch's correction. The Mann-Whitney test or Kolmogorov-Smirnov test was used to analyze the data that did not pass the normality and lognormality tests in Figure 3K. P value < 0.05 was regarded as statistically significant. Images with high image quality that were most accurately matched the quantitative analysis were shown. Each independent experiment was repeated three times. Each scRNA-sequencing sample of cardiac allografts included 5-8 independent mouse individuals.

## Results

### Recipient LYVE1^+^ Cells Reconstruct Lymphatic Vessels in Mouse Cardiac Allografts

A mouse heterotopic cervical heart transplant model (**Figure S1A-B**) was applied here to investigate lymphangiogenesis. *BALB/c* mice hearts were transplanted into *C57BL/6J* recipient mice, cyclosporine A (CsA) was given to the recipient mouse in order to avoid acute rejection. Cardiac allografts were harvested for further analysis 1 week, 2 and 4 weeks after transplantation (**Figure 1A**). Cardiac shape and size were compared between control hearts (normal *BALB/c* hearts) and allograft hearts. No obvious heart dilation or shrinkage until 4 weeks after transplantation (**Figure S1C**). Increased cell infiltration was observed in the heart after transplantation (**Figure 1B, Figure S1D-E**), demonstrating that the cardiac environment was altered and could be in a proinflammatory state, which was coincident with previous findings 12, 21. We next investigated the lymphangiogenesis in cardiac allografts. Heart sections for immunostaning against LYVE1 and VEGFR3 indicated that lymphangiogenesis was primarily presented in the myocardium of mouse allograft hearts (**Figure 1C-D**).

To further investigate the lymphatic cellular origin, lineage-tracing experiments using a *Cag*-*Cre* mice, crossed with a *R26-tdTomato* reporter line were performed. Both strains of mice were of *C57BL/6J* genetic background. Theoretically, all *Cag*-*Cre* expressing cells would be labeled with tdTomato. To test the labeling effect of CAG, we detected tdTomato and DAPI in major organ slices. Co-expression of tdTomato and DAPI was widely observed in the aorta, heart, kidney, liver and spleen (**Figure S2A**). These data demonstrated that the tdTomato^+^ cells faithfully represented CAG cells.

In order to assess the contribution of donor-derived cells in lymphangiogenes after heart transplantation, we transplanted *Cag*-*Cre;R26-tdTomato* donor hearts into *BALB/c* recipients. About 8.9% recombination of tdTomato within LYVE1^+^ lymphatic vessels were evident with tdTomato^+^LYVE1^+^/LYVE1^+^ lymphatic vessels (**Figure 1E, Figure S3A-B**), indicating donor-derived cells were not the main source of LECs after heart transplant. Then we transplanted *BALB/c* donor hearts into *Cag*-*Cre*;R26-tdTomato recipients, and found most (89.08%) LECs were stained with tdTomato positive (**Figure 1E, Figure S3C-D**), suggesting that recipient cells were the dominant origin of LECs in cardiac allografts.

We next examined the possible cellular origins of LECs. Bone marrow-derived MSCs have been reported to contribute to cardiac lymphangiogenesis 12. Subsequently, we sought to determine whether there might be a bone marrow source of LECs.* BALB/c* donor hearts were transplanted into *C57BL/6J* recipient mice, which bone marrow was replaced with tdTomato-labeled bone marrow before the operation. Surprisingly, no tdTomato^+^LYVE1^+^ lymphatic vessels were found in transplant hearts (**Figure S3E-G**), excluding the bone marrow as a source of LECs.

To further confirm the potential cellular source of LECs might arise from LYVE1^+^ cells, we then conducted an inducible genetic lineage tracing system for LYVE1^+^ cells by crossing *Lyve1-CreER^T2^* with *R26-tdTomato* mouse lines. The *Lyve1-CreER^T2^;R26-tdTomato* mice were treated with tamoxifen to induce tdTomato labeling of LYVE1^+^ cells, and tissue was collected for labeling efficacy analysis 1 week later. Co-expression of tdTomato and LYVE1 was observed in the aorta, heart, kidney and liver (**Figure S4A**), indicating that the tdTomato+ cells truly represented LYVE1^+^ cells.

We first treated* Lyve1-CreER^T2^;R26-tdTomato* mice as donors (**Figure 1F**) and found that donor-derived LYVE1^+^ cells only labeled a minor proportion (**Figure 1G**) of LECs in cardiac allografts. The whole mount staining of cardiac grafts was employed to clearly visualize lymphatic vessels in the transplanted heart (**Figure S4B**). This was further supported by the whole mount staining of decreased tdTomato^+^ vessels in the cardiac allografts which donor hearts were from *Lyve1-CreER^T2^;R26-tdTomato* mice (**Figure 1H, Supplemental video 1,2**). These results demonstrated that the lymphatic identity of the tdTomato-labeled cardiac vessels gradually disappeared after surgery. Conversely, recipient-derived LYVE1^+^ cells labeled extensive lymphatic vessels (**Figure 1E, I and J**), revealing that the recipient LYVE1^+^ cells are the main contributors of LECs in cardiac allografts. And increased tdTomato^+^ vessels were displayed by the whole mount staining of the allograft hearts after *BALB/c* donor hearts were transplanted into *Lyve1-CreER^T2^;R26R-tdTomato* recipients (**Figure 1K, Supplemental video 3,4**). Thus, these data indicated that lymphatic vessels of cardiac allograft underwent reconstruction after transplantation, and recipient LYVE1^+^ cells were the major cellular origin for LECs (**Figure 1L**).

### Cellular Interaction between Fibroblasts and LECs

**Figure 2:**
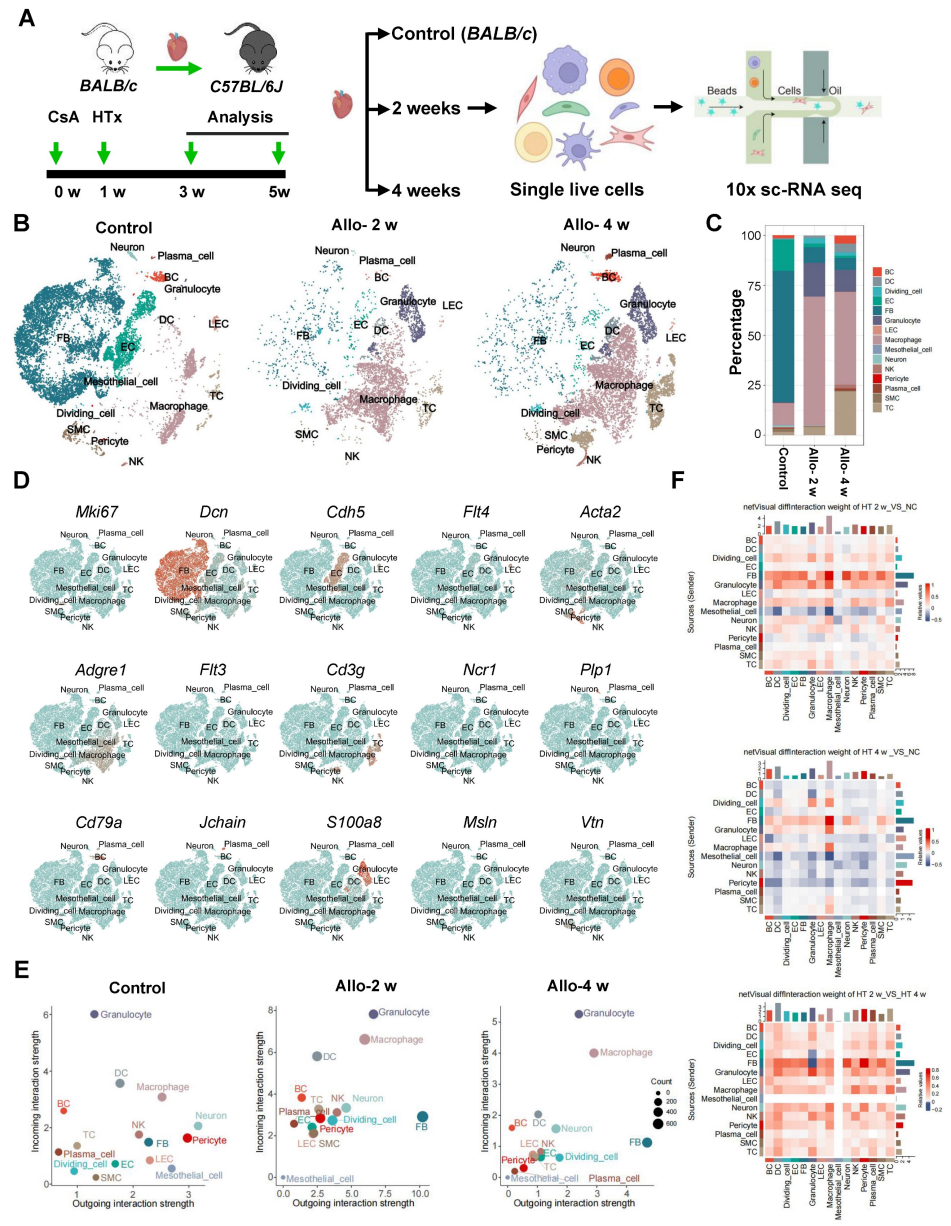
** scRNA-seq Reveals Cellular Interaction between Fibroblasts and LECs. A**, Schematic of experimental strategy and scRNA-seq strategy for heart samples. **B**, t-SNE (t-distributed stochastic neighbor embedding) plot showing the unsupervised clustering of cells isolated from normal *BALB/c* mouse heart (control) and allograft hearts of indicated time points (2 and 4 weeks). **C**, Bar chart showing the proportion of major cell types across samples. **D**, Feature plot showing the expression of marker genes. **E**, Dot plot showing 2D distribution of cell-cell communication network across different groups. **F**, The differential strength of signaling communication compared in different groups. Allo: allograft; CsA: cyclosporine A; HTx: heart transplant; LECs: lymphatic endothelial cells.

To unravel the potential factors would trigger the lymphangiogenesis, we used single-cell RNA sequencing to investigate the cellular and transcriptional landscape of allograft heart after transplantation. *BALB/c* donor hearts were transplanted into *C57BL/6J* recipients, single live nucleated cells isolated from transplanted hearts were sorted by flow cytometry with or without CD45 enrichment (**Figure S5A**) separately at baseline (normal *BALB/c* heart), 2 and 4 weeks after transplantation for single cell RNA sequencing (scRNA-seq; **Figure 2A**). Cells with low quality (<= 400 genes/cell & >= 6000 genes/cell & > 10% mitochondrial transcripts/cell & > 5% hemoglobin related gene transcript presence/cell) were filtered, and a total of 48,243 cells were obtained (Control, 16,726; Allo-2w, 19,623; Allo-4w, 11,894) (**Figure S5B**). Unsupervised clustering with t-distributed stochastic neighbor embedding analysis revealed 15 cell clusters (**Figure 2B-C**), which were further attributed to putative biological identities based on signature of differential gene expression and Gene Ontology enrichment analysis (**Figure S5C**). The cell clusters comprised B cells (BCs, *Cd79a*, *Cd19*), dendritic cells (DCs;* Flt3*), dividing cells (*Stmn1*, *Mki67*), endothelial cells (ECs; *Cdh5*, *Aqp1*), fibroblasts (FBs; *Dcn*, *Pdgfra*), Granulocytes (*S100a8*,* Retnlg*), lymphatic endothelial cells (LECs; *Flt4*, *Lyve1*), macrophages (MFs; *Cd68*, *Adgre1*), mesothelial cells (*Msln*,* Upk3b*), neurons (*Plp1*, *Prnp*), natural killer cells (NK cells;* Ncr1*, *Gzma*), pericytes (*Vtn*,* Pdgfrb*), plasma cells (*Jchain*,* Igkc*), smooth muscle cells (SMCs; *Acta2*, *Myl9*), and T cells (TCs, *Cd3g*, *Cd3d*) (**Figure 2D**).

Then we compared cell state between normal control hearts and allograft hearts, cell-cell communication network revealed that unlike in control hearts, LECs and many other cells in allograft hearts displayed a more active state which was evident with higher incoming/outgoing interaction strength. Of note, it seems that LECs are prone to receive signals rather than to send signals based on higher incoming interaction strength, while fibroblasts appeared as dominant signal sending cells after heart transplant (**Figure 2E**). Meanwhile, by comparing the strength of signal interaction among the three groups (control, allo-2w, allo-4w), we found that both incoming and outgoing interaction strength in allo-2w group were stronger than the other two groups (**Figure 2E**). These results revealed there might be a strong relationship between LECs and fibroblasts at 2 weeks after transplantation. Subsequently, we analyzed cell-cell interaction to confirm the possibility. Cell chat analysis showed strong communication between LECs and fibroblasts, and revealed that these cells were the major regulating-cells in allograft hearts (**Figure 2F**). LECs were also found to be mainly regulated by fibroblasts (**Figure 2F**), coincident with its signal-receiving character analyzed above. Collectively, these data suggested the interaction between LECs and fibroblasts might be responsible for the cardiac lymphangiogenesis after surgery.

### Function Shifts of LECs in Allograft Heart

**Figure 3:**
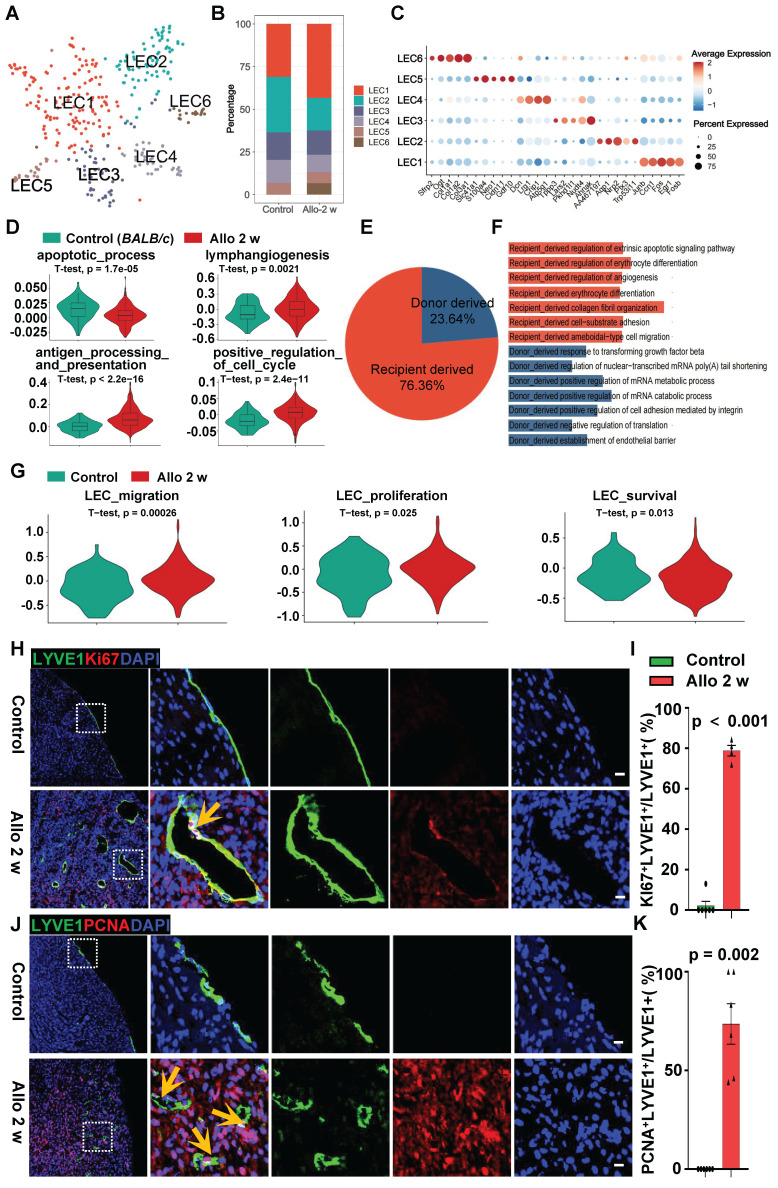
** Function Shifts of LECs in Allograft Heart. A**, t-SNE plot of lymphatic endothelial cell (LEC) populations of control group (*BALB/c*) and allograft-2 w group. **B**, Bar plot showing the percentage of cell composition in two groups.** C**, Dot plot showing average scaled expression levels (color-scaled, column-wise average expression) of top differentially expressed genes (DEGs; columns) across the LEC subpopulations (rows), cells with a value > 0 represent cells with expression above the population mean. Dot size reflects the percentage of cells expressing the selected gene in each population. **D**, Violin plot showing expression of selected gene sets. **E**, Pie plot showing the percentage of LECs from different origins in allograft. **F**, Bar plot showing gene ontology (GO) terms enriched in sub-populations of allograft LECs. **G**, Violin plot showing expression of genes of selected pathways in LECs from two groups (gene.migration: c (*'Flt4','Shc1','Grb2','Pik4r1','Akt1'*); gene.proliferation: c (*'Flt4', 'Shc1', 'Grb2', 'Mapk1'*) gene.survival: c (*'Nrp2', 'Mapk14', 'Cdk6', 'Mapk8', 'Mapk9'*)). **H**, **J**, Immunostaining of LEC proliferation in two groups. The images within the dashed box are displayed on the right. The yellow arrow indicates proliferating LECs. **I, K**, Quantification of Ki67^+^ LECs (**I**) and PCNA^+^ LECs (**K**) in two groups. Number of mice in group (Ki67): n = 6 (Control), n = 4 (Allo-2 w); Number of mice in group (PCNA): n = 6; Mann-Whitney test. Scale bars, 25 µm (H,J). Allo: allograft; LEC: lymphatic endothelial cells.

To investigate the features of LECs after surgery, we performed focused clustering analysis of LECs at a higher resolution, and further separated them into 6 clusters upon differential gene expression (**Figure 3A-C, Figure S6A**). Then the biological processes enriched in each sub-cluster were revealed by GO analysis (**Figure S6B**). In contrast to the situation in normal heart, vasculature development regulation and lymphangiogenesis-related processes such as lymph vessel morphogenesis and lymph vessel development were present in LEC1 and LEC5 subpopulation respectively after heart transplant (**Figure S6B**), suggesting allograft LECs are more intended to form new lymphatic vessels. Cluster LEC2 in allograft heart was proposed to engaged in immune rejection due to its ability of antigen processing and response to IFN-γ (**Figure S6B**). Furthermore, LEC4 and LEC6 sub-clusters in allograft heart were more prone to engage in the processes include ECM organization, extracellular structure organization and collagen fibrin organization (**Figure S6B**). Interestingly, LEC6 subpopulation was uniquely observed in allograft hearts (**Figure 3B, Figure S6B**). Next we analyzed the gene sets that might affect the generation of new lymphatic vessels in two groups, the LECs in allograft heart group displayed higher expression of genes related to lymphangiogenesis, antigen processing and presentation, positive regulation of cell cycle (**Figure 3D, Figure S6C**). Meanwhile, the apoptotic process associated genes expression decreased in allograft LECs (**Figure 3D**).

As we mentioned above, recipient cells are the major contributors for cardiac LECs after heart transplantation. This was also supported by the Y chromosome analyses of LECs in allograft hearts. The results showed 76.36% LECs are Y chromosome positive (**Figure 3E**), further indicating cardiac LECs are mainly derived from recipients. Subsequently, we performed GO analysis to delineate the differences of functional roles between donor and recipient LECs in cardiac allografts (**Figure 3F**). Donor derived LECs displayed a steady phenotype because the processes include “positive regulation of cell adhesion”, “establishment of endothelial barrier” were identified, whereas the processes such as “regulation of angiogenesis” “collagen fibril organization” were enriched in recipient derived LECs, revealing these LECs were more active and could have higher potential to form new lymphatic vessels after transplantation.

Additionally, gene sets of LEC migration (*Flt4, Shc1, Grb2, Pik4r1, Akt1*), proliferation (*Flt4, Shc1, Grb2, Mapk1*) and survival (*Nrp2, Mapk14, Cdk6, Mapk8, Mapk9*) were compared between two groups, results showed enhanced migration, proliferation and survival genes expression in transplant groups (**Figure 3G**). This was validated by strong inmmuno-staining of p-AKT and p-ERK in LECs of transplant groups (**Figure S6D**). In order to confirm the features of LECs in cardiac allograft hearts, co-immunostaining of Ki67 or PCNA with LYVE1 was applied to validate the cellular activity of LECs. Compared with the control, allograft LECs revealed higher proliferation potential which was evident by the positive staining of Ki67 and LYVE1, or PCNA and LYVE1 (**Figure 3H-K, Figure S6E**). Collectively, these data demonstrate that with the change of the environment in transplanted heart, LECs acquired the property of being more likely to form lymphatic vessels.

### Fibroblasts Display Activated Phenotype in Allograft Heart

**Figure 4:**
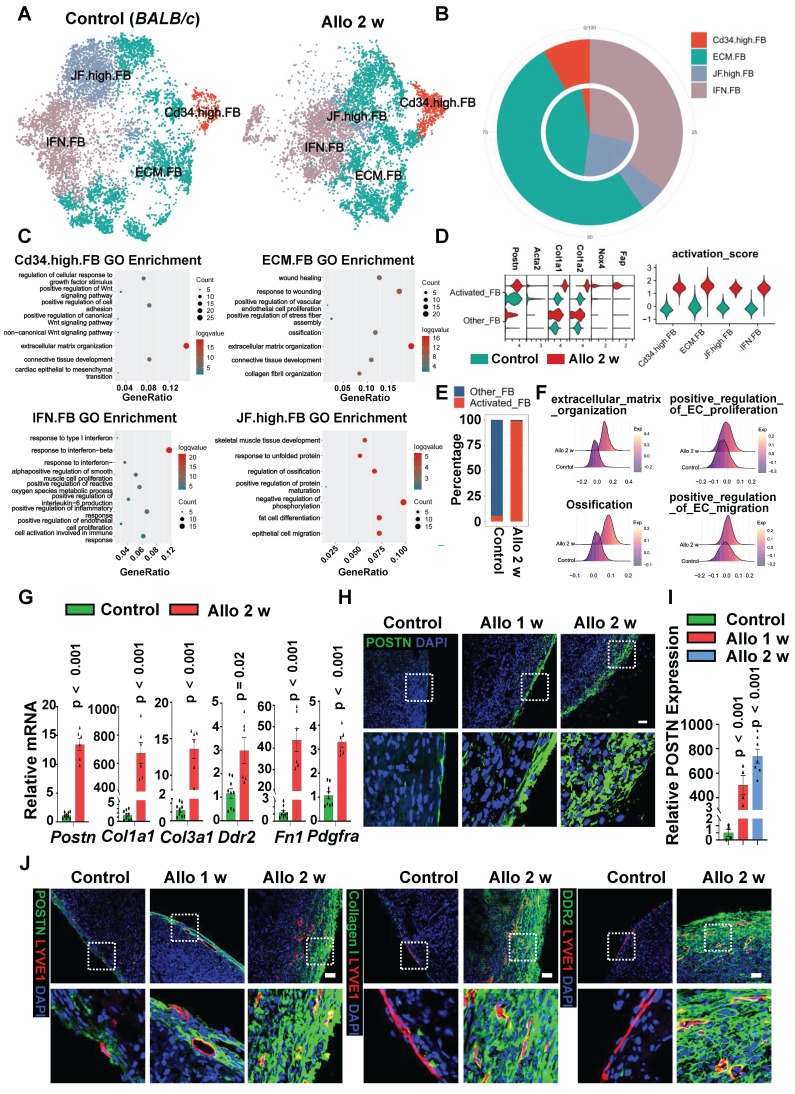
** Fibroblasts Display Activated Phenotype in Allograft Heart. A**, t-SNE plot of fibroblast populations of control group (*BALB/c*) and allograft-2 w group. Cd34.high.FB, high expression of FB; ECM.FB, enriched genes and functions related to ECM organization; JF.high.FB, high expression of *Jun* and *Fos*; IFN.FB, high expression of genes related to interferon. **B**, Pie plot showing the percentage of cell composition in two groups (inside: control; outside: allo-2 w). **C**, Dot plot shows selected GO terms enriched in fibroblast sub-clusters. **D**, Violin plot showing the combination expression level (right) of selected activated fibroblast genes (left) in sub-population from two groups. **E**, Bar plot showing the percentage of activated fibroblasts and other fibroblasts in two datasets. The activated fibroblasts were identified according to combination expression level of genes in Figure 4d. **F**, Ridgeline chart showing the expression score of fibrosis-related score in fibroblasts from two groups. **G**, mRNA quantification of activated fibroblast-associated genes in the hearts from two groups, mRNA expression in the control group as the criterion (1.0). Number of mice in each group: n = 9 (Control), n = 6 (Allo-2 w); unpaired t-test. *Postn* (Periostin), *Col1a1* (Collagen, type I, alpha 1), *Col3a1* (Collagen, type III, alpha 1), *Ddr2* (Discoidin domain-containing receptor 2), *Fn1* (Fibronectin), *Pdgfra* (Platelet-derived growth factor receptorα). **H**, **I**, Immunostaining (**H**) and quantification (**I**) of POSTN expression in control group and allograft group, the area of POSTN expression in the control group as the criterion (1.0). The images within the dashed box are displayed in the second row. Number of mice in each group: n = 4 (Control), n = 4 (Allo-1 w), n = 7 (Allo-2 w); one-way ANOVA test. **J**, Immuno-staining of POSTN and LYVE1, Collagen I and LYVE1, DDR2 and LYVE1 in two groups. The images within the dashed box are displayed in the second row. Scale bars, 100 µm (H,J). Allo: allograft; FB: fibroblast.

To provide further evidence for fibroblasts in regulating LECs in allograft hearts, we compared the properties of fibroblasts between control group (normal *BALB/c* heart) and allo-2w group (allograft hearts after 2 weeks of transplantation). A total of 20,496 fibroblasts were extracted from the data set and 4 fibroblast sub-clusters, including ECM-FB, IFN-FB, JF high-FB and CD34 high-FB were identified based on the differential genes expression (**Figure 4A, Figure S7A-B**).

After heart transplantation, the proportion of CD34 high-FB and ECM-FB subsets were significantly increased (**Figure 4B**). Functional analysis showed that both CD34 high-FB and ECM-FB subsets were mainly enriched in extracellular matrix organization (**Figure 4C**), indicating that the secretion of ECM-related factors by fibroblasts increased after transplantation. As for IFN-FB subset, it is a fibroblast subpopulation associated with enrichment of response to interferon-beta, positive regulation of cytokine production and other processes such as positive regulation of endothelial cell proliferation (**Figure 4C**), in which the proportion also increased in cardiac allografts compared with the control group (**Figure 4B**). The results implied that cardiac fibroblasts displayed a secreted phenotype and might be involved in inflammatory responses and vasculature remodeling after transplantation.

Activated fibroblasts in disease have traditionally been referred to as myofibroblasts 22-24, in part because they express contractile genes such as *Acta2*, which encodes smooth muscle αactin (αSMA).

Here we identified a population of fibroblasts expressed high ECM, proteoglycans and proteases associated genes (**Figure 4D, Figure S7C**), but very low level of gene *Acta2* (**Figure 4D**). In some injured tissues, these non-contractile but activated fibroblasts (not myofibroblasts) are considered to be even more adaptive at ECM production and driving fibrotic response 25. According to the expression score of markers genes of activated fibroblasts reported in previous studies (*Fap*, *Nox4*, *Col1a1*, *Col1a2*, and *Postn*), we classified these four fibroblast subsets into two subpopulations: activated fibroblasts (not myofibroblasts) and other fibroblasts (**Figure 4D**). By comparing with the fibroblasts in control group, most fibroblasts were activated after cardiac transplantation (**Figure 4E**), and fibroblasts in cardiac allografts were more likely to engage in extracellular matrix organization, ossification and positive regulation of EC proliferation and migration (**Figure 4F**). Interestingly, the analysis of the origin of fibroblasts in cardiac allografts revealed that a large number of the activated fibroblasts were derived from recipients (**Figure S7D**), indicating that recipient-derived fibroblasts may play an important role in the hearts after transplantation.

In addition, fresh tissues from control hearts and allograft hearts were isolated, ECM-related and fibroblast-associated gene markers (*Postn*, *Col1a1*,*Col3a1*, *Ddr2*, *Fn1*, and *Pdgfra*) were analyzed by q-PCR (**Figure 4G**) and immunostaining (**Figure 4H-I, Figure S7E-F**), displaying much higher expression post-transplantation. We next examined the proliferation state of fibroblasts, heart sections immunostaning against PCNA and POSTN/PDGFR-α suggested that the proliferation ability of fibroblasts in the transplanted heart was greatly enhanced compared with control hearts (**Figure S7G-J**). The results further confirmed that fibroblasts became activated after transplantation. In theory, it could be an effective method for these activated fibroblasts to participate in lymphatic vessel formation by maintaining anatomical proximity to the lymphatic vessels. Immunofluorescence staining showed that lymphatic vessels were indeed distributed among the activated fibroblasts in the transplanted heart (**Figure 4J**), therefore, we speculate that lymphangiogenesis in the cardiac allograft is probably regulated by activated fibroblasts.

### Ablation of Activated Fibroblasts Suppresses Lymphangiogenesis and Leads to Graft Failure

**Figure 5:**
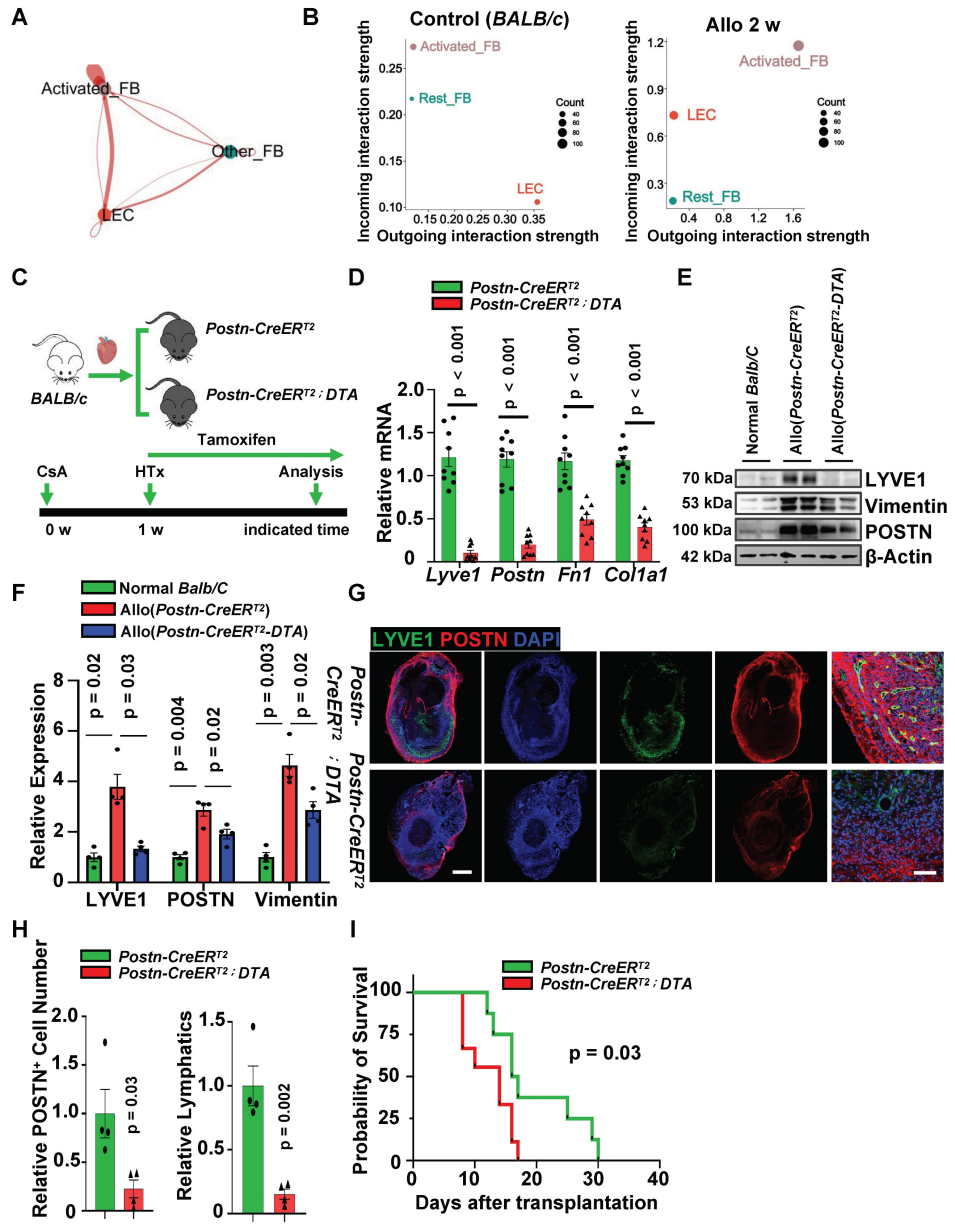
** Ablation of Activated Fibroblasts Alleviates Lymphatic Vessel Formation and Leads to Graft Failure. A**, Circle plot showing the differences in communication strength between cardiac allografts group and normal hearts group. Red infers to increased enrichment in allografts, blue refers to decrease in allografts. **B**, Dot plot showing 2D distribution of cell-cell communication (activated FB - LEC, other FB - LEC) network across two groups. **C**, Sketch of the experimental design for*Postn*^+^ cells depletion using *Postn*-*CreER^T2^*-diphtheria toxin subunit A (DTA) mice. *Postn*-*CreER^T2^* mice were used as control mice (Ctr). **D**, qPCR quantification of *Lyve1*, *Postn*, *Fn1* and *Col1a1* in the cardiac allografts from *Postn-CreER^T2^* (Ctr group) and *Postn-CreER^T2^-DTA* (*Postn-DTA* group) mice treated with tamoxifen after 2 weeks of transplantation, mRNA expression in the control group as the criterion (1.0). Number of mice in each group: n = 9; unpaired t-test. **E**,**F**, Western blotting assay (**E**) and quantification (**F**) of LYVE1, POSTN and Vimentin in the hearts of normal *BALB/c* mice, allograft hearts from *Postn-CreER^T2^* and *Postn-CreER^T2^-DTA* mice treated with tamoxifen after 2 weeks of transplantation, the protein expression in the normal *BALB/c* group as the criterion (1.0). Number of mice in each group: n = 4; one-way ANOVA test. **G**, Immunostaining of POSTN and LYVE1 of cardiac allografts in two groups after 2 weeks of transplantation. **H**, Quantification of POSTN positive cells and lymphatic vessels in two groups, POSTN^+^ cell number in the control group (*Postn-CreER^T2^*) as the criterion (1.0). Number of mice in each group: n = 4; unpaired t-test. **I**, Survival time of cardiac allografts in two groups. Number of mice in each group: n = 8 (*Postn-CreER^T2^* group), n = 9 (*Postn-CreER^T2^-DTA* group); Gehan-Breslow-Wilcoxon test. Scale bars, 1 mm (G-left), 100 µm (G-right). CsA: cyclosporine A; FB: fibroblast; HTx: heart transplant.

To demonstrate that the generation of lymphatic vessels in transplanted hearts is primarily regulated by activated fibroblasts, we compared the strength of cellular communication in other fibroblasts, activated fibroblasts and LECs both in control group and transplantation group. Thicker red lines indicate higher strength of interaction between cells in transplanted hearts. Analysis showed activated fibroblasts were the dominant regulators for LECs after heart transplantation (**Figure 5A**). We also confirmed that the signals received by LECs after heart transplantation mainly come from activated fibroblasts based on the analysis of cellular outgoing/incoming interaction strength (**Figure 5B**).

To further validate the regulation of activated fibroblasts for lymphangiogenesis in vivo, we next sought to examine lymphangiogenesis by deleting activated fibroblasts after transplantation. Our data from scRNA-seq analysis implied that a large population of activated fibroblasts in cardiac allografts were recipient derived (**Figure S7D**), therefore we crossed the *Postn-CreER^T2^*and diphtheria toxin subunit A (*DTA*) mouse lines to allow selective ablation of the POSTN^+^ cell population at indicated time points. Cre recombinase activation by tamoxifen administration leads to the specific expression and activation of the *DTA* in recipient POSTN^+^ cells, resulting in termination of protein synthesis and apoptotic cell death. We treated the *Postn-CreER^T2^-DTA*mice with tamoxifen after heart transplantation,* Postn-CreER^T2^* mice were used as control (**Figure 5C**). Whole hearts after transplantation were acquired and POSTN expression was analyzed, great POSTN reduction was detected in allograft hearts after tamoxifen induction (**Figure 5D-F**), immunostanining of POSTN also showed a decrease of POSTN^+^ cells in allograft hearts (**Figure 5G, H**), and the formation of lymphatic vessels in the transplanted heart was potently inhibited, further proving that lymphangiogenesis is activated fibroblasts dependent after transplantation (**Figure 5G-H**).

Surprisingly, the removal of activated fibroblasts in transplanted hearts not only suppressed the formation of lymphatic vessels but also led to an earlier onset of graft failure (**Figure 5I**). To verify whether this phenomenon was caused by impaired lymphatic vessel network, we treated transplanted mice with VEGFR3 inhibitor (SAR131675) to hinder lymphatic vessel growth (**Figure S8A**). We found that compared to the control group, mice injected with SAR131675 exhibited impaired cardiac lymphatic vessel formation (**Figure S8B-C**) and significantly decreased graft survival time (**Figure S8D**). Further analysis of the transplanted hearts after 12 days revealed that, compared to the control group, the SAR131675 group exhibited a significant increase in inflammatory cell infiltration and upregulation of inflammatory cytokines expression (**Figure S8E-G**). These results indicate that recipient-derived lymphatic vessels could be beneficial to the heart, at least in the early stages of transplantation, possibly by clearing inflammatory cells and inflammatory factors from the cardiac allografts. Additionally, early activation of fibroblasts post-transplantation promotes lymphangiogenesis, which is necessary for maintaining graft function.

### Activated Fibroblasts Secrete Multiple Cytokines to Interact with LECs in Allograft Hearts

**Figure 6:**
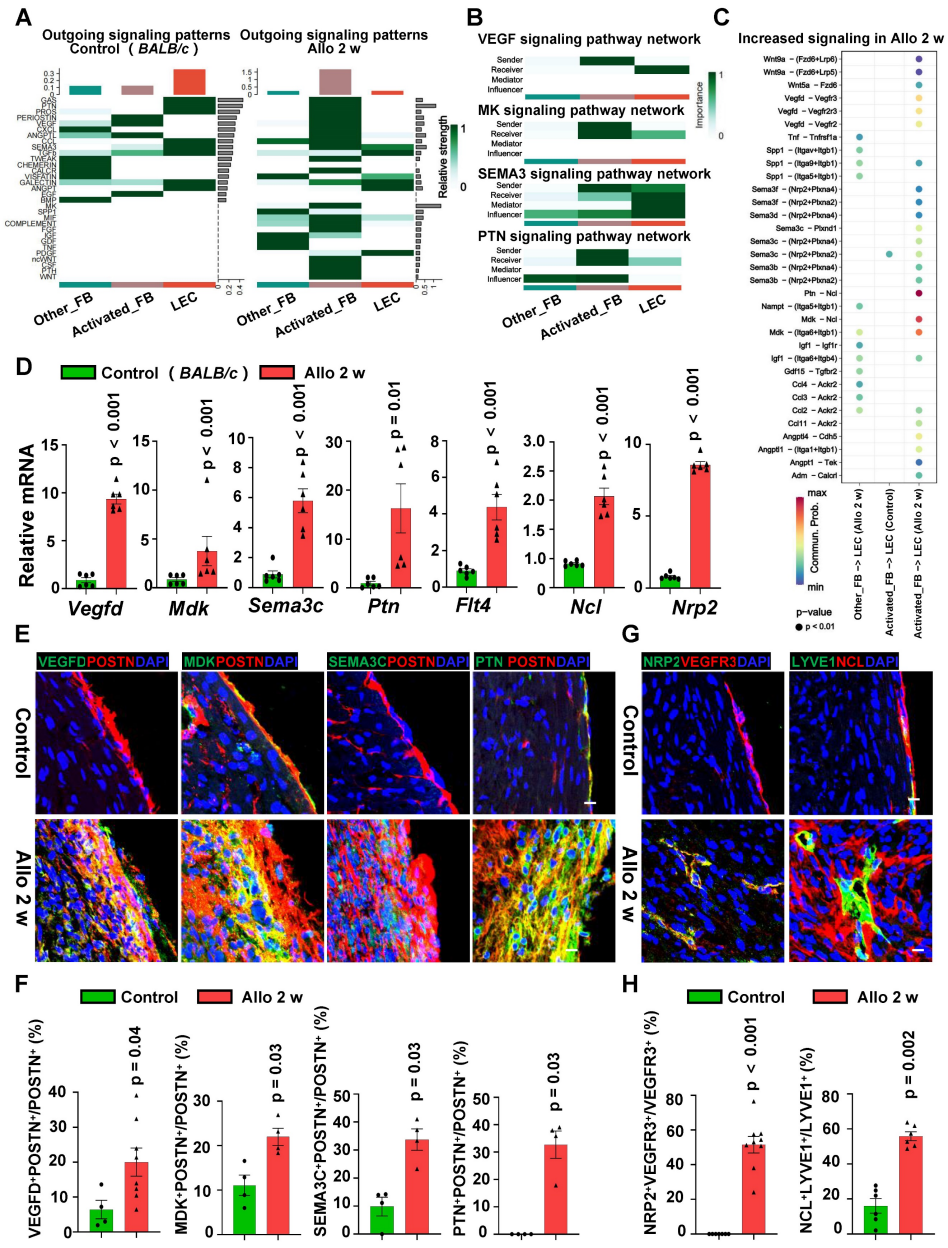
** Activated Fibroblasts Secrete Multiple Factors to Interact with LECs in Allograft Heart. A**, Heatmap showing the signaling patterns enriched in fibroblasts and LECs of control group and cardiac allograft group. **B**, Heatmap showing the signaling patterns enriched in two fibroblast sub-populations (activated FB and other FB) and LECs. **C**, Increased ligand-receptor pairs enriched between fibroblasts and LECs after 2 weeks of transplantation. **D**, mRNA expression of activated fibroblast secreted ligand genes (*Vegfd*, *Mdk*, *Sema3c*, *Ptn*) and LEC expressed receptor genes (*Flt4*, *Ncl*, *Nrp2*) in the cardiac allografts from two groups, mRNA expression in the control group as the criterion (1.0). Number of mice in each group: n = 6; unpaired t-test. *Vegfd* (vascular endothelial growth factor D), MDK (midkine), *Sema3c* (semaphorin-3C), *Ptn* (pleiotrophin), Flt4 (Fms-related tyrosine kinase 4), *Ncl* (nucleolin), *Nrp2* (neuropilin 2). **E**, **F**, Immunostaining (**E**) and quantification (**F**) of the ligands in activated fibroblasts of the cardiac allografts from two groups. Number of mice in VEGFD expression group: n = 4 (Control), n = 8 (Allo 2 w); Number of mice in other ligands expression group: n = 4; unpaired t-test. **G**, **H**, Immunostaining (**G**) and quantification (**H**) of the receptors in LECs of the cardiac allografts from two groups. Number of mice in NRP2 group: n = 6 (Control), n = 9 (Allo 2 w); Number of mice in NCL group: n = 6; unpaired t-test. Scale bars, 25 µm (E, G). Allo: allograft.

Due to the predominant distribution of newly formed lymphatic vessels around activated fibroblasts, we hypothesize that activated fibroblasts may promote lymphatic vessel neogenesis through paracrine signaling. To validate this hypothesis, we conducted scRNA-seq analysis of transplanted hearts to investigate the molecular basis of the crosstalk between fibroblasts and LECs. Pathway enrichment revealed that cellular signals are more active in the cardiac allografts than in the control heart, of note, the genes related to lymphangiogenesis such as *Vegf, Sema3, Mk* and *Ptn* were mainly present in allograft hearts (**Figure S9A**). We further analyzed the outgoing signaling patterns of fibroblasts and LECs in the hearts before and after surgery. Compared to fibroblasts of control hearts, fibroblasts especially the activated fibroblasts in the allografts displayed higher potential to send signals (**Figure 6A**). Analysis of lymphangiogenesis associated signaling pathway network also approved that activated fibroblasts were the signal senders, which secreted VEGF/MK/SEMA3/PTN family proteins to the receiver LECs (**Figure 6B**). Based on above signal expression patterns, signature ligand-receptor pairs were subsequently analyzed and four major signal pairs include *Vegfd/Vegfr3*, *Mdk/Ncl, Sema3c/Nrp2* and* Ptn/Ncl* were identified in the crosstalk between activated fibroblasts and LECs after heart transplantation (**Figure 6C, Figure S9B**).

In order to verify the results from scRNA-seq, we harvested the fresh heart tissue and evaluated the mRNA expression of the ligand genes (*Vegfd, Mdk, Sema3c* and* Ptn*) and receptor genes (*Flt4, Ncl* and* Nrp2*). Gene expression were significantly up-regulated in allograft hearts comparing with the expression in control hearts (**Figure 6D**). To determine whether these highly expressed ligand proteins are produced by activated fibroblasts, two groups of immunostaining of ligands (VEGFD, MDK, SEMA3C and PTN) in fibroblasts (**Figure 6E-F, Figure S9C**) were applied. It appeared that cardiac allografts had abundant co-expression of aforementioned ligand proteins and POSTN (**Figure 6E-F, Figure S9D**). Here we used POSTN to represent activated fibroblasts. Although POSTN is not exclusive to activated fibroblasts during cardiac development and in other tissues 26, however, in adult stressed cardiac ventricles, POSTN is uniquely expressed by activated fibroblasts 24, 27-29. Meanwhile, receptor expression (VEGFR3, NCL and NRP2) in LECs were also up-regulated after transplantation (**Figure 6G-H, Figure S9E**). These results illustrated that activated fibroblasts may secrete multiple cytokines, rather than a single factor, which trigger the lymphangiogenesis after the heart transplantation in a synergistic mechanism.

### Fibroblasts Facilitate LECs to Form Lymphatic Vessels Mainly via VEGFD/VEGFR3, MDK/NCL and SEMA3C/NRP2 Pathways

**Figure 7:**
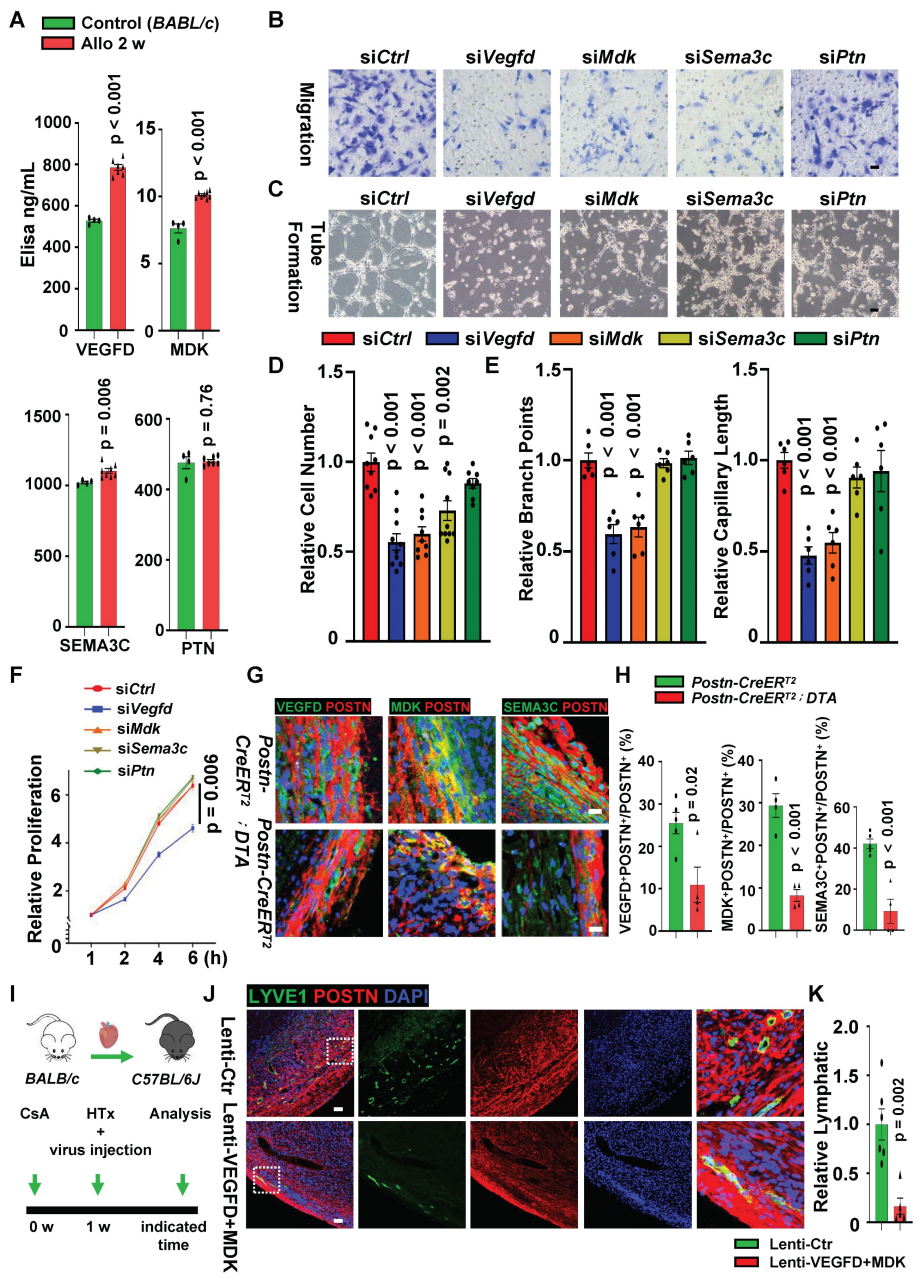
** Fibroblasts Facilitate Lymphatic Vessel Formation Mainly via VEGFD/VEGFR3, MDK/NCL and SEMA3C/NRP2 Pathways. A**, Elisa assay of ligands (VEGFD, MDK and SEMA3C) in control fibroblasts and allograft fibroblasts. Number of mice in each group: n = 4 (Control), n = 7 (Allo 2 w); unpaired t-test. **B**, **D**, Transwell assay (**B**) and quantification (**D**) of LECs co-cultured with allograft fibroblasts (treated with negative control siRNA or targeted ligand genes siRNA), cell number in the control group as the criterion (1.0). Number in each group: n = 9 (siCtrl), n = 10 (siVegfd), n = 9 (siMdk), n = 10 (siSema3c), n = 8 (siPtn), one-way ANOVA test. **C**, **E**, Tube formation assay (**C**) and quantification (**E**) of LECs cultured with allograft fibroblasts conditioned medium (treated with negative control siRNA or targeted ligand genes siRNA), the branch points and the capillary length in the control group as the criterion (1.0). n = 6, one-way ANOVA test. **F**, CCK8 assay of LECs cultured with allograft fibroblasts conditioned medium. n = 6, one-way ANOVA test. **G**, **H**, Immunostaining (**G**) and quantification (**H**) of secreted fibroblast ligands in the cardiac allografts from the POSTN^+^ cell depletion group and the control group. Number of mice in each group: n = 5 (*Postn-CreER^T2^* group), n = 4 (*Postn-CreER^T2^-DTA* group); unpaired t-test. **I**, Sketch of the experimental design for knockdown of VEGFD and MDK expression in POSTN^+^ cells in the cardiac allografts. **J**, **K**, Immunostaining (**J**) and quantification (**K**) of activated fibroblasts (POSTN^+^) and lymphatic vessels (LYVE1^+^) incardiac allografts from Lenti-Ctr group (control) and Lenti-VEGFD+MDK group. The images within the dashed box are displayed on the right. The lymphatic vessel density of the control group as the criterion (1.0). Number of mice in each group: n = 6 (Lenti-Ctr group), n = 5 (Lenti-VEGFD+MDK group); Gehan-Breslow-Wilcoxon test. Scale bars, 50µm (B, J), 200 µm (C), 25 µm (G), and 100 μm (J). Allo: allograft; CsA: cyclosporine A; HTx: heart transplant.

Since lymphangiogenesis mainly depends on the proliferation, migration and tube formation of LECs, it is essential to investigate whether fibroblasts facilitate lymphangiogenesis by regulating these functions of LECs. To confirm that in vitro cultured fibroblasts can directly modulate the function of LECs, we first isolated and verified the features of fibroblasts from allograft hearts 2 weeks after transplantation. These fibroblasts displayed an activated phenotype, which was evidenced by co-immunostaining of POSTN and vimentin or other ECM proteins like fibronectin, fibroblasts from normal hearts were used as control (**Figure S10A**). This was further validated by Western blotting with higher protein expression of POSTN and fibronectin in allograft fibroblasts than in control heart fibroblasts (**Figure S10B**). In order to figure out whether these allograft fibroblasts in vitro culture have more potential to promote the formation of lymphatic vessels by LECs than control fibroblasts, we compared the expression of VEGFD, MDK SEMA3C and PTN in the two kinds of fibroblasts. Elisa analysis of these secreted ligands in culture medium supernatant showed increased concentration of VEGFD, MDK and SEMA3C in allograft fibroblasts compared with control fibroblasts (**Figure 7A**), but no differences of PTN were observed between two fibroblast groups (**Figure 7A**). Immunostaining and Western blotting assay also revealed that the expression of VEGFD, MDK and SEMA3C in cultured allograft fibroblasts were significantly higher than those in control fibroblasts (**Figure S10C-E**). These data suggest that the transplanted cardiac fibroblasts cultured in vitro retained features of activated fibroblasts as in vivo, which is beneficial for subsequent studies.

Subsequently, we analyzed the migration, proliferation and tube formation of LECs by co-culturing mouse LECs with fibroblasts or fibroblasts conditioned medium. After 24 hours of co-culture with fibroblasts, a stronger migration ability of LECs was observed in allograft fibroblasts group, compared to control fibroblasts group (**Figure S10F-G**). In addition, conditioned medium harvested from fibroblasts was applied to culture LECs, a significant improvement in lymphatic tube formation (**Figure S10H-I**) and LEC proliferation (**Figure S10J**) were recorded in the allograft fibroblasts conditioned medium treated group. These in vitro results further confirmed that fibroblasts from transplanted hearts could promote lymphatic vessel formation by regulating the proliferation, migration and tube formation of LECs.

scRNA-seq analysis suggested that the crosstalk between allograft fibroblasts and LECs depends on their ligand-receptor pairs, we first knocked down the ligand genes (*Vegfd, Mdk, Sema3c* and* Ptn*) of allograft fibroblasts (**Figure S11A**) to examine whether LEC functions were dependent on these fibroblast ligands. The results showed that LECs migration was significantly attenuated in knockdown groups (si*Vegfd, siMdk* and si*Sema3c*) compared with the control group (si*ctrl*) (**Figure 7B and D**) after 24 hours of co-culture with allograft fibroblasts. Conditioned medium harvested from genes-knockdown fibroblasts was applied to culture LECs, tube formation of LECs was impaired by the down-regulating expression of VEGFD and MDK in fibroblasts (**Figure 7C and E**), and LECs proliferation seems to be mainly regulated by VEGFD (**Figure 7F**). Subsequently, the receptor genes (*Flt4, Nrp2, Ncl*) expression on the LECs was respectively inhibited (**Figure S11B-C**) to evaluate whether regulatory effect of allograft fibroblasts on LEC functions still exists. We observed that LEC migration was greatly decreased (**Figure S11D-E**), tube formation was found impaired (**Figure S11F-G**) and the proliferation of LECs was attenuated (**Figure S11H**) after receptors knockdown. Taken together, these data suggested that allograft fibroblasts could promote LEC proliferation, migration and tube formation via multiple ligand-receptor pairs, of which VEGFD/VEGFR3, MDK/NCL and SEMA3C/NRP2 pathways would be the dominant contributors for facilitating lymphatic vessel formation in cardiac allografts.

Given that lymphangiogenesis was limited by the loss of activated fibroblasts in allograft hearts, whether the reduction in lymphatic vessel formation is associated with the decreased expression of activated fibroblast-secreting ligand genes (*Vegfd, Mdk and Sema3c*) is still to be addressed. We next examined these ligands expression between activated fibroblasts ablation group and control group. mRNA quantification and histological examination in allograft hearts were performed between two groups and revealed that the expression of VEGFD, MDK and SEMA3C was dramatically reduced in the *Postn*^+^ cells ablation group after heart transplantation (**Figure S12A, Figure 7G-H**), suggesting that decreased expression of activated fibroblast derived ligands could be accounting for the impairment of lymphangiogenesis after heart transplantation. Furthermore, lentiviral vectors which target POSTN-expressed cells and could silence *Vegfd* or *Mdk* gene in POSTN^+^ cells were constructed, these viruses were injected in the donor mouse hearts on the transplanting day and allograft hearts were harvested when palpitation stopped (**Figure 7I**). Comparing with control group (donor hearts were administered with the control lentiviral vectors), both VEGFD and MDK expression were successfully knocked down in the transplanted hearts which were treated with POSTN^+^ cells targeting lenti-VEGFD and lenti-MDK vectors (**Figure S12B-D**). More importantly, lymphatic vessel formation was significantly inhibited and the survival time of the cardiac allografts was declined in the the knockdown group (**Figure 7J-K, Figure S12E**). Taken together, these results demonstrated that lymphangiogenesis in transplanted hearts is regulated by activated fibroblasts in a synergistic effect of multiple ligands secreted by activated fibroblasts after transplantation.

To confirm whether there is a correlation between activated fibroblasts and lymphatic vessels in heart-transplant patients, we collected biopsy samples from the patients for analysis. The tissue samples harvested from normal heart were used as control. In post-transplant patients, cardiac lymphatics were found relatively increased compared with the controls (**Figure S13A-B**). POSTN expression was also improved in the patient group (**Figure S13C-D**) and the correlation between activated fibroblasts and lymphatic vessels was confirmed by the Pearson test of POSTN and LYVE1 positive area in the transplanted heart (**Figure S13E**). Furthermore, we examined the expression of lymphangiogenic-related glycoprotein VEGFD, which secretion was significantly increased in cardiac biopsy samples after transplantation (**Figure S13F-G**). These findings implicate that results obtained from the mouse model share many similarities to patients with heart transplantation in terms of lymphangiogenesis.

## Discussion

Insight into the origin and development of the cardiac lymphatics has important implications for understanding heart tissue fluid homeostasis, inflammation-related immune responses and other pathological processes in transplanted hearts. Despite intensive efforts to definitively assign the origins of cardiac lymphatics after heart transplantation, the origin of newly formed lymphatic vessels in the transplanted heart remains a topic of ongoing debates. It has been shown that cardiac lymphatics were donor-derived in a mouse intraperitoneal heart transplant model 12, 13. However, the lymphatic vessels detected in lesions of human cardiac allografts are considered to be from the recipient 11. In our study, mouse heterotopic cervical heart transplant model was applied and tdTomato labeled mice were introduced as donors or recipients to explore the origin of cardiac lymphatics.

In contrast to the previous study, we observed that the majority (89.08%) of lymphatic vessels were derived from recipients, and only a minority (8.9%) were of donor origin. In addition, Y chromosome analysis of scRNA-seq of the cardiac allografts, which female *BALB/c* hearts were moved into male *C57BL/6J* mice, indicating that 76% LECs were recipient-derived while 23% LECs were donor-derived. These results suggest that in our mouse model of heart transplantation, the cardiac lymphatic vessels are present in a “lymphatic chimera” in which most of the lymphatic vessels are derived from the recipient. It is worth noting that inhibiting the generation of lymphatic vessels can lead to graft failure in transplanted hearts. This may be due to the establishment of connections between recipient-derived lymphatic vessels of cardiac allograft and the pre-existing lymphatic network in the recipient mouse, allowing for timely clearance of excessive tissue fluid and inflammatory substances in the transplanted heart. Therefore, establishing recipient-derived lymphatic vessels at least in the early stages of transplantation may be beneficial for the transplanted heart.

In mice, lymphatic vessels mainly arise from venous origin and non-venous origin 30-32, and many lymphatic endothelial progenitor cells are considered to contribute in cardiac lymphatics both during development and cardiac remodeling 33, 34. For example, bone marrow derived CD34^+^VEGFR3^+^ progenitor cells are claimed to be able to differentiate into LECs to increase cardiac lymphangiogenesis in the adult rat model after myocardial infarction 34. However, the investigations on the cellular origin of lymphatic vessels in heart transplantation are still at an early stage. The whole-mount staining of tdTomato labeled LYVE1^+^ cells in the transplanted heart showed that donor-derived tdTomato labeled cells decreased, whereas recipient-derived tdTomato labeled cells gradually appeared, demonstrating that recipient LYVE1^+^ cells reconstructed cardiac lymphatics after transplantation.

Previous studies mainly focused on the effect of inflammatory cells (macrophages, T cells, etc.) on lymphangiogenesis after transplantation 12, while the function of other cells in this issue was largely overlooked. Actually, current understanding of the functions of cardiac fibroblasts has moved beyond their roles in heart structure and extracellular matrix generation, but now includes their contributions to paracrine signalling during cardiac remodeling after myocardial ischaemia, hypertension and heart failure 22. A recent study on the function of fibroblasts at different time points after myocardial infarction found that 3 days after myocardial infarction, fibroblasts displayed a proliferative state and promoted angiogenesis through up-regulation of Il4ra signaling, while by 7 days after myocardial infarction, fibroblasts showed an anti-angiogenic homeostatic-like myofibroblast profile and with a step-wise increase in *Acta2* expression 35. This indicates that the function of fibroblasts is different at different stages after myocardial infarction, and that fibroblasts can promote angiogenesis before transforming into myofibroblasts. We therefore speculated that there might exist a population of fibroblasts which could facilitate lymphangiogenesis at some time point after heart transplantation. In this study, we revealed that after 2 weeks of transplantation, cardiac LEC functions were primarily regulated by the activated fibroblasts (not myofibroblasts), which highly specialized in the generation and secretion of fibrillar collagens and matri-cellular proteins but very low expression of contractile genes such as *Acta2*. These activated fibroblasts were proved to have potential to promote LEC proliferation, migration and tube formation in culture system. Interestingly, the majority of activated fibroblasts were found originating from recipients and the ablation of these recipient-derived activated fibroblasts after heart transplantation resulted in a reduced lymphangiogenesis, which also leads to reduced survival time of the transplanted hearts, this emphasizes the critical role of activated fibroblasts in promoting lymphatic vessel formation and maintaining graft viability.

Another intriguing discovery in this study is that activated fibroblasts facilitate lymphangiogenesis based on a multiple signaling pathways such as VEGFD/VEGFR3, MDK/NCL and SEMA3C/NRP2, but not through classical factor VEGFC 36, which was identified previously as a major factor secreted by macrophages and CD4^+^ T cells in the transplanted heart. MDK is generally proposed to mediate tumor metastasis by its angiogenic function, but recently it is revealed a pro-lymphangiogenic role for MDK in cutaneous melanoma 37. Here we found MDK is secreted by activated fibroblasts and confirmed its role in LEC migration and tube formation in the transplanted heart. SEMA3C has been observed to function as a potent inhibitor of angiogenesis and lymphangiogenesis, which is likely mediated by the activation of plexin-D1-dependent signal transduction in endothelial cells and lymphatic endothelial cells, resulting in the inhibition of tumor progression 38. However, in our study, SEMA3C expression was upregulated in activated fibroblasts of cardiac allografts, and the conditioned medium harvested from allograft fibroblasts displayed higher potential to improve LEC migration, while *Sema3c* gene knockdown in allograft fibroblasts greatly inhibited LEC migration, which is a vital step to help LECs to form lymphatic vessels. Thus, it could be likely that activated fibroblasts in transplanted heart secrete different cytokines to affect functions of LEC, and these signals act synergistically to promote cardiac lymphangiogenesis after transplantation. This is also verified by the impaired lymphangiogenesis after co-injection of lentiviral vectors which targeting POSTN+ cells to knockdown VEGFD and MDK in transplanted hearts. Given that cardiac lymphangiogenesis is a complex and dynamic process post-transplantation, it is more reasonable that lymphatic vessel formation is regulated by multiple signals rather than by only one.

A limitation of this study is that we did not further explore the ways by which LYVE1^+^ cells entered the transplanted heart and whether LYVE1^+^ cells might have stem cell properties, because recent studies in our laboratory have shown that circulating-derived endothelial progenitor cells express LYVE1, which does not exclude the possibility of differentiation into LECs. Although we have rejected the possibility that lymphatic vessels originate from the bone marrow, the contribution of circulating non-bone marrow-derived cells to the cardiac lymphatic sremains to be illustrated. In the future, lineage tracing mice combined with parabiosis experiments will be needed to further investigate the potential of circulating LEC progenitors in lymphangiogenesis after heart transplantation.

In summary, our results describe the process of lymphatic regeneration in mouse heart allografts, with the gradual appearance of myocardial lymphatic vessels post-transplantation. These newly formed lymphatics are mainly from the recipient and LYVE1^+^ cells are the major contributors to the cardiac lymphatic vessels. Activated fibroblasts (not myofibroblasts) are widely appeared in the transplanted heart and are shown to be the major regulatory cells of LECs after transplantation. They facilitate lymphangiogenesis mainly by secreting VEGFD, MDK and SEMA3C, which acts on their corresponding receptors on LECs. Ablation of activated fibroblasts and knocking down VEGFD and MDK of activated fibroblasts leads to a reduction of cardiac lymphatic vessels, which is related to the survival of transplanted heart. Therefore, manipulation of lymphangiogenesis or lymphatic vessel formation could be a potential target for maintaining the function of transplanted heart.

## Supplementary Material

Supplementary figures and legends, and additional higher-quality versions of the supplementary figures.

## Abbreviations

BM: bone marrow
CsA: cyclosporine A
DTA: diphtheria toxin subunit A
ECs: endothelial cells
ECM: extracellular matrix
FACS: fluorescence-activated cell sorting
IL-1β: interleukin 1β
IL-6: interleukin 6
LECs: lymphatic endothelial cells
LYVE1: lymphatic vessel endothelial hyaluronan receptor 1
MDK: midkine
NCL: nucleolin
NRP2: neuropilin 2
POSTN: periostin
PTN: pleiotrophin
scRNA-seq: single-cell RNA sequencing
SEMA3C: semaphorin 3C
TNF-α: tumor necrosis factor α
VEGFC: vascular endothelial growth factor C
VEGFD: vascular endothelial growth factor D
VEGFR3/FLT4: Fms-related tyrosine kinase 4
