# Supplementary material for: Fibroblasts facilitate lymphatic vessel formation in transplanted heart
Source: Theranostics. 2024 Feb 24;14(5):1886–908. doi: 10.7150/thno.92103 (PMC10945330; doi:10.7150/thno.92103)
Supplement: Supplementary file 1 — Supplementary figures and legends, and additional higher-quality versions of the supplementary figures. [file thnov14p1886s1.pdf]

Supplementary Material

Supplemental Figures

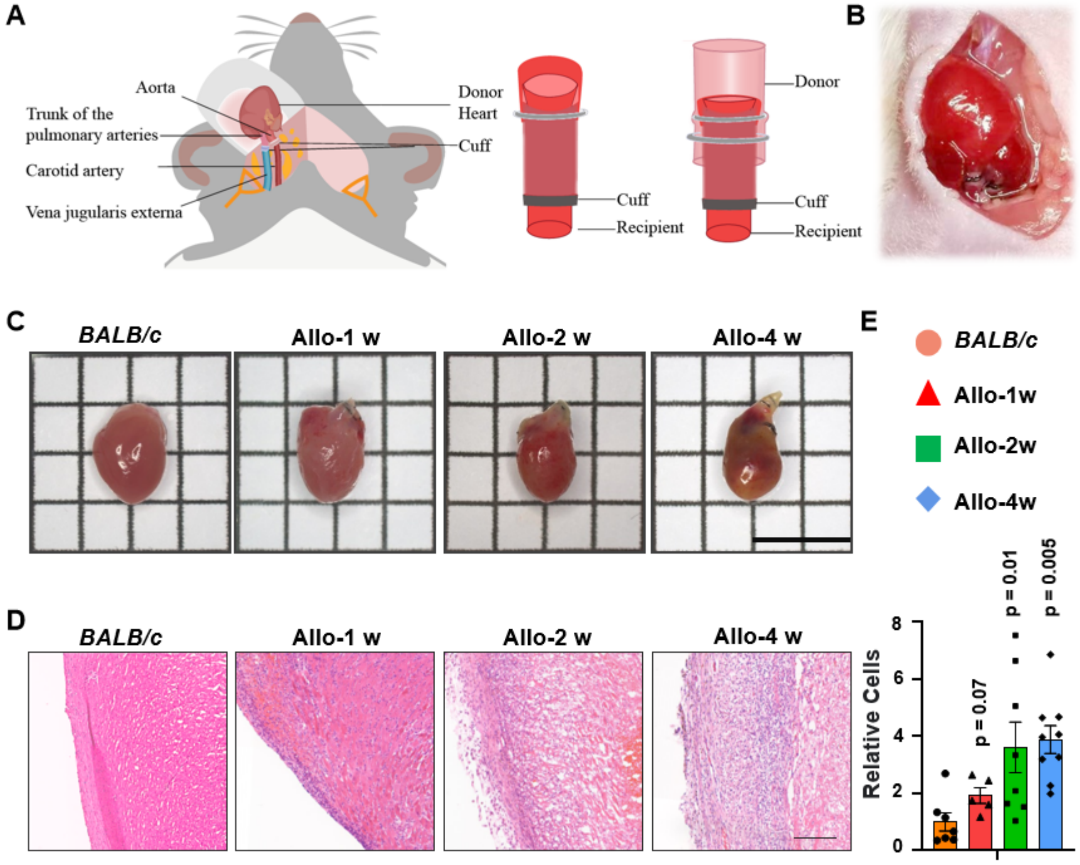

Figure S1. **A**, Sketch of the mouse heterotopic cervical heart transplantation. **B**, Construction of the mouse heterotopic cervical heart transplantation model. **C**, Cardiac allografts at indicated time points. **D**, HE staining of cardiac allograft slices at indicated time points. **E**, Quantification of relative cell number, cell number of BALB/c group as the criterion (1.0); Number of mice in each group: n = 7(control), n = 5 (Allo-1 w) , n = 8 (Allo-2 w), n = 9 (Allo-4 w), one-way ANOVA test. Scale bars, 1 mm (C), and 200 μm (D).

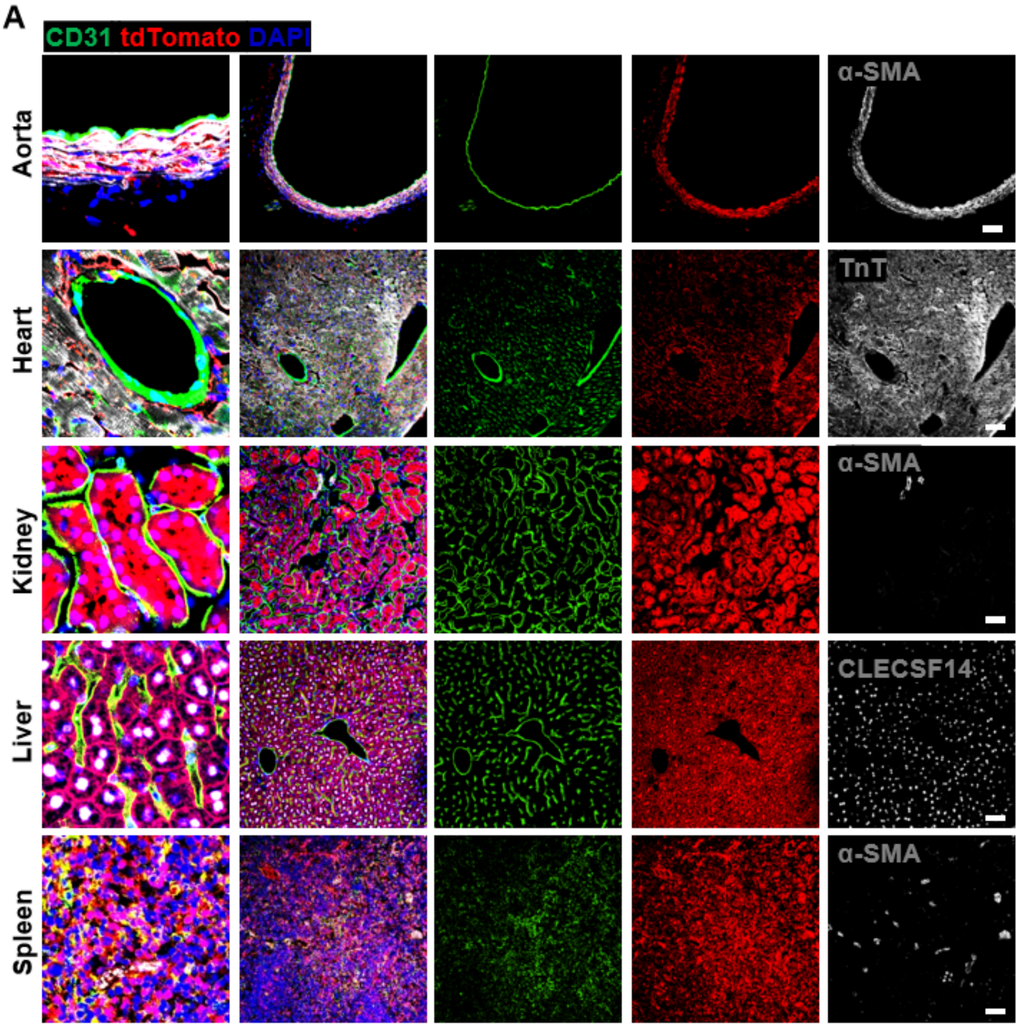

Figure S2. **A**, Immunofluorescence staining to verify the efficiency of tdTomato-labeled CAG+ cells in Cag-Cre;R26-tdTomato mice. Scale bar: 100 μm.

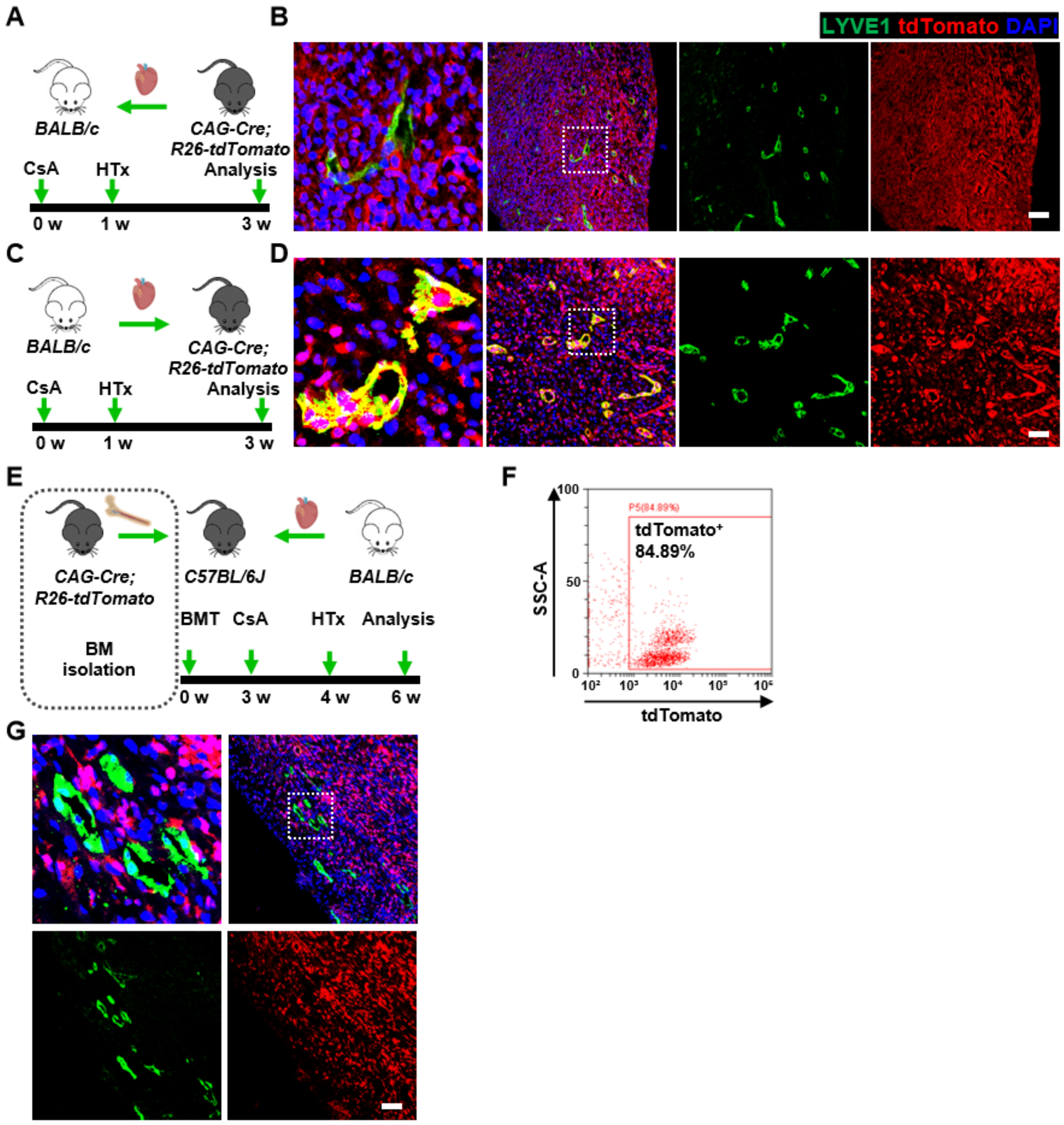

Figure S3. **A**, Schematic of heart transplantation model: Donor heart from Cag-Cre;R26-tdTomato mouse was transplanted into BALB/c recipient mouse. **B**, Immuno-staining of lymphatic vessels in allograft heart 2 weeks after transplantation which was described in (**A**).The images within the dashed box are displayed on the left. **C**, Schematic of heart transplantation model: Donor heart from BALB/c mouse was transplanted into Cag-Cre;R26-tdTomato recipient mouse. **D**, Immuno-staining of lymphatic vessels in allograft heart 2 weeks after transplantation which was described in (**C**). **E**, Schematic of heart transplantation model: Bone marrow (BM) of C57BL/6J recipient mice was replaced with the BM harvested from Cag-Cre;R26-tdTomato mice, subsequently the donor hearts from BALB/c mice were transplanted into C57BL/6J recipient mice. **F**, Quantification of tdTomato positive cells by flow cytometry in the reconstructed BM of recipient mice before heart transplantation which was described in (**E**). **G**, Immuno-staining of lymphatic vessels in allograft heart 2 weeks after transplantation which was described in (**E**).The image within the dashed box is displayed on the right. Scale bars, 100 μm (B, D and G).

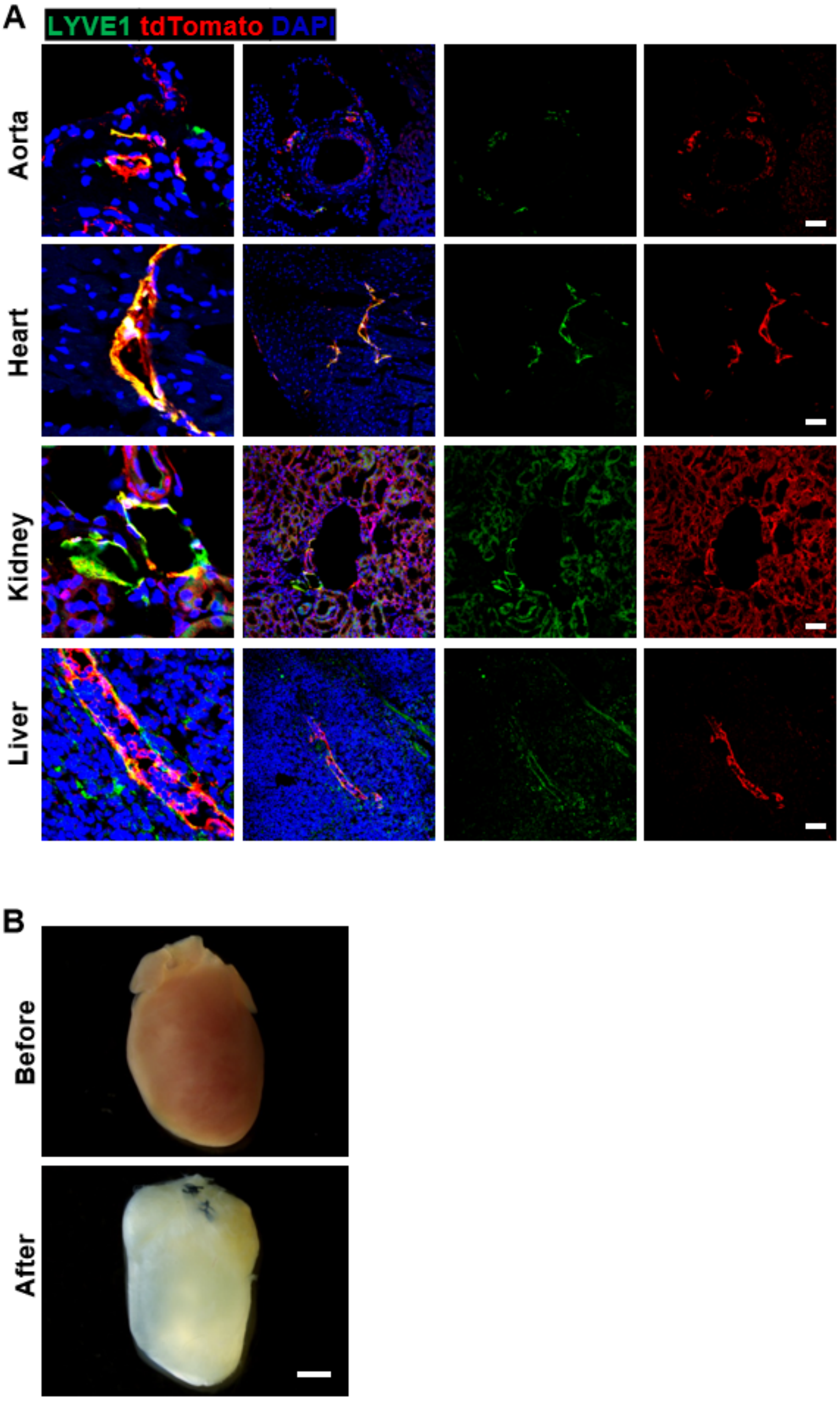

Figure S4. **A**, Immunofluorescence staining to verify the efficiency of tdTomato-labeled LYVE1+ cells in Lyve1-CreERT2;R26-tdTomato mice. **B**, Whole heart clearing with CUBIC. Scale bar: 100 μm (A), 1 mm (H).

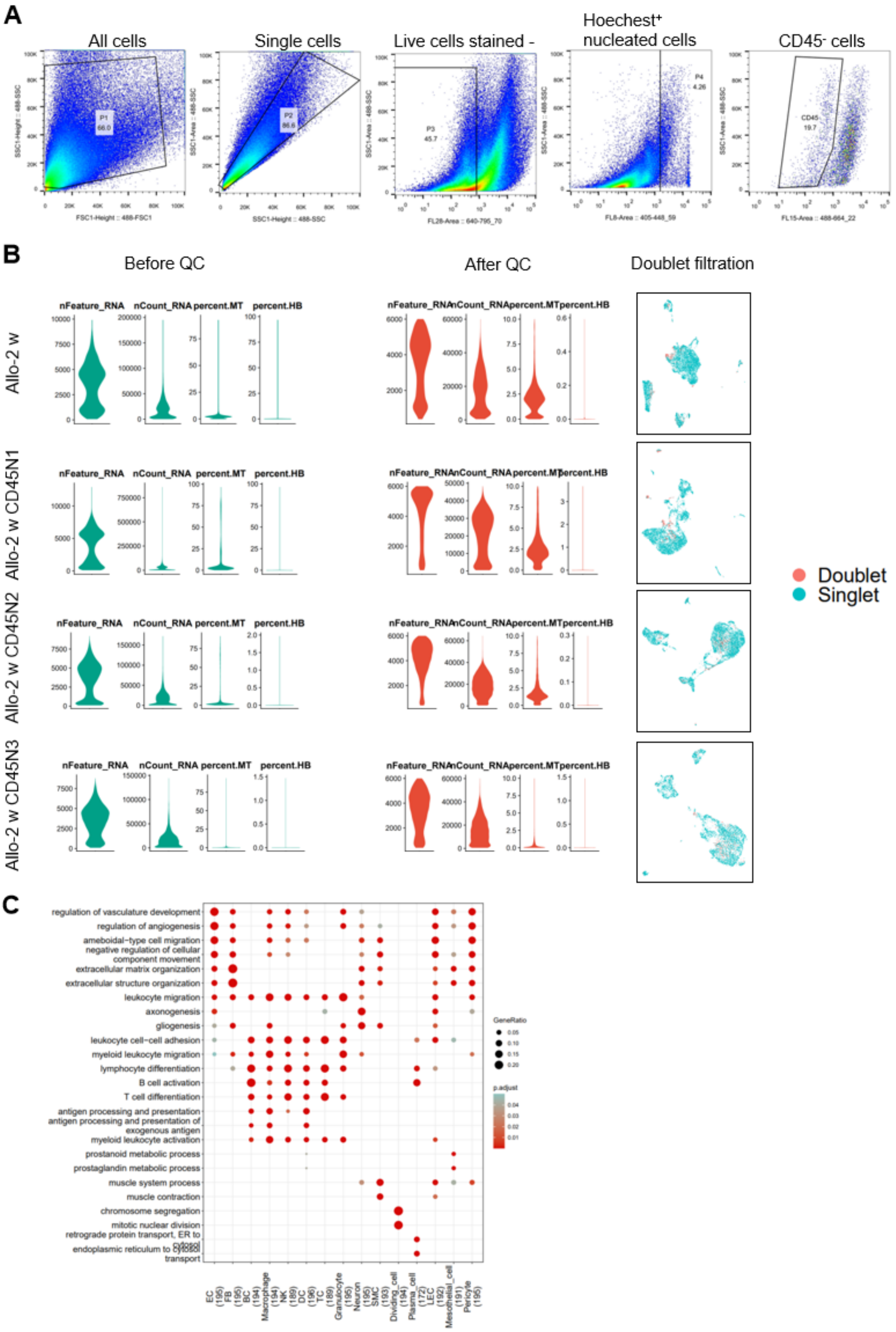

Figure S5. **A**, Flow cytometry of sorting strategy. **B**, Quantity control of single-cell RNA sequencing data. C, Dot plot shows the GO enrichment terms of major cell types.

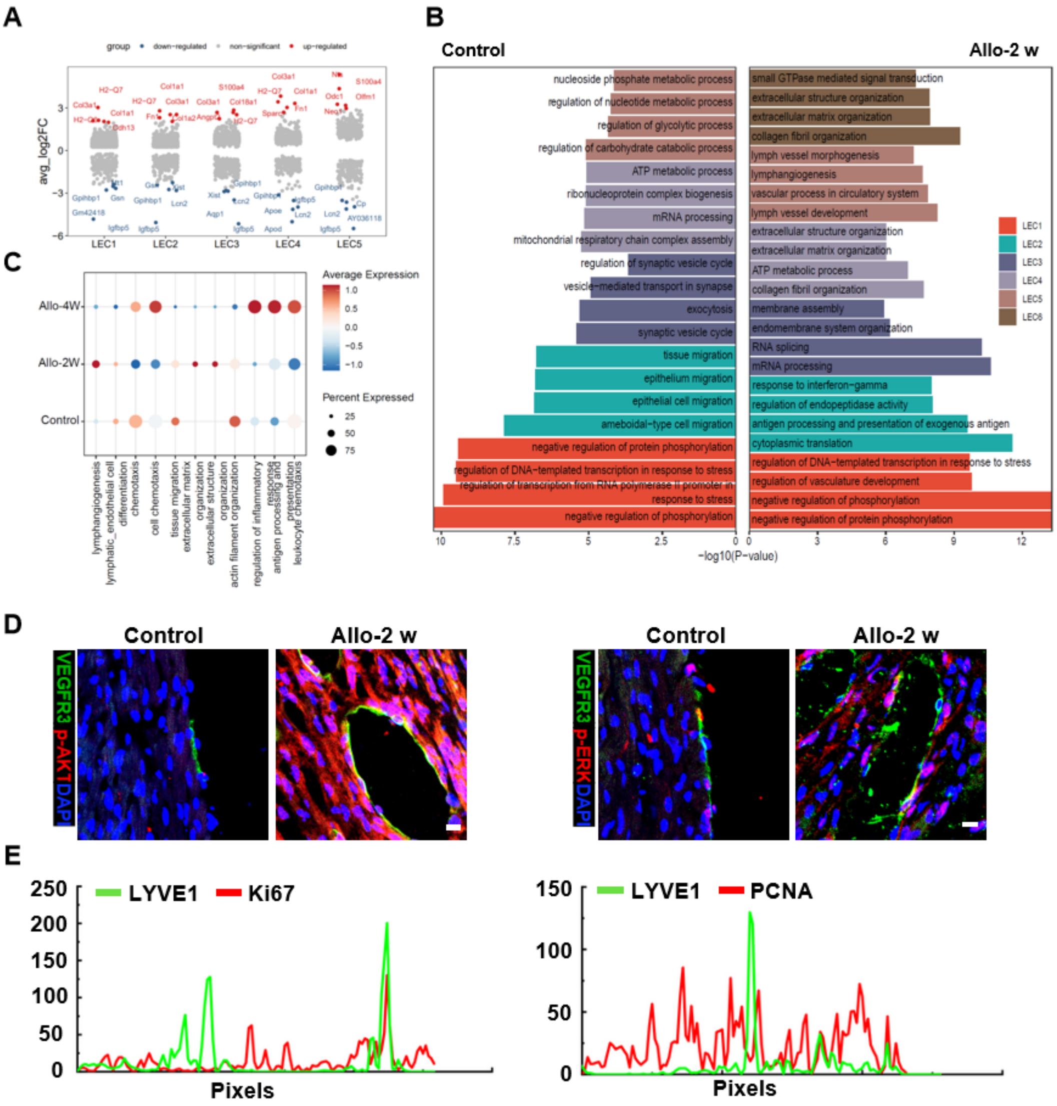

Figure S6. **A**, Volcano plot shows DEGs between two groups. **B**, Bar plot shows GO enriched in LEC sub-clusters from two groups. **C**, Dot plot shows GO enrichment of DEGs in different groups. **D**, Immuno-staining of p-AKT (left) and p-ERK (right) in LECs from two groups. **E**, Image J analysis of co-immuno staining of Ki67 (left) and PCNA (right) with LYVE1 in the cardiac allograft group. Scale bars, 25 μm (D).

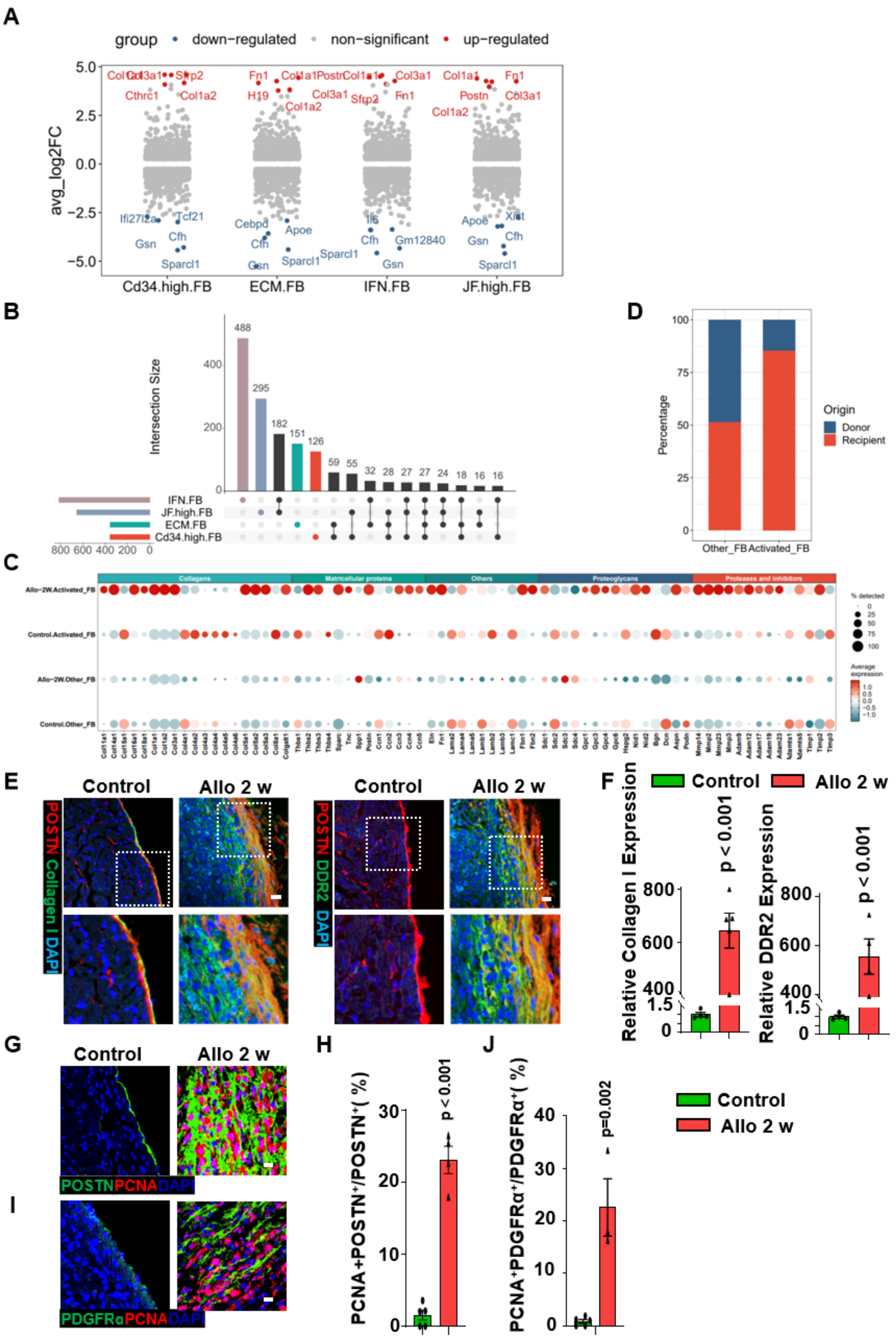

Figure S7. **A**, Volcano plot shows differentially expressed genes (DEGs) between allo-2 w and control group, genes in red represents up-regulated expression in allo-2 w group, genes in blue represents down-regulated expression in control group. **B**, Upset plot shows gene ontology (GO) terms enriched in each sub-cluster. The GO terms were enriched according to top 200 DEGs of each sub-cluster, and the terms with p value < 0.01 were used for further analysis. **C**, Dot plot

shows the expression of genes associated with ECM metabolism in two fibroblast sub-populations. **D**, Bar plot shows the proportions of donor-derived and recipient derived cells in two fibroblast sub-populations. **E**, **F**, Immuno-staining (**E**) and quantification (**F**) of Collagen I and DDR2 expression in control group and allograft group. The images within the dashed box are displayed in the second row. The area of POSTN+Collagen I+ or POSTN+DDR2+ in control group as the criterion (1.0); Number of mice in group (Collagen I): n = 4 (control), n = 5 (Allo-2 w); Number of mice in group (DDR2): n = 4; unpaired t-test. **G**, **H**, Immunostaining (**G**) and quantification (**H**) of proliferation marker (PCNA) in cardiac fibroblasts (POSTN+) of two groups; Number of mice in each group: n = 5 (control), n = 4 (Allo-2 w), unpaired t-test. **I**, **J**, Immunostaining (**I**) and quantification (**J**) of proliferation marker (PCNA) in cardiac fibroblasts (PDGFRα+) of two groups; Number of mice in each group: n = 5 (control), n = 3 (Allo-2 w), unpaired t-test. Scale bars, 100 μm (E, G, I).

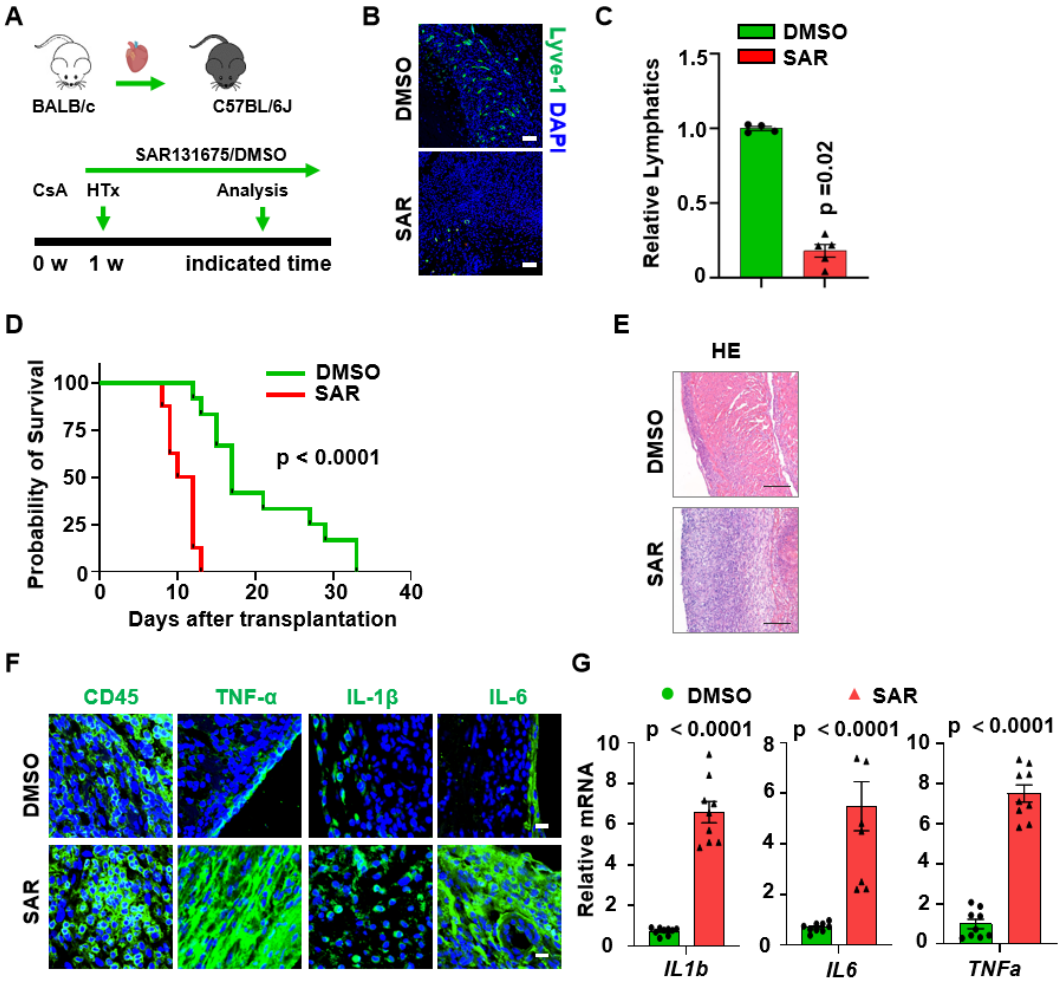

Figure S8. **A**, Sketch of the experimental design for lymphatic vessel growth inhibition after heart transplantation: VEGFR3 inhibitor (SAR131675) was used to suppress lymphatic vessel formation (SAR group), while DMSOl solution (5% DMSO in 30% PEG 300+5% Tween 80+ddH2O solution) was used as control group. **B**, **C**, Immunostaining (**B**) and quantification (**C**) of lymphatic vessels of cardiac allografts in two groups after 12 days of transplantation; Number of mice in each group: n = 4 (DMSO), n = 5 (SAR); unpaired t-test. **D**, Survival time of cardiac allografts in two groups; Number of mice in each group: n = 12 (DMSO), n = 8 (SAR); Gehan-Breslow-Wilcoxon test. **E**, HE staining of cardiac allograft slices in two groups. **F**, Immunostaining assay of CD45, TNF-α, IL-1β and IL-6 expression in cardiac allografts from two groups. **G**, qPCR quantification of IL-1b, IL-6 and TNFa in cardiac allografts of two groups, mRNA level in the DMSO group as the criterion (1.0); Number of mice in each group (IL1b, TNFa): n = 9; Number of mice in group (IL6): n = 9 (DMSO), n = 7 (SAR); unpaired t-test. Scale bars, 50 μm (B), 200 μm (E), 25 μm (F).

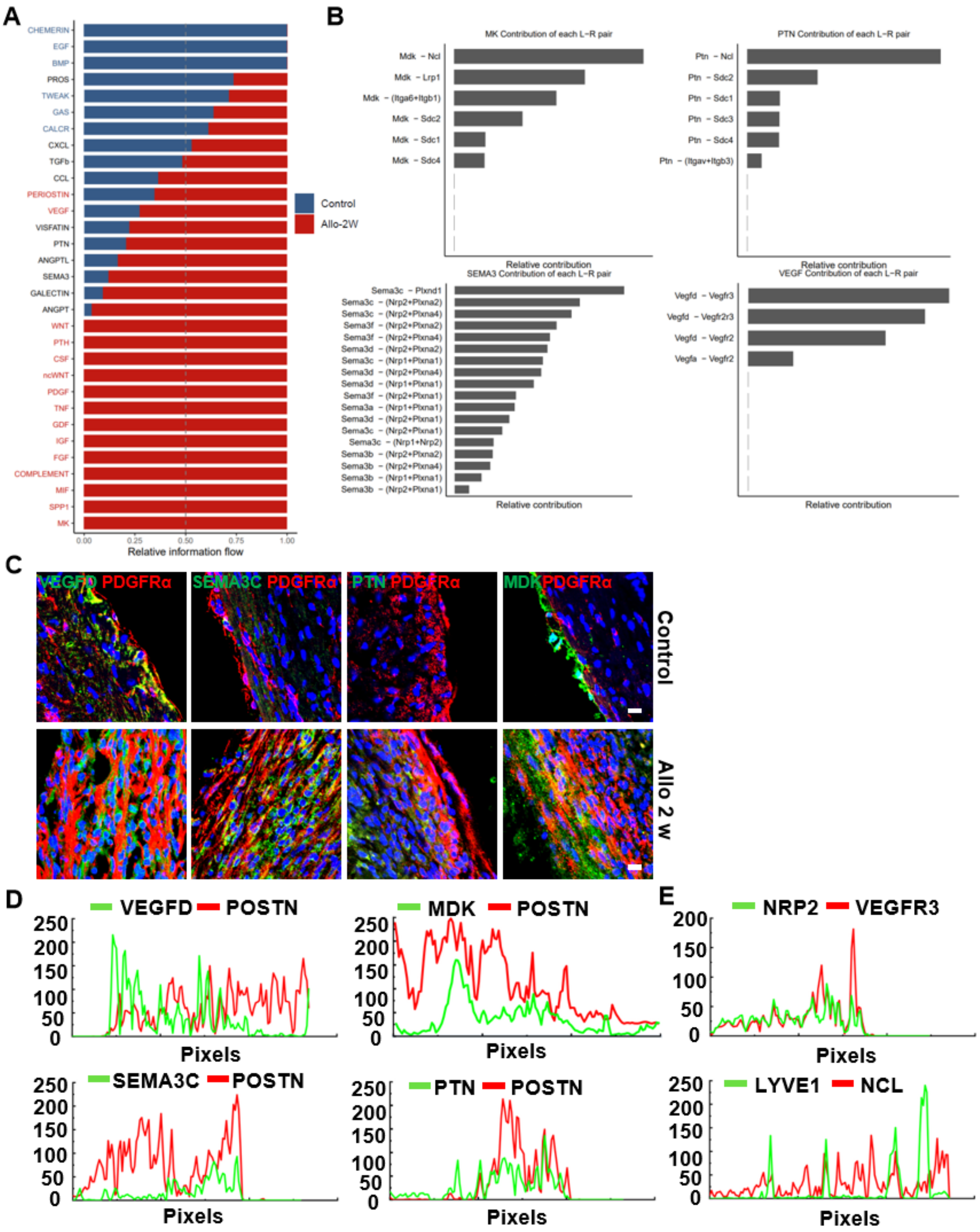

Figure S9. **A**, Bar plot shows the rank of cell-cell communication patterns in two groups. **B**, Bar plot shows the contribution of ligand-receptor pairs in selected signaling patterns. **C**, Immuno-staining of ligands in PDGFRα+ fibroblasts of the cardiac allografts from two groups. **D**, Image J analysis of co-immuno staining of potential ligands (VEGFD, MDK, SEMA3C and PTN) with POSTN in allograft group. E, Image J analysis of co-immuno staining of NRP2 with VEGFR3, and NCL with LYVE1 in allograft group. Scale bars, 25 μm (C).

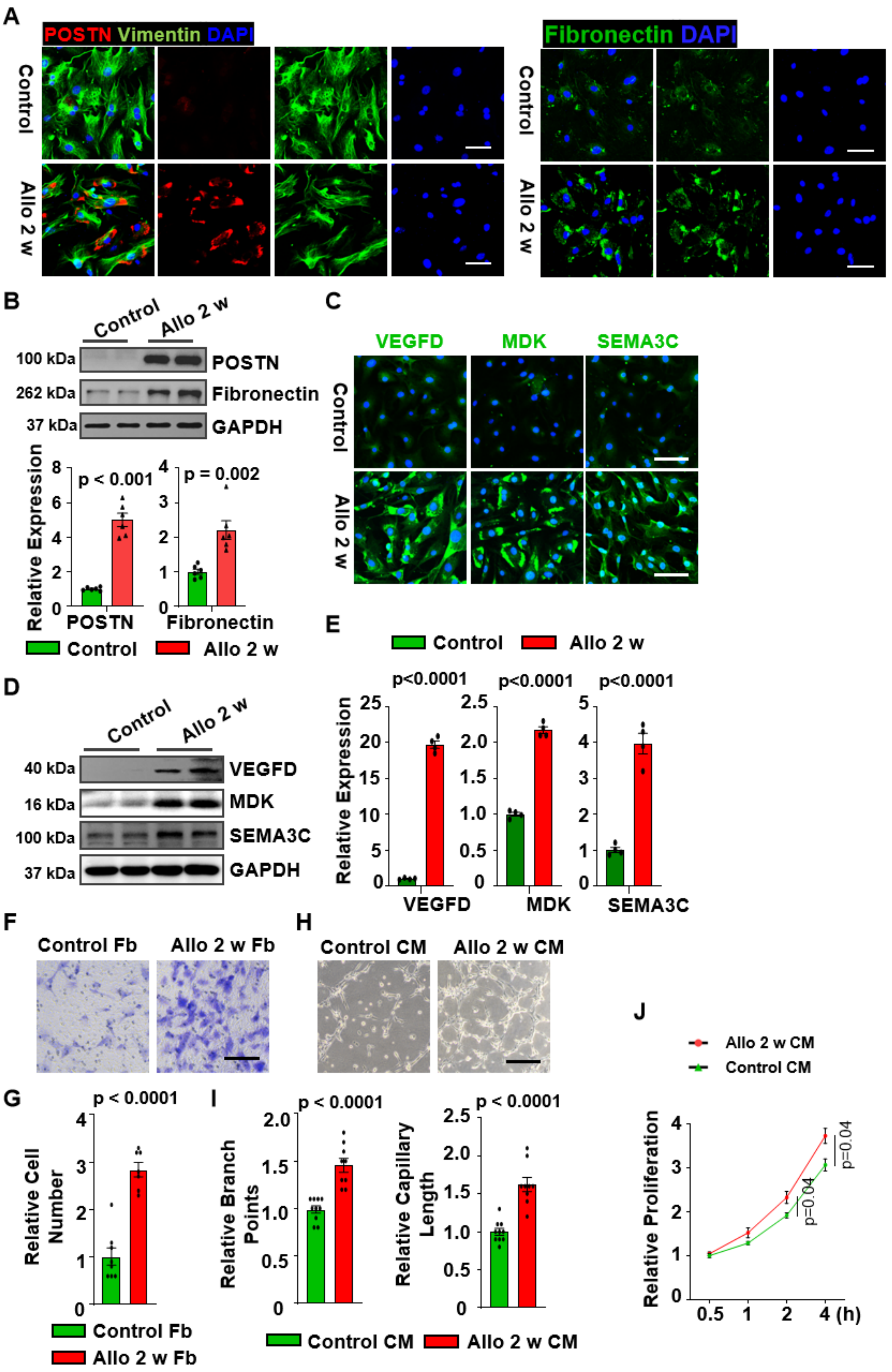

Figure S10. **A**, Immunostaining of POSTN, Vimentin (left panel) and Fibronectin (right panel) in fibroblasts isolated from normal hearts and allograft hearts after 2 weeks of transplantation. **B**, Western blotting assay (up panel) and quantification (down panel) of POSTN and Fibronectin expression in control fibroblasts and allograft fibroblasts, the protein expression in the control

group as the criterion (1.0); Number of mice in each group: n = 6, unpaired t-test. **C**, Immunostaining of ligands (VEGFD, MDK and SEMA3C) in control fibroblasts and allograft fibroblasts. **D**, **E**, Western blotting assay (**D**) and quantification (**E**) of VEGFD, MDK and SEMA3C in the fibroblasts from two groups, the protein expression in the control group as the criterion (1.0); Number of mice in each group: n = 4, unpaired t-test. **F**, **G**, Transwell assay (**F**) and quantification (**G**) of LECs co-cultured with fibroblasts from two groups, cell number in the control group as the criterion (1.0); Replication number in each group (from four heart isolation): n = 8 (Control Fb), n = 7 (Allo 2 w Fb) , unpaired t-test. **H**, **I**, Tube formation assay (**H**) and quantification (**I**) of LECs cultured with fibroblasts conditioned medium of two groups, the branch points and the capillary length in the control group as the criterion (1.0); Replication number in each group (from four heart isolation): n = 10 (Control CM), n = 9 (Allo 2 w CM), unpaired t-test. **J**, CCK8 assay of LECs cultured with fibroblasts conditioned medium of two groups; Replication number in each group (from four heart isolation): n = 6, unpaired t-test. Scale bars, 25 μm (A, C), 50 μm (F), and 200 μm (H).

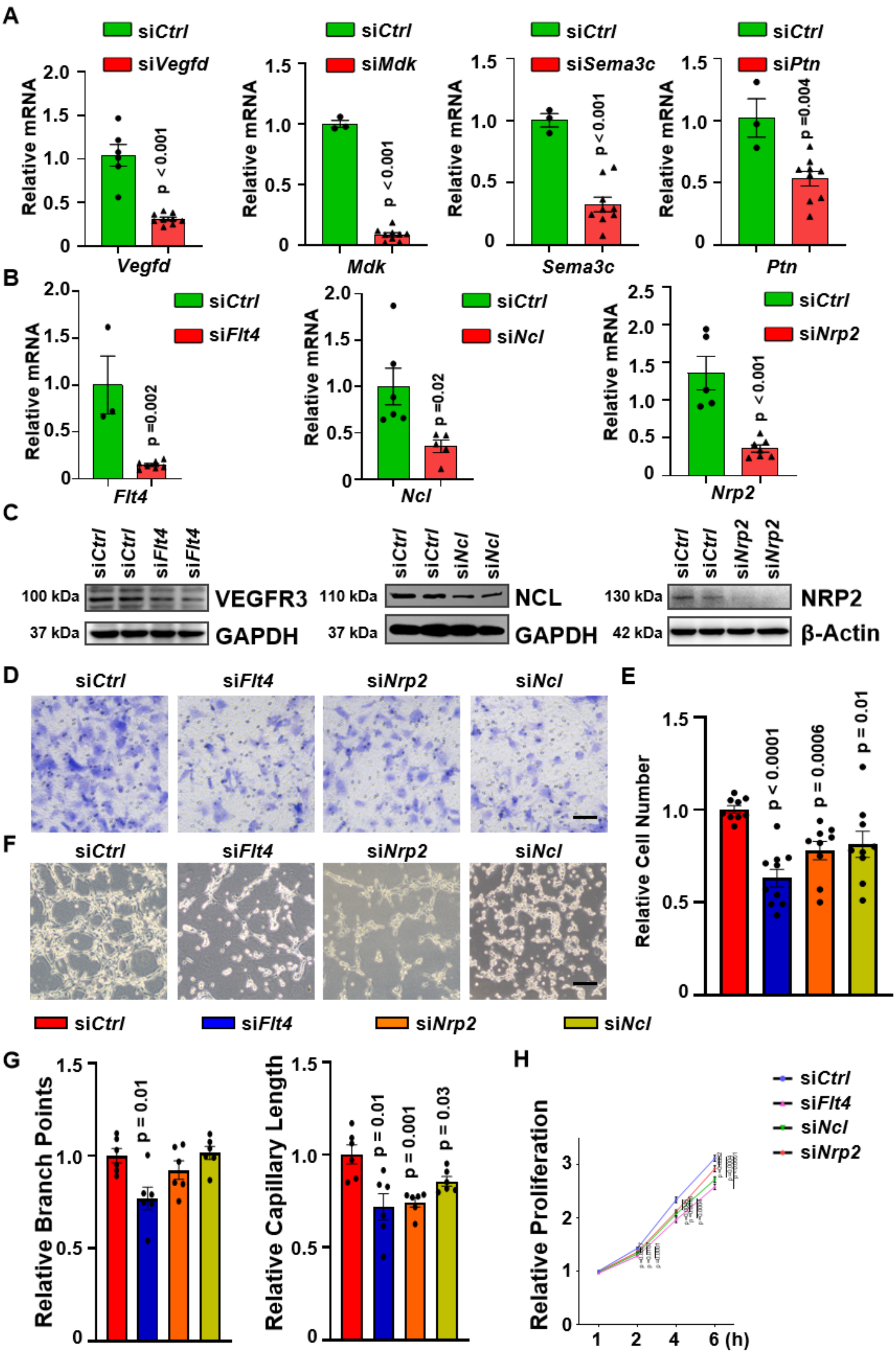

Figure S11. **A**, qPCR quantification of Vegfd, Mdk, Sema3c and Ptn in the transplanted heart fibroblasts treated with siRNA, mRNA expression in the control group as the criterion (1.0); Cells were isolated from four heart isolation, replication number in group (Vegfd): n = 6 (siCtrl), n = 9 (siVegfd); group (Mdk/Sema3c/Ptn): n = 3 (siCtrl), n = 9 (siMdk/siSema3c/siPtn); unpaired t-test.

B, qPCR quantification of Flt4, Ncl and Nrp2 in mouse LECs treated with siRNA, mRNA expression in the control group as the criterion (1.0); Replication number in group (Flt4): n = 3 (siCtrl), n = 7 (siFlt4); group (Ncl): n = 6 (siCtrl), n = 5 (siNcl); group (Nrp2): n = 5 (siCtrl), n = 7 (siNrp2); unpaired t-test. **C**, Western blotting assay of Flt4, Ncl and Nrp2 in mouse LECs treated with siRNA. **D**, **E**, Transwell assay (**D**) and quantification (**E**) of LECs (treated with siRNA) co-cultured with allograft fibroblasts, cell number in the control group as the criterion (1.0); Number in each group: n = 9 (siCtrl), n = 10 (siFlt4), n = 9 (siNrp2), n = 9 (siNcl), one-way ANOVA test. **F**, **G**, Tube formation assay (**F**) and quantification (**G**) of LECs (treated with siRNA) cultured with allograft fibroblasts conditioned medium, the branch points and the capillary length in the control group as the criterion (1.0); n = 6, one-way ANOVA test. **H**, CCK8 assay of LECs (treated with siRNA) cultured with allograft fibroblasts conditioned medium; n = 6, one-way ANOVA test. Scale bars, 50 μm (D), and 200 μm (F).

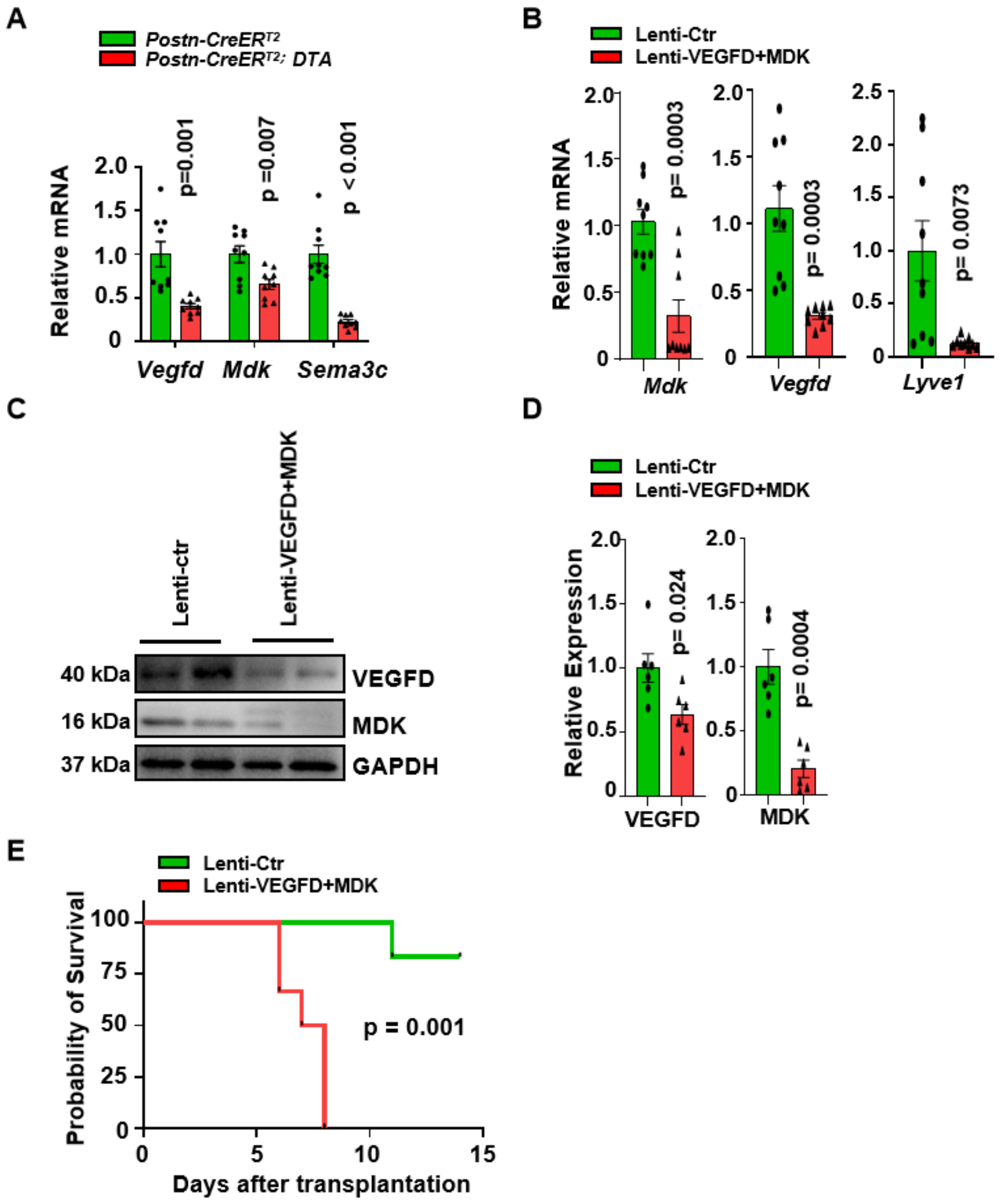

Figure S12. **A**, mRNA expression of secreted fibroblast ligand genes (Vegfd, Mdk and Sema3c) in the hearts from the POSTN+ cell depletion group (Postn-CreERT2;DTA) and the control group (Postn-CreERT2), mRNA expression in the control group as the criterion (1.0); Number of mice in each group: n = 9, unpaired t-test. **B**, qPCR quantification of Vegfd, Mdk and Lyve1 in the cardiac allografts of the ligands-knockdown group and the control group, mRNA expression in the control group as the criterion (1.0); Number of mice in each group: n = 9, unpaired t-test. **C**, **D**, Western blotting assay and quantification of VEGFD and MDK expression in ligands-knockdown group and the control group, protein expression in the control group as the criterion (1.0); Number of mice in each group: n = 6, unpaired t-test. **E**, Survival time of cardiac allografts in VEGFD/MDK-knockdown group and the control group; Number of mice in each group: n = 6, Gehan-Breslow-Wilcoxon test.

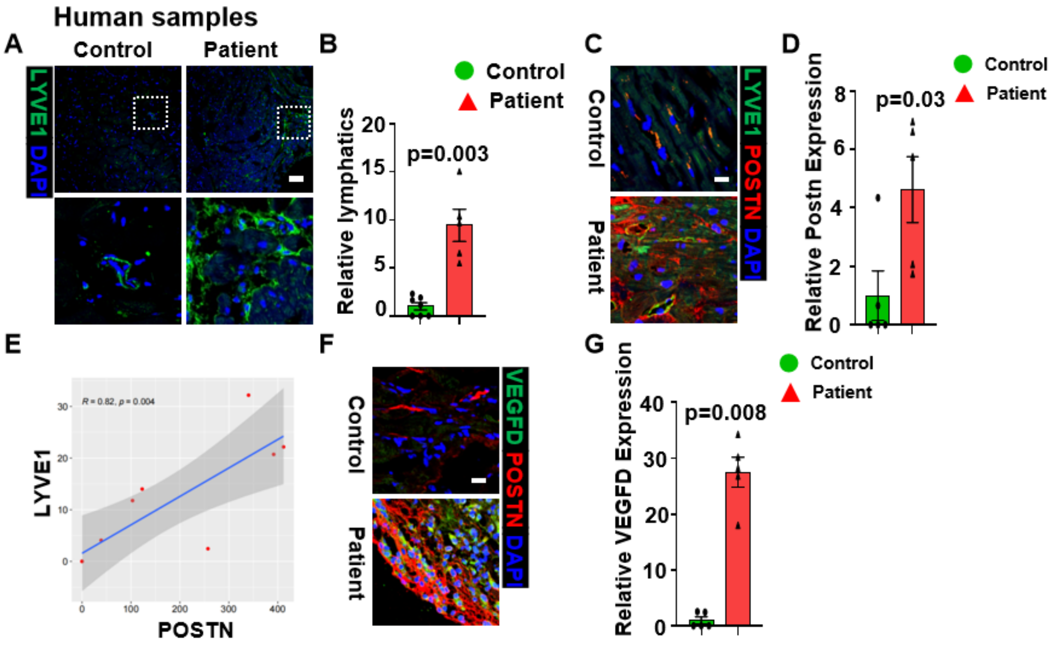

Figure S13. **A**, **B**, Immunostaining (**A**) and quantification (**B**) of cardiac lymphatic vessels in heart tissue of healthy people (control group) and the patients undergone the heart transplant operation, the lymphatic vessel density of the control group as the criterion (1.0); n = 7 (Control), n = 5 (Patient), unpaired t-test. **C**, **D**, Immunostaining (**C**) and quantification (**D**) of activated fibroblasts in two human groups, the area of POSTN+ expression in control group as the criterion (1.0); n = 5, unpaired t-test. **E**, Pearson correlation coefficient of POSTN and LYVE1 in human transplanted heart; n = 5. **F**, **G**, Immunostaining (**F**) and quantification (**G**) of VEGFD secreted by activated fibroblasts in two human groups, the area of POSTN+VEGFD+ expression in control group as the criterion (1.0); n = 5, unpaired t-test. Scale bars, 100 µm (A), 25 µm (C,F).

Figure S1

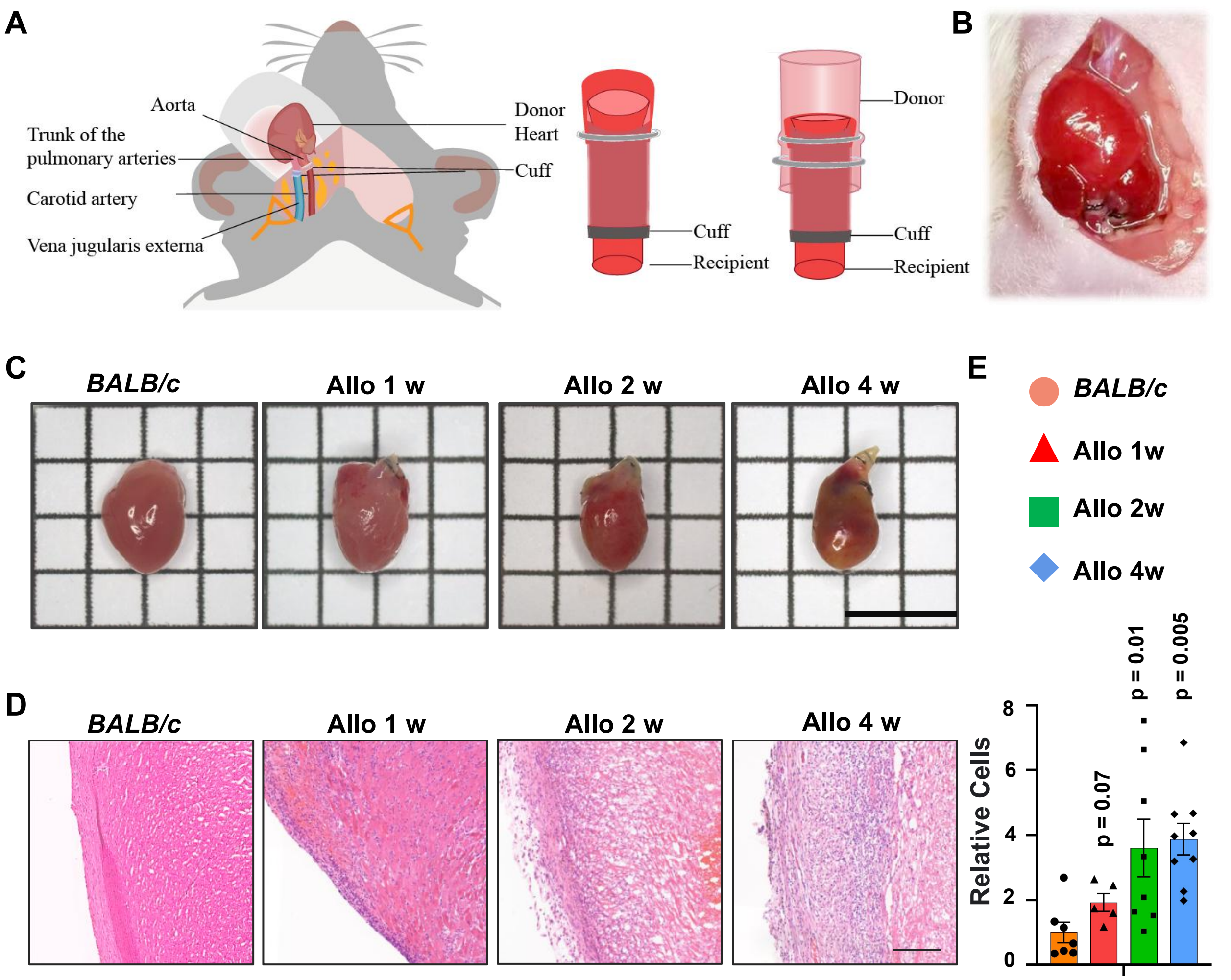

A
Aorta
Trunk of the
pulmonary arteries
Carotid artery
Vena jugularis externa
Donor
Heart
Cuff
Cuff
Recipient
Donor
Cuff
Recipient
B
C
BALB/c
Allo 1 w
Allo 2 w
Allo 4 w
D
BALB/c
Allo 1 w
Allo 2 w
Allo 4 w
E
BALB/c
Allo 1w
Allo 2w
Allo 4w
Relative Cells
p = 0.07
p = 0.01
p = 0.005
8
6
4
2
0

Figure S2

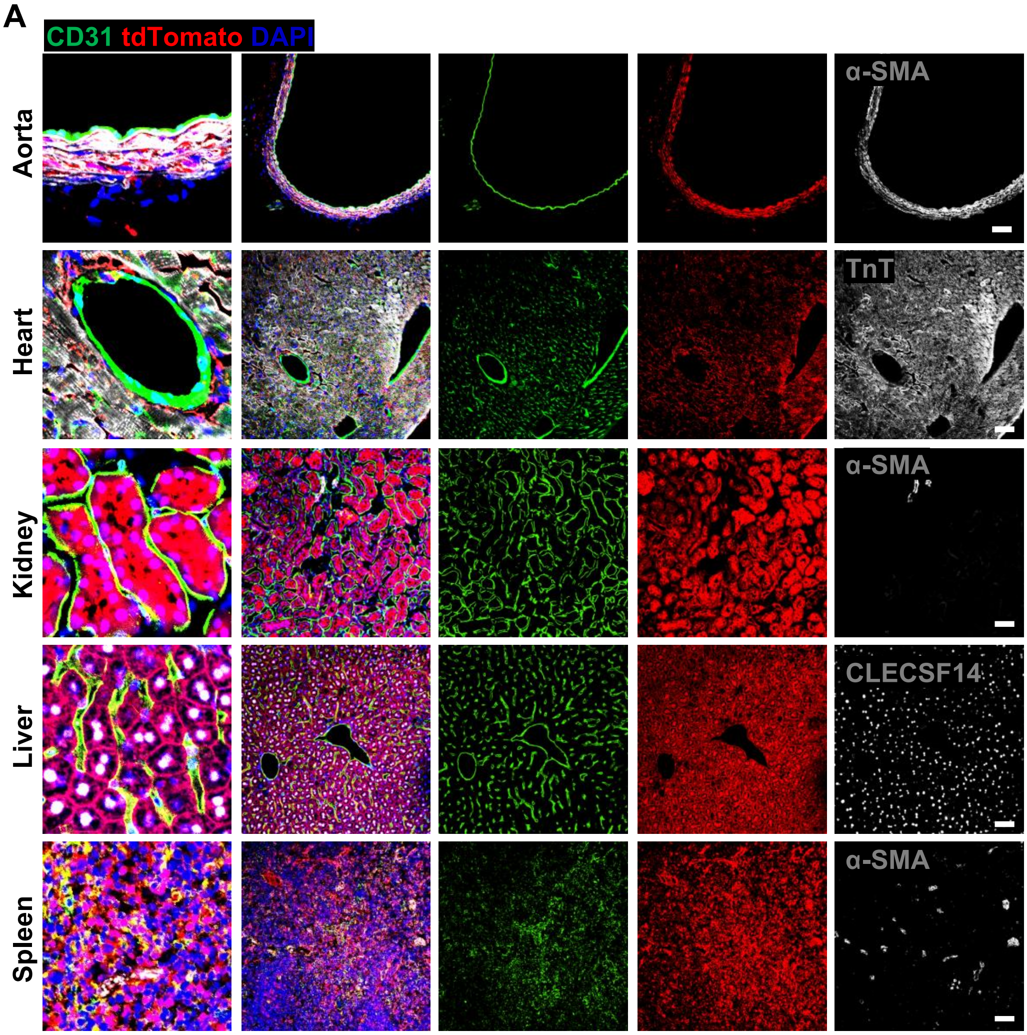
A
CD31 tdTomato DAPI
Aorta
Heart
Kidney
Liver
Spleen
α-SMA
TnT
α-SMA
CLECSF14
α-SMA

Figure S3

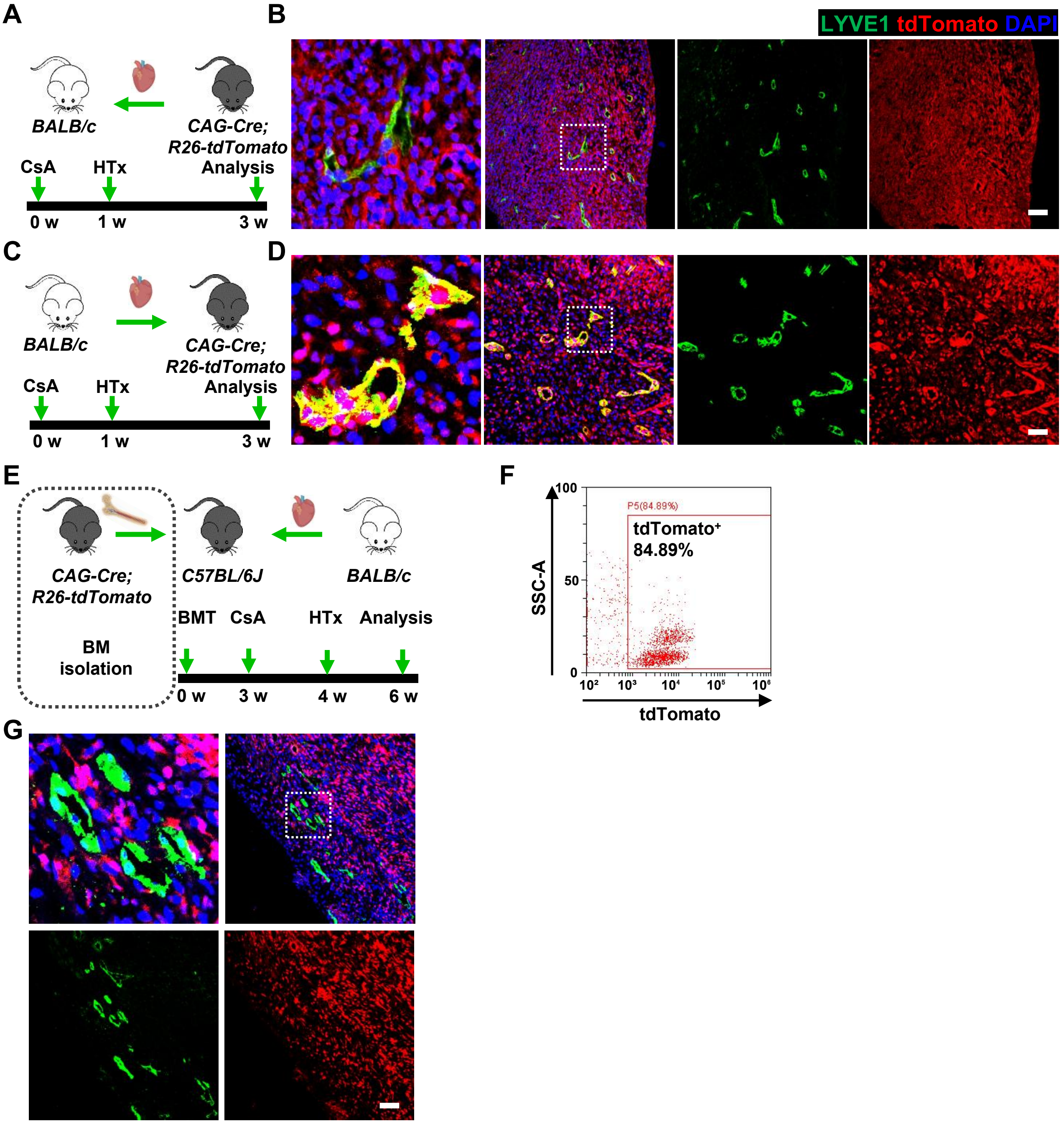
A
B
LYVE1 tdTomato DAPI
BALB/c
CAG-Cre; R26-tdTomato
CsA
HTx
Analysis
0 w
1 w
3 w
C
D
BALB/c
CAG-Cre; R26-tdTomato
CsA
HTx
Analysis
0 w
1 w
3 w
E
CAG-Cre; R26-tdTomato
BM isolation
C57BL/6J
BALB/c
BMT
CsA
HTx
Analysis
0 w
3 w
4 w
6 w
F
P5(84.89%)
tdTomato+ 84.89%
SSC-A
100
50
0
tdTomato
G

Figure S4

A

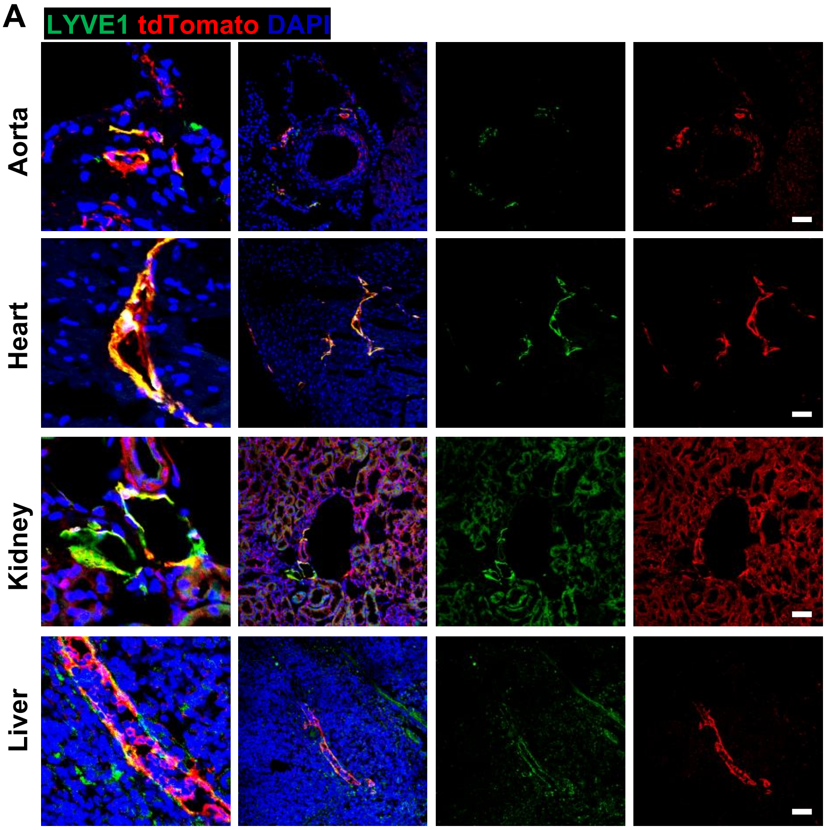

B

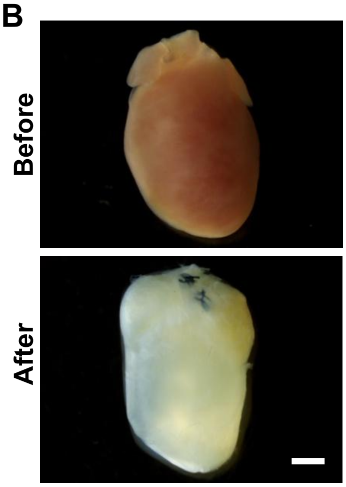

Figure S5

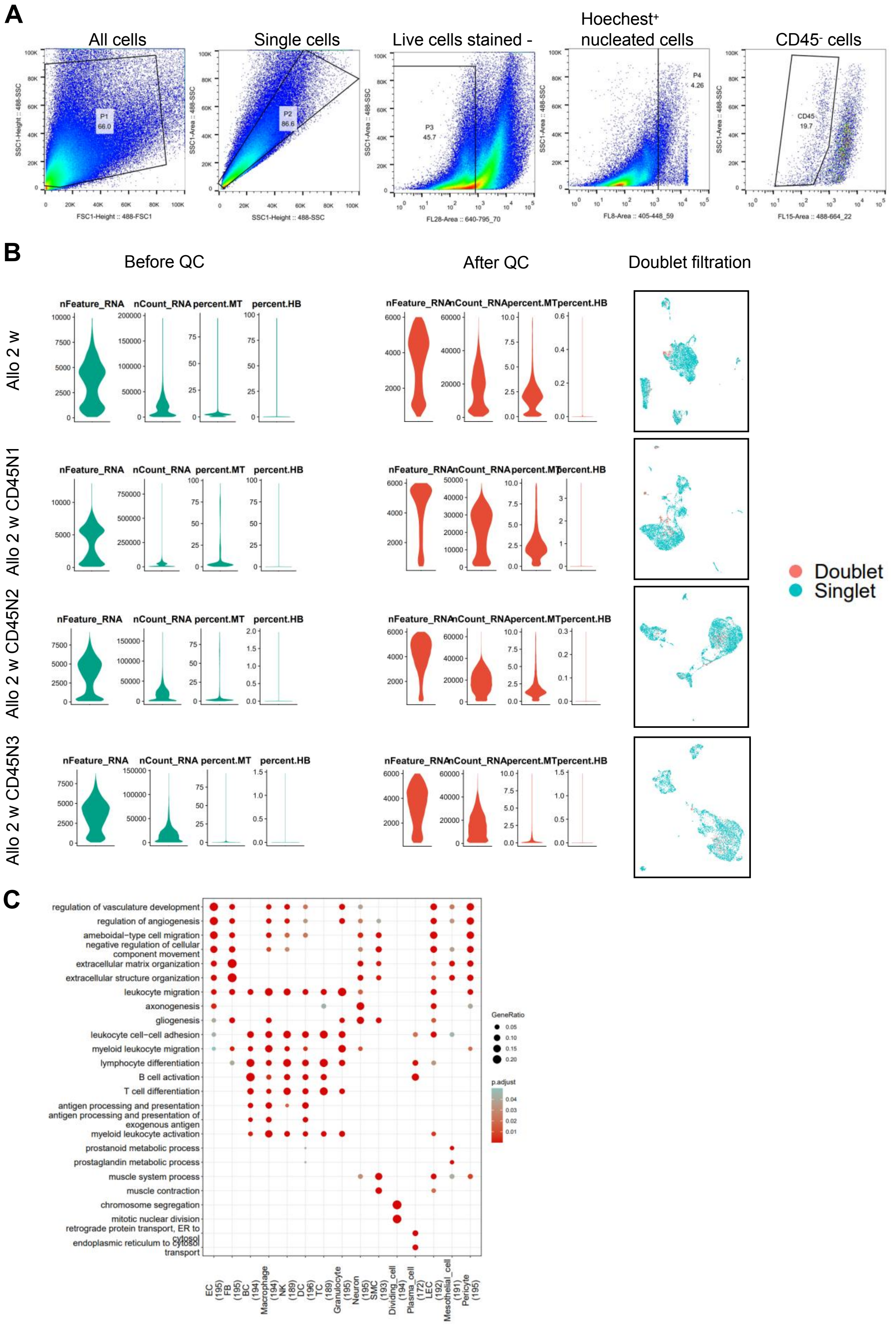
A
All cells
Single cells
Live cells stained -
Hoechest+ nucleated cells
CD45- cells
B
Before QC
After QC
Doublet filtration
Allo 2 w
Allo 2 w CD45N1
Allo 2 w CD45N2
Allo 2 w CD45N3
nFeature_RNA
nCount_RNA
percent.MT
percent.HB
Doublet
Singlet
C
regulation of vasculature development
regulation of angiogenesis
ameboidal−type cell migration
negative regulation of cellular component movement
extracellular matrix organization
extracellular structure organization
leukocyte migration
axonogenesis
gliogenesis
leukocyte cell−cell adhesion
myeloid leukocyte migration
lymphocyte differentiation
B cell activation
T cell differentiation
antigen processing and presentation
antigen processing and presentation of exogenous antigen
myeloid leukocyte activation
prostanoid metabolic process
prostaglandin metabolic process
muscle system process
muscle contraction
chromosome segregation
mitotic nuclear division
retrograde protein transport, ER to cytosol
endoplasmic reticulum to cytosol transport
GeneRatio
p.adjust
EC (195)
FB (195)
BC (194)
Macrophage (194)
NK (189)
DC (196)
TC (189)
Granulocyte (195)
Neuron (195)
SMC (193)
Dividing_cell (194)
Plasma_cell (172)
LEC (192)
Mesothelial_cell (191)
Pericyte (195)

Figure S6

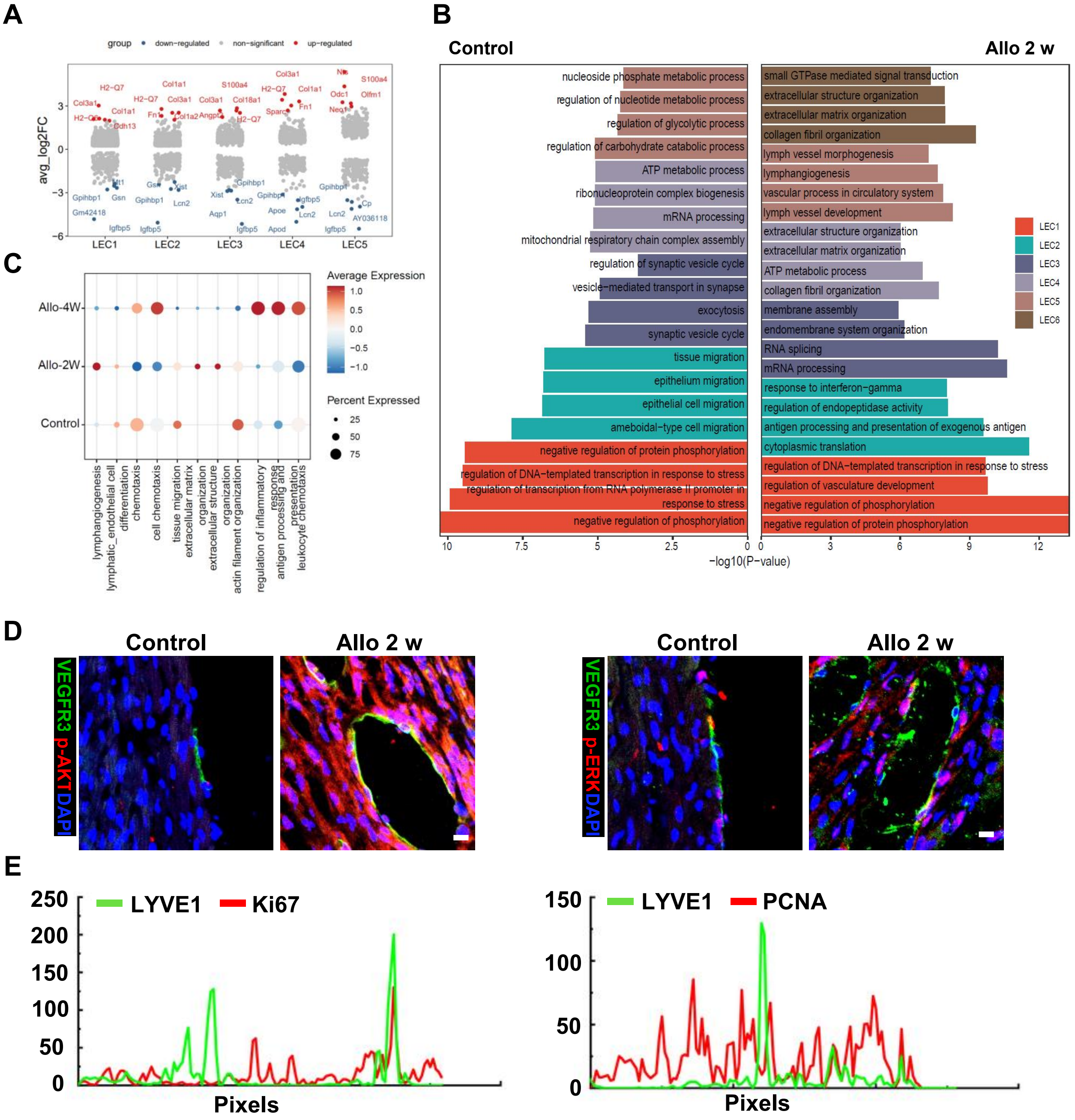
A
B
C
D
E
group down−regulated non−significant up−regulated
avg_log2FC
LEC1
LEC2
LEC3
LEC4
LEC5
Control
Allo 2 w
nucleoside phosphate metabolic process
regulation of nucleotide metabolic process
regulation of glycolytic process
regulation of carbohydrate catabolic process
ATP metabolic process
ribonucleoprotein complex biogenesis
mRNA processing
mitochondrial respiratory chain complex assembly
regulation of synaptic vesicle cycle
vesicle−mediated transport in synapse
exocytosis
synaptic vesicle cycle
tissue migration
epithelium migration
epithelial cell migration
ameboidal−type cell migration
negative regulation of protein phosphorylation
regulation of DNA−templated transcription in response to stress
regulation of transcription from RNA polymerase II promoter in response to stress
negative regulation of phosphorylation
small GTPase mediated signal transduction
extracellular structure organization
extracellular matrix organization
collagen fibril organization
lymph vessel morphogenesis
lymphangiogenesis
vascular process in circulatory system
lymph vessel development
ATP metabolic process
membrane assembly
endomembrane system organization
RNA splicing
response to interferon−gamma
regulation of endopeptidase activity
antigen processing and presentation of exogenous antigen
cytoplasmic translation
regulation of vasculature development
negative regulation of phosphorylation
LEC6
−log10(P−value)
Allo-4W
Allo-2W
Control
Average Expression
Percent Expressed
lymphangiogenesis
lymphatic_endothelial cell differentiation
chemotaxis
cell chemotaxis
tissue migration
extracellular matrix organization
extracellular structure organization
actin filament organization
regulation of inflammatory response
antigen processing and presentation
leukocyte chemotaxis
VEGFR3 p-AKT DAPI
VEGFR3 p-ERK DAPI
LYVE1
Ki67
PCNA
Pixels

Figure S7

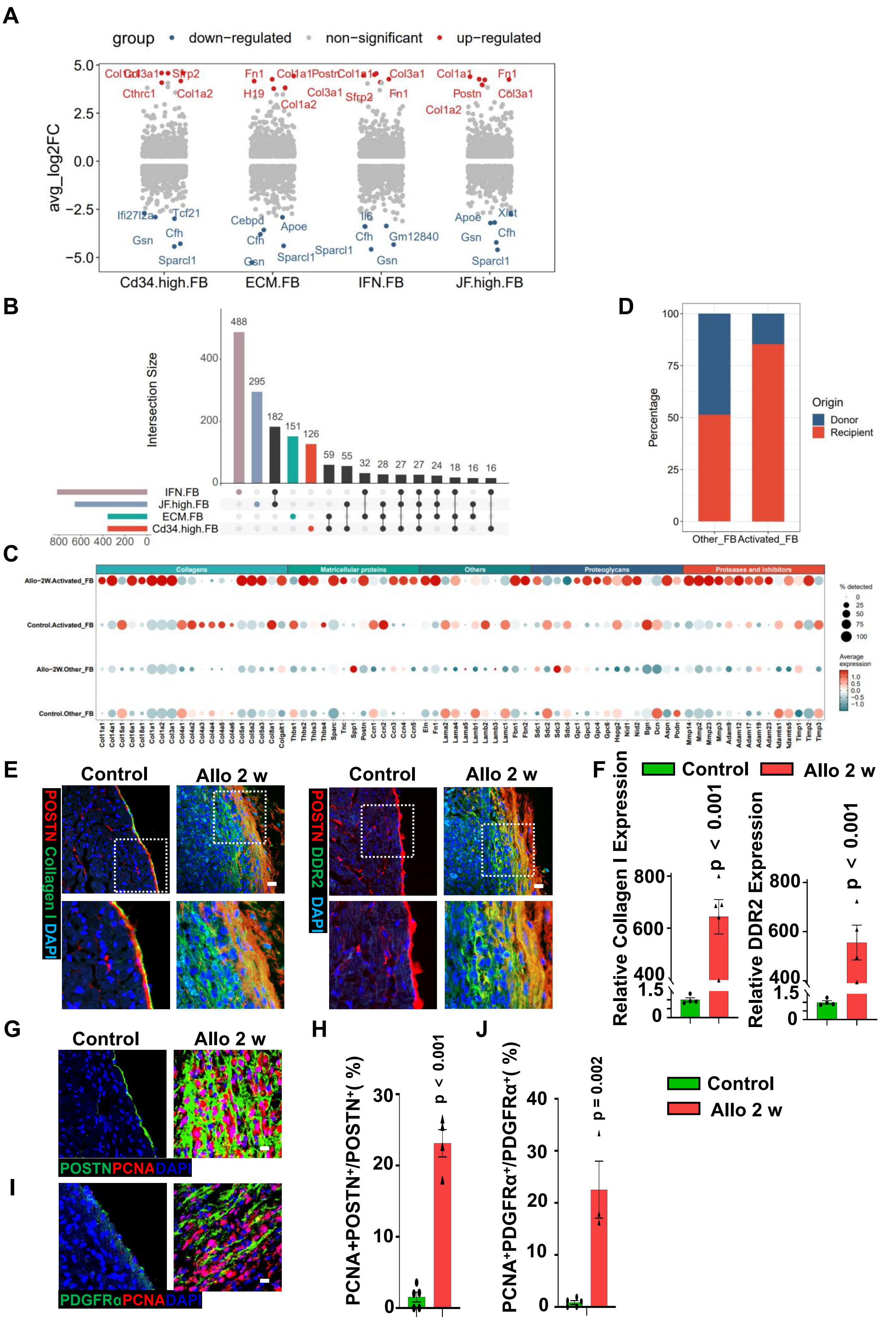
A
group down−regulated non−significant up−regulated
avg_log2FC
Cd34.high.FB
ECM.FB
IFN.FB
JF.high.FB
B
Intersection Size
IFN.FB
JF.high.FB
ECM.FB
Cd34.high.FB
C
Collagens
Matricellular proteins
Others
Proteoglycans
Proteases and inhibitors
Allo−2W.Activated_FB
Control.Activated_FB
Allo−2W.Other_FB
Control.Other_FB
D
Percentage
Origin
Donor
Recipient
Other_FB
Activated_FB
E
Control
Allo 2 w
POSTN Collagen I DAPI
POSTN DDR2 DAPI
F
Control
Allo 2 w
Relative Collagen I Expression
Relative DDR2 Expression
p < 0.001
G
POSTN PCNA DAPI
H
PCNA+POSTN+/POSTN+(%)
p < 0.001
I
PDGFRα PCNA DAPI
J
PCNA+PDGFRα+/PDGFRα+(%)
p = 0.002

Figure S8

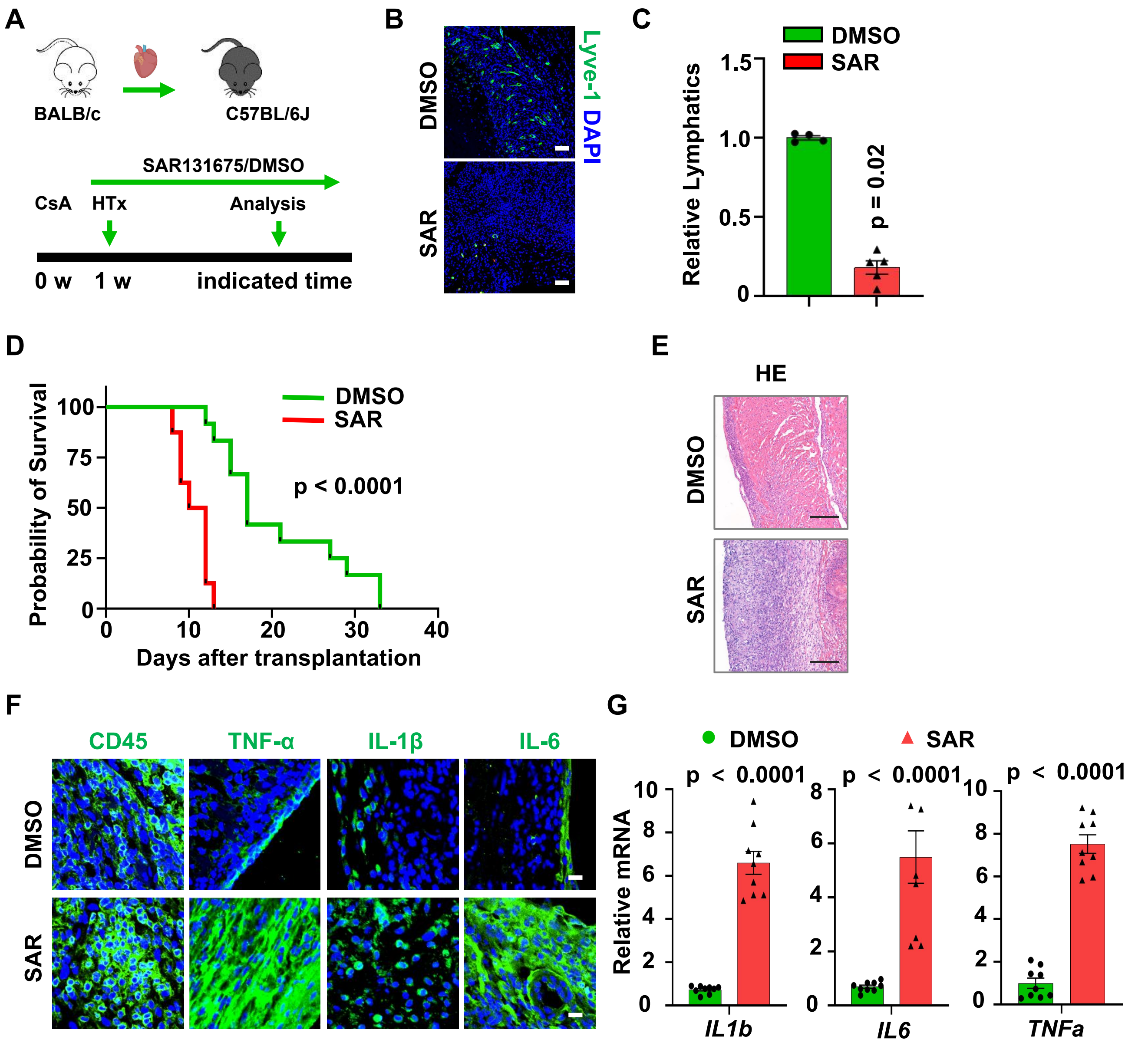

A
BALB/c
C57BL/6J
SAR131675/DMSO
CsA
HTx
Analysis
0 w
1 w
indicated time
B
DMSO
SAR
Lyve-1 DAPI
C
DMSO
SAR
Relative Lymphatics
1.5
1.0
0.5
0
p = 0.02
D
Probability of Survival
100
75
50
25
0
DMSO
SAR
p < 0.0001
0
10
20
30
40
Days after transplantation
E
HE
DMSO
SAR
F
CD45
TNF-α
IL-1β
IL-6
DMSO
SAR
G
DMSO
SAR
Relative mRNA
p < 0.0001
p < 0.0001
p < 0.0001
IL1b
IL6
TNFa

Figure S9

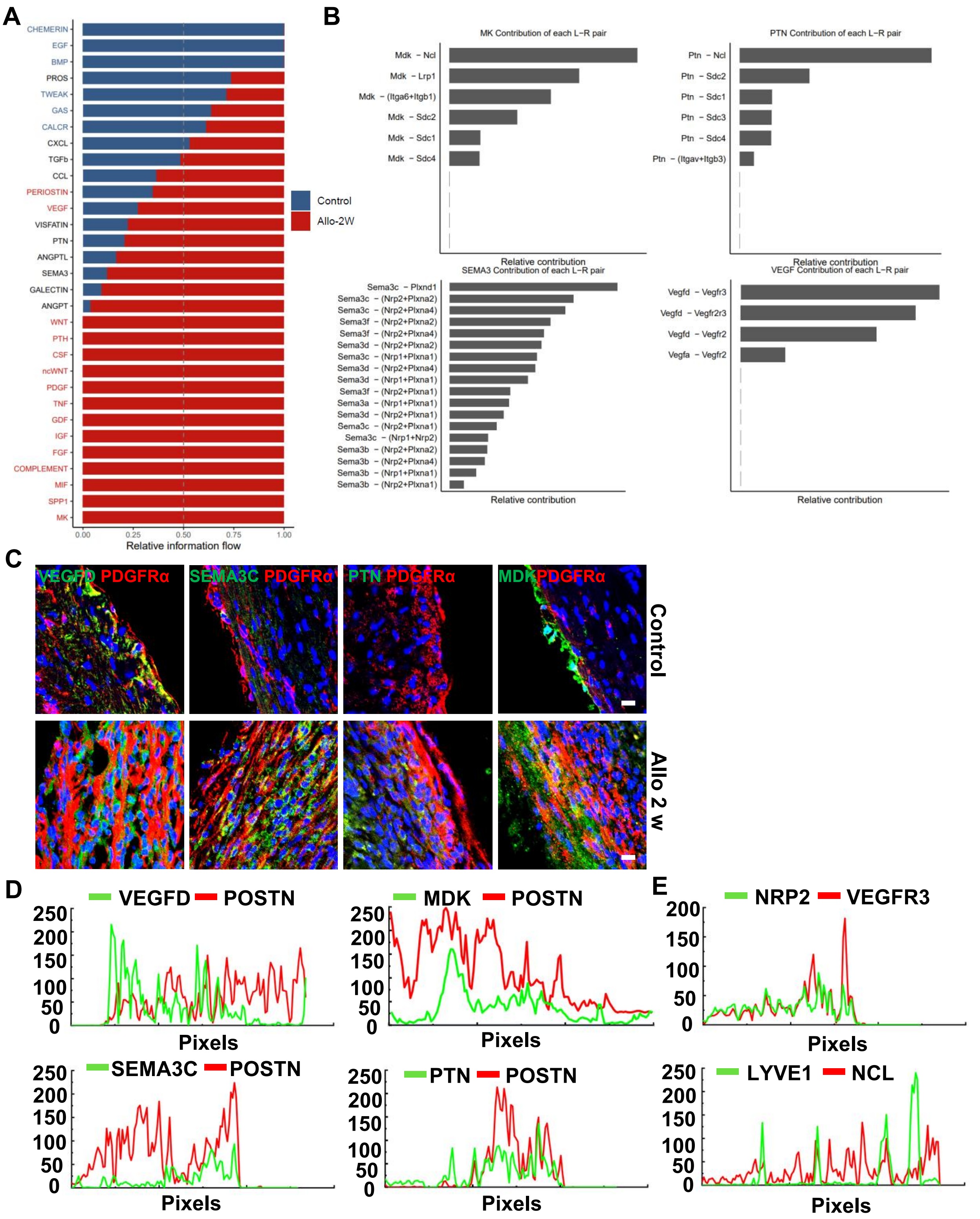

A
CHEMERIN
EGF
BMP
PROS
TWEAK
GAS
CALCR
CXCL
TGFb
CCL
PERIOSTIN
VEGF
VISFATIN
PTN
ANGPTL
SEMA3
GALECTIN
ANGPT
WNT
PTH
CSF
ncWNT
PDGF
TNF
GDF
IGF
FGF
COMPLEMENT
MIF
SPP1
MK
Control
Allo-2W
0.00 0.25 0.50 0.75 1.00
Relative information flow
B
MK Contribution of each L−R pair
Mdk − Ncl
Mdk − Lrp1
Mdk − (Itga6+Itgb1)
Mdk − Sdc2
Mdk − Sdc1
Mdk − Sdc4
Relative contribution
PTN Contribution of each L−R pair
Ptn − Ncl
Ptn − Sdc2
Ptn − Sdc1
Ptn − Sdc3
Ptn − Sdc4
Ptn − (Itgav+Itgb3)
Relative contribution
SEMA3 Contribution of each L−R pair
Sema3c − Plxnd1
Sema3c − (Nrp2+Plxna2)
Sema3c − (Nrp2+Plxna4)
Sema3f − (Nrp2+Plxna2)
Sema3f − (Nrp2+Plxna4)
Sema3d − (Nrp2+Plxna2)
Sema3c − (Nrp1+Plxna1)
Sema3d − (Nrp2+Plxna4)
Sema3d − (Nrp1+Plxna1)
Sema3f − (Nrp2+Plxna1)
Sema3a − (Nrp1+Plxna1)
Sema3d − (Nrp2+Plxna1)
Sema3c − (Nrp2+Plxna1)
Sema3c − (Nrp1+Nrp2)
Sema3b − (Nrp2+Plxna2)
Sema3b − (Nrp2+Plxna4)
Sema3b − (Nrp1+Plxna1)
Sema3b − (Nrp2+Plxna1)
Relative contribution
VEGF Contribution of each L−R pair
Vegfd − Vegfr3
Vegfd − Vegfr2r3
Vegfd − Vegfr2
Vegfa − Vegfr2
Relative contribution
C
VEGFD PDGFRα
SEMA3C PDGFRα
PTN PDGFRα
MDK PDGFRα
Control
Allo 2 w
D
VEGFD POSTN
MDK POSTN
SEMA3C POSTN
PTN POSTN
E
NRP2 VEGFR3
LYVE1 NCL
250 200 150 100 50 0
Pixels

Figure S10

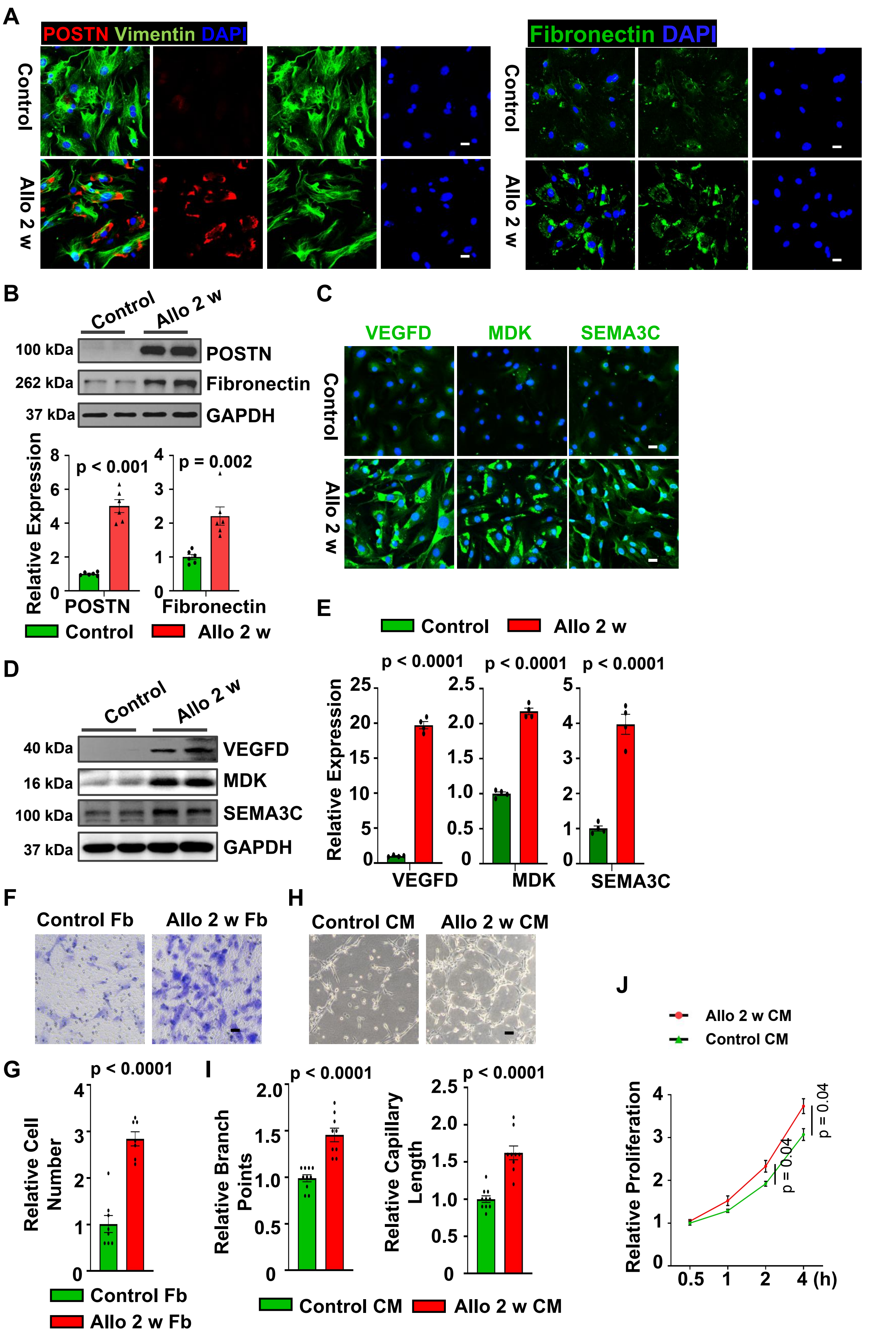

A
POSTN Vimentin DAPI
Control
Allo 2 w
Fibronectin DAPI
B
Control
Allo 2 w
100 kDa
POSTN
262 kDa
Fibronectin
37 kDa
GAPDH
Relative Expression
p < 0.001
p = 0.002
POSTN
Fibronectin
Control
Allo 2 w
C
VEGFD
MDK
SEMA3C
D
40 kDa
VEGFD
16 kDa
MDK
100 kDa
SEMA3C
37 kDa
GAPDH
E
p < 0.0001
p < 0.0001
p < 0.0001
F
Control Fb
Allo 2 w Fb
H
Control CM
Allo 2 w CM
G
Relative Cell Number
I
Relative Branch Points
Relative Capillary Length
J
Allo 2 w CM
Control CM
Relative Proliferation
p = 0.04
p = 0.04
0.5
1
2
4 (h)

Figure S11

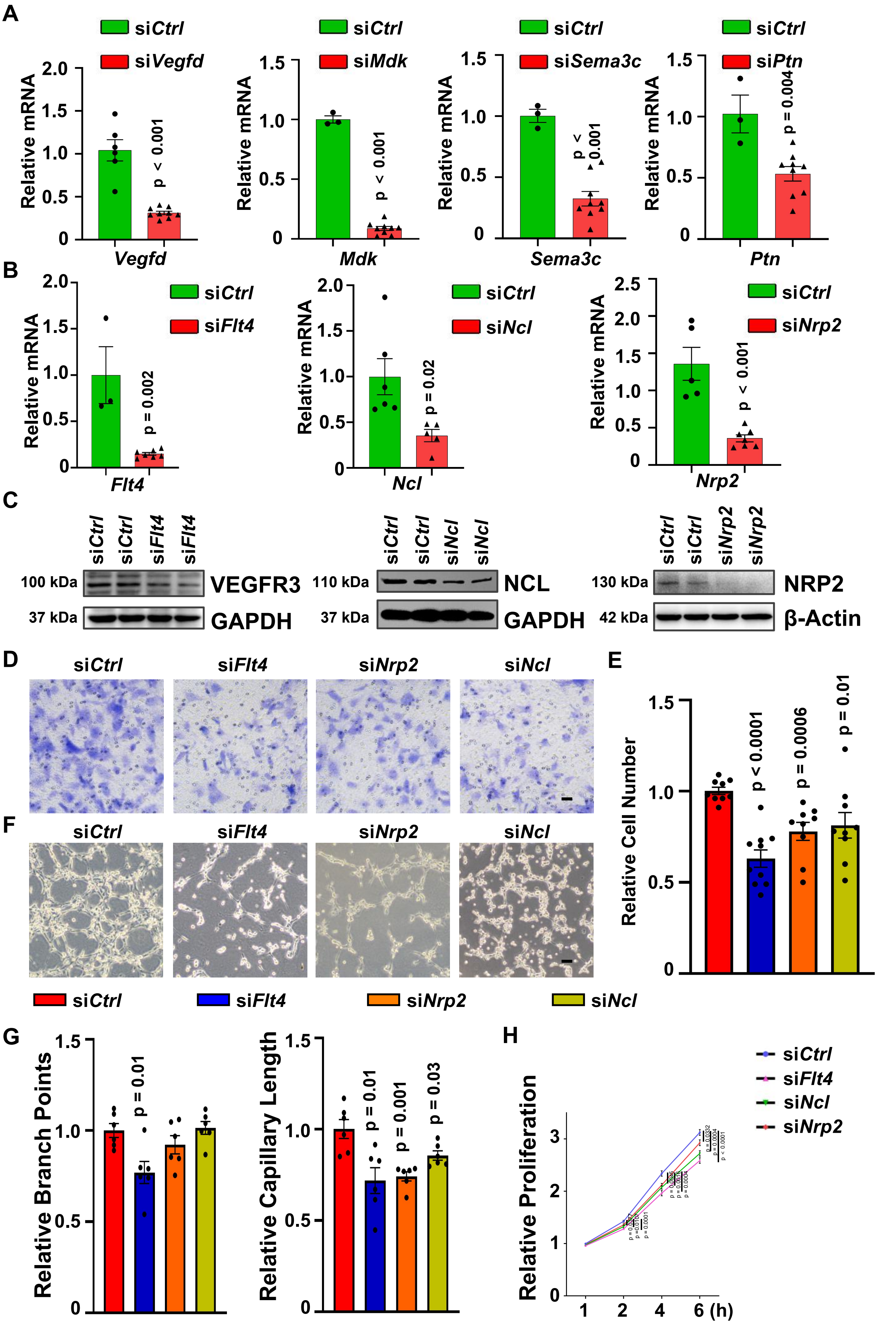
A
siCtrl
siVegfd
Relative mRNA
p < 0.001
Vegfd
siCtrl
siMdk
p < 0.001
Mdk
siCtrl
siSema3c
p < 0.001
Sema3c
siCtrl
siPtn
p = 0.004
Ptn
B
siCtrl
siFlt4
p = 0.002
Flt4
siCtrl
siNcl
p = 0.02
Ncl
siCtrl
siNrp2
p < 0.001
Nrp2
C
siCtrl siCtrl siFlt4 siFlt4
100 kDa VEGFR3
37 kDa GAPDH
siCtrl siCtrl siNcl siNcl
110 kDa NCL
37 kDa GAPDH
siCtrl siCtrl siNrp2 siNrp2
130 kDa NRP2
42 kDa β-Actin
D
siCtrl siFlt4 siNrp2 siNcl
E
Relative Cell Number
p < 0.0001
p = 0.0006
p = 0.01
F
siCtrl siFlt4 siNrp2 siNcl
siCtrl siFlt4 siNrp2 siNcl
G
Relative Branch Points
p = 0.01
Relative Capillary Length
p = 0.01
p = 0.001
p = 0.03
H
siCtrl
siFlt4
siNcl
siNrp2
Relative Proliferation
1 2 4 6 (h)

Figure S12

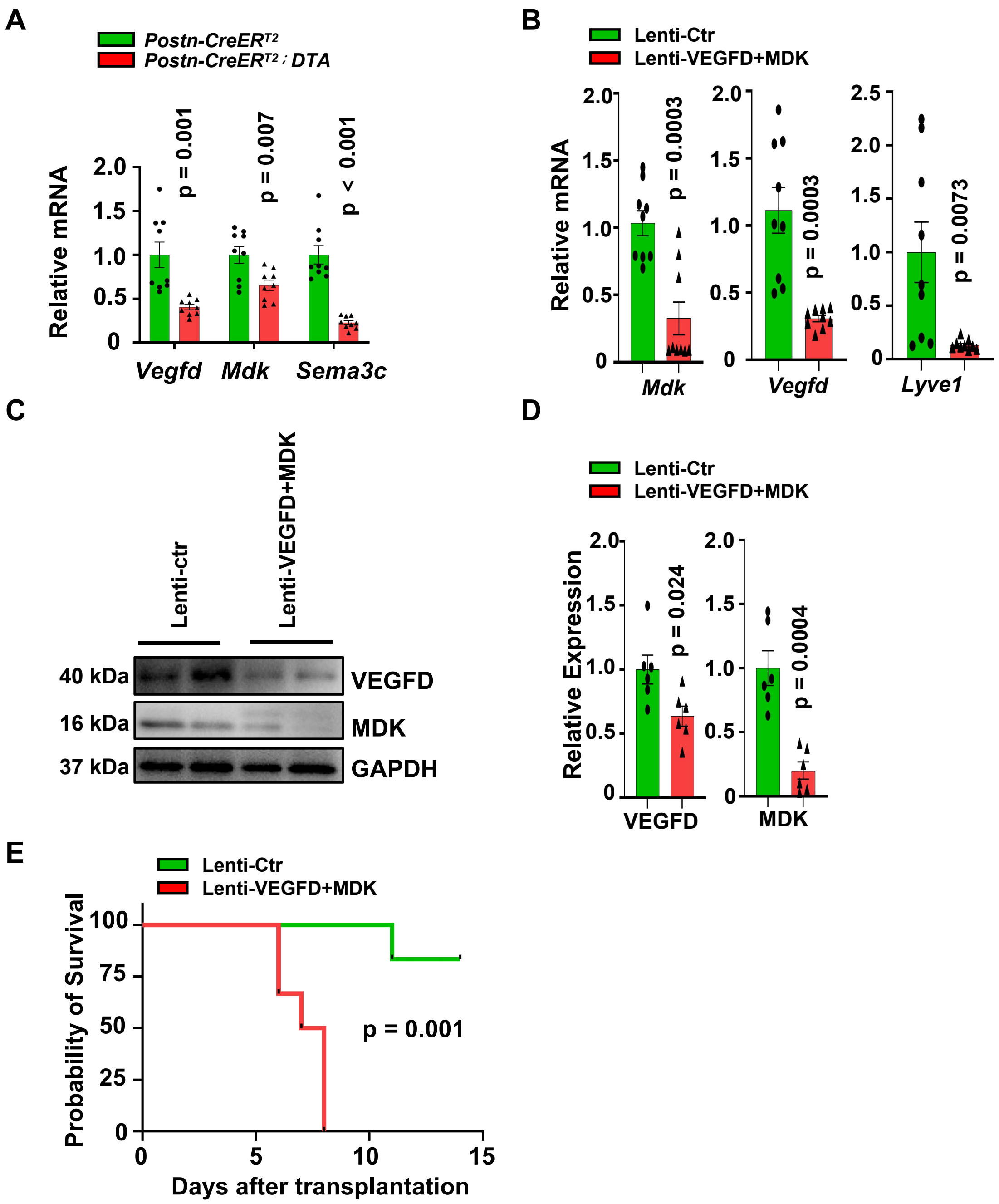
A
Postn-CreERT2
Postn-CreERT2 ; DTA
Relative mRNA
p = 0.001
p = 0.007
p < 0.001
Vegfd
Mdk
Sema3c
B
Lenti-Ctr
Lenti-VEGFD+MDK
Relative mRNA
p = 0.0003
p = 0.0003
p = 0.0073
Mdk
Vegfd
Lyve1
C
Lenti-ctr
Lenti-VEGFD+MDK
40 kDa
VEGFD
16 kDa
MDK
37 kDa
GAPDH
D
Lenti-Ctr
Lenti-VEGFD+MDK
Relative Expression
p = 0.024
p = 0.0004
VEGFD
MDK
E
Lenti-Ctr
Lenti-VEGFD+MDK
Probability of Survival
p = 0.001
Days after transplantation

Figure S13

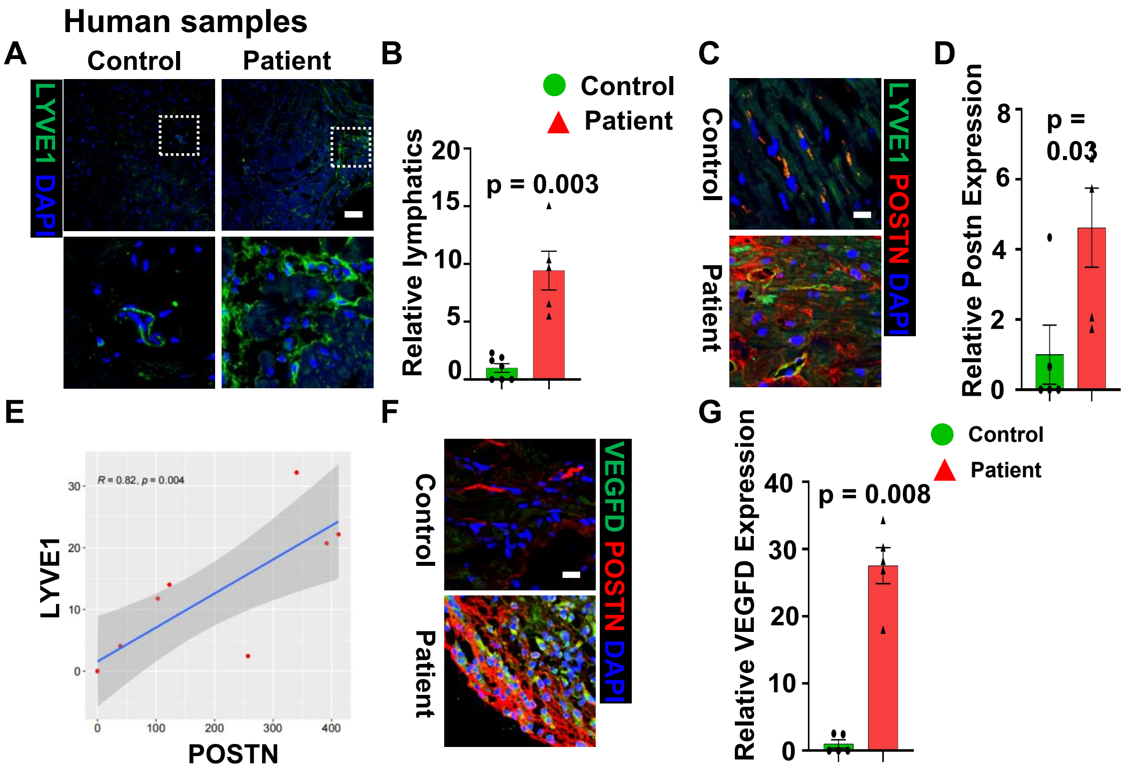
Human samples
A
Control
Patient
LYVE1 DAPI
B
Control
Patient
Relative lymphatics
p = 0.003
20
15
10
5
0
C
Control
Patient
LYVE1 POSTN DAPI
D
Control
Patient
Relative Postn Expression
p =
0.03
8
6
4
2
0
E
R = 0.82, p = 0.004
LYVE1
POSTN
30
20
10
0
0
100
200
300
400
F
Control
Patient
VEGFD POSTN DAPI
G
Control
Patient
Relative VEGFD Expression
p = 0.008
40
30
20
10
0
